# Quantifying the efficacy of genetic shifting in control of mosquito‐borne diseases

**DOI:** 10.1111/eva.12802

**Published:** 2019-06-14

**Authors:** Siyang Xia, Marissa L. Baskett, Jeffrey R. Powell

**Affiliations:** ^1^ Department of Ecology and Evolutionary Biology Yale University New Haven Connecticut; ^2^ Department of Environmental Science and Policy University of California, Davis Davis California; ^3^ Yale University New Haven Connecticut

**Keywords:** *Aedes aegypti*, assisted evolution, genetic shifting, mosquito control, quantitative polygenic model, selective breeding, vector genetics

## Abstract

Many of the world's most prevalent diseases are transmitted by animal vectors such as dengue transmitted by mosquitoes. To reduce these vector‐borne diseases, a promising approach is “genetic shifting”: selective breeding of the vectors to be more resistant to pathogens and releasing them to the target populations to reduce their ability to transmit pathogens, that is, lower their vector competence. The efficacy of genetic shifting will depend on possible counterforces such as natural selection against low vector competence. To quantitatively evaluate the potential efficacy of genetic shifting, we developed a series of coupled genetic–demographic models that simulate the changes of vector competence during releases of individuals with low vector competence. We modeled vector competence using different genetic architectures, as a multilocus, one‐locus, or two‐locus trait. Using empirically determined estimates of model parameters, the model predicted a reduction of mean vector competence of at least three standard deviations after 20 releases, one release per generation, and 10% of the size of the target population released each time. Sensitivity analysis suggested that release efficacy depends mostly on the vector competence of the released population, release size, release frequency, and the survivorship of the released individuals, with duration of the release program less important. Natural processes such as density‐dependent survival and immigration from external populations also strongly influence release efficacy. Among different sex‐dependent release strategies, releasing blood‐fed females together with males resulted in the highest release efficacy, as these females mate in captivity and reproduce when released, thus contributing a greater proportion of low‐vector‐competence offspring. Conclusions were generally consistent across three models assuming different genetic architectures of vector competence, suggesting that genetic shifting could generally apply to various vector systems and does not require detailed knowledge of the number of loci contributing to vector competence.

## 1 INTRODUCTION

Vector‐borne diseases transmitted by arthropods are major public health concerns worldwide. For example, the yellow‐fever mosquito, *Aedes aegypti,* transmits dengue fever which threatens ~40% of the world population; malaria, transmitted by *Anopheles* mosquitoes, kills millions of people each year (World Health Organization, 2014). As traditional control methods like insecticides have been losing effectiveness as well as being recognized as having adverse environmental effects, alternative approaches have been sought. One promising approach to reducing vector‐borne diseases is to genetically manipulate populations of vectors to make them less capable of transmitting pathogens (Beaty, 2000; Beerntsen, James, & Christensen, 2000; Collins & James, 1996; Hardy, Houk, Kramer, & Reeves, 1983; Scott, Takken, Knols, & Boëte, 2002). The potential of these proposals has long been discussed and is only now beginning to be seriously implemented. Many proposed modifications involve transgenic methods by which desirable genes, such as pathogen‐suppression genes, are “engineered” into the vectors (transgenic *Anopheles*: Ito, Ghosh, Moreira, Wimmer, & Jacobs‐Lorena, 2002; transgenic *A. aegypti*: Franz et al., 2006; Mathur et al., 2010). However, the public has not universally accepted such genetically modified (GM) vectors (Toure & Knols, 2006) and in many cases has concerns about their safety and ethics, which may hinder their implementation (Lavery, Harrington, & Scott, 2008; Marshall, Touré, Traore, Famenini, & Taylor, 2010; Toure & Knols, 2006).

A potential solution to this problem is found in an old method of genetic modification: selective breeding. Humans have been genetically modifying plants and animals in agriculture for centuries. Such practices utilized naturally existing genetic variations without introducing transgenes (genes or genetic material from a different species). Harking back to these traditional plant and animal breeding practices, Powell and Tabachnick (2014) suggested a nontransgene method to genetically modify vector populations. In many disease vectors, resistance to pathogens exists naturally and is genetically controlled (Black et al., 2002; Hardy et al., 1983; Severson & Behura, 2016; Tabachnick, 1994), similar to many traits selected in animal and plant breeding. Vectors resistant to transmitting pathogens have been successfully selected from wild populations with no introduction of foreign transgenes (Collins et al., 1986; Hardy, Apperson, Asman, & Reeves, 1978; Miller & Mitchell, 1991; Wallis et al., 1985). Such selected strains could be reared to large numbers and released back into the wild vector populations. The expectation is that the releases would increase the frequency of the naturally occurring alleles conferring lowered ability to transmit diseases, that is, lower vector competence (VC), which may ultimately break the disease transmission cycle (Ferguson et al., 2015; Lambrechts, 2015). The method is called “genetic shifting”: shifting frequencies of already present resistant alleles rather than introducing new genetic materials. It has several advantages over the GM methods (discussed in Powell & Tabachnick, 2014). For instance, it eliminates the controversy over transgenics and does not require a priori knowledge of the vector genome, which increases its potential for broad application.

Genetic shifting essentially represents an artificially driven migration where the immigrants drive allele frequency changes in the opposite direction as natural selection, that is, introduce a “migration load” (Lenormand, 2002; Ronce & Kirkpatrick, 2001). The theory of how migration and selection interact to determine genetic composition indicate that important drivers include the migration rate, degree of genetic differentiation between populations, strength of natural selection, order of life cycle events (migration, selection, density dependence, reproduction), and genetic architecture (Baskett, Burgess, & Waples, 2013; Lenormand, 2002; Ronce & Kirkpatrick, 2001). Quantifying how these factors determine the efficacy of genetic shifting can inform whether it is a feasible strategy in terms of the scale of the release program required (number of released individuals and program duration), the difference in vector competency between captive and wild populations that is achievable, and the life history and genetic context of the target organism. An additional consideration for genetic shifting in insect‐borne diseases is which sex to release. In many insect disease vectors such as mosquitoes, only females “bite” (take blood meals), such that male‐only releases might be more publicly acceptable. However, separating sexes requires extra resources and labor (Araújo, Carvalho, Ioshino, Costa‐da‐Silva, & Capurro, 2015) and male‐only releases may reduce efficacy of vector incompetency spread due to increased mating competition. An additional strategy could be to release both sexes but allowing them to mate and the females to feed on uninfected blood in the laboratory. This strategy is roughly equivalent to releasing eggs and larvae in addition to males.

Here, we examine the feasibility and efficacy of genetic shifting by constructing a series of coupled demographic–genetic models (Baskett et al., 2013). We explore how varying parameters of these models affect the predicted outcome, that is, how much the wild target population is reduced in VC. Variables tested include (a) genetic architecture of VC, whether highly polygenic or simple Mendelian; (b) the difference in VC between the release strain and target population; (c) size, number, and frequency of releases; (d) survival of the released strain relative to the target population; (e) “release strategy” in terms of whether only males or both sexes are released, without and with mating and blood‐feeding before releases; (f) effect of external migration; and (g) demographic effects such as density‐dependent and density‐independent survival and natural selection. We use the yellow‐fever mosquito *Ae. aegypti* as an example to parameterize our models, but the models can potentially be applied to any vector–pathogen systems, and we preform extensive parameter sensitivity analyses to assess the consistency of our conclusions across parameter values that might represent different target organisms.

## 2 MATERIALS AND METHODS

### 2.1 Model overview

The coupled demographic–genetic models follow the population size and the genetic distribution of VC in the vector population, as both influence disease epidemic risk (Focks, Brenner, Hayes, & Daniels, 2000; Scott & Morrison, 2003). The genetic distribution depends on the genetic architecture of VC, that is, the number of genes affecting the phenotype, their interactions, and their interaction with environmental variations as well as the pathogens (Severson & Behura, 2016). Such information is very limited in most vector species, and the exact genetic basis of VC may be specific to each vector–pathogen pair (Severson & Behura, 2016). Given this uncertainty, we used three different models that represent the extremes of a continuum of all possible genetic architectures on which VC sits: a quantitative polygenic model, a one‐locus Mendelian model, and a two‐locus Mendelian model. The quantitative polygenic model assumed an infinite number of loci with additive effects (i.e., the overall genetic components of the traits are the sum of contributions of all loci) on the VC genotype (Baskett et al., 2013; Turelli & Barton, 1994), with the phenotype distributed around the genotype with random environmental effects. The one‐locus and two‐locus Mendelian models assumed that one or two loci alone determine VC, with no environmental effects. In the example of *Ae. aegypti* interacting with dengue‐2 virus, the polygenic model likely resembles the genetic basis of VC better, as suggested by quantitative trait loci mapping studies (Bennett et al., 2005; Bosio, Beaty, & Black, 1998; Bosio, Fulton, Salasek, Beaty, & Black, 2000).

**Figure 1:**
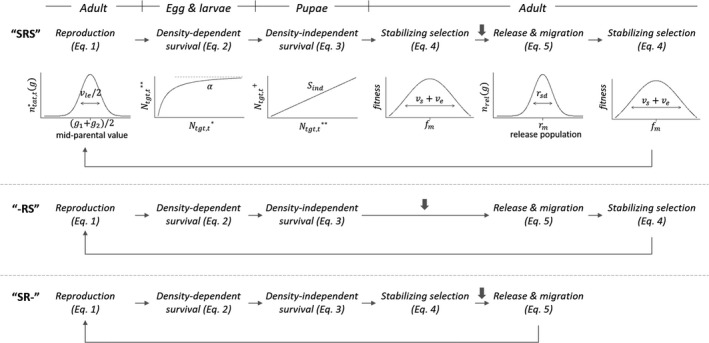
Model illustration based on *Aedes aegypti* life cycle. Each generation contains five events: reproduction that starts a new generation, density‐dependent survival in the larval stage, density‐independent survival in the pupal stage, stabilizing selection in the adult stage, and immigration and release in the adult stage. Migration from external populations happens at the same time as release. Stabilizing natural selection happens both before and after release and migration (“SRS”), only after release and migration (“‐RS”), or only before release and migration (“SR‐”). The gray downward arrows indicate the time point of censusing the population and calculating the four measures of efficacy in each life cycle order (immediately before release)

The life cycle of mosquitoes involves four stages: eggs, larvae, pupae, and adults (Christophers, 1960). In each stage, the mosquitoes experience different demographic processes (Figure 1). We assume adult mosquitoes mate randomly and produce eggs, with the inheritance of VC dependent on the genetic architecture. The offspring experience density‐dependent survival in the larval stage, which represents the feeding competition in aquatic environment with limited resources (Legros, Lloyd, Huang, & Gould, 2009; Walsh, Facchinelli, Ramsey, Bond, & Gould, 2011). In the pupal stage, density‐independent mortality occurs due to environmental factors such as habitat desiccation (Southwood, Murdie, Yasuno, Tonn, & Reader, 1972). In the adult stage, two immigration events occur: release from the laboratory‐bred population and immigration from external wild populations. We considered three different release strategies: (a) only males, (b) males and virgin females without blood‐feeding, and (c) males with females prefed with blood meals before release. In addition to release and immigration, we assume that adult mosquitoes experience stabilizing natural selection, where individuals with VC further away from the optimum in the wild have lower fitness (Sheldon & Verhulst, 1996). This is consistent with empirical studies showing intermediate levels of VC in natural populations (Bennett et al., 2002; Gonçalves et al., 2014; Souza‐Neto, Powell, & Bonizzoni, 2019). Release, immigration, and selection can occur in different orders, which can substantially alter the outcome of how the released population influences a wild population in coupled demographic–genetic models (Baskett et al., 2013; Baskett & Waples, 2013). Unfortunately, the most appropriate order is uncertain for *Ae. aegypti*. To account for this uncertainty, we explored three possible orders: (a) selection—release and migration (“SR‐”; Figure 1); (b) release and migration—selection (“‐RS); and (c) selection—releasing and migration—selection (“SRS”). We group release and migration as they influence the target population in a similar way by introducing individuals external to the target population. We assume discrete nonoverlapping generations and all individuals in the wild population have synchronized development and reproduction.

Based on this basic model framework, we simulated the change of VC distribution across the life cycle and explored an array of release schemes. We measured the efficacy of the release program using four metrics that describe the reduction of mean VC, the population size, and the population's total VC after releases. We applied sensitivity analysis to examine the relative effects of all model parameters on the release efficacy.

### 2.2 Mathematical details

Here, we describe the mathematical details of the quantitative polygenic model using the “SR‐” life cycle order and releasing both males and females without prefeeding (Figure 1). Modifications for other life cycle orders and release strategies are described in Appendix 1, and mathematical details for the one‐locus and two‐locus Mendelian models are described in Appendices 2 and 3. The VC genotype *g* of each individual ranges between 0 and 1: *g = *0 indicates that the animal is genetically predisposed to be completely incapable of transmitting pathogens (i.e., resistant), *g =* 1 indicates that it is genetically predisposed to be completely competent (i.e., susceptible), and the continuum of values between 0 and 1 represents the summed effects of the infinite number of small‐effect loci (Turelli & Barton, 1994). The model follows the population density distribution of number of individuals having genotype *g* denoted as *n_i,t_*(*g*), where *i* indicates the population of origin (*i = *rel: the release population; *i = *ext: the external population; and *i = *tgt: the target wild population) and *t* indicates generations (*t = *0, 1, 2, etc.).

Mating and reproduction happen at the end of the adult stage and produce the new generation. We separate males and females in the reproduction and release steps (first and last steps). Given random mating, the likelihood that a male and a female with genotype *g*_1_ and *g*_2 _respectively encountering each other is the product of their frequency in the population: $n_{tgt,M,t}\left(g_{1}\right)$/$N_{tgt,M,t}$ and $n_{tgt,F,t}\left(g_{2}\right)$/$N_{tgt,F,t}$, where M and F denote males and females in the population; and $N_{tgt,M,t}$ and $N_{tgt,F,t}$ are the total number of males and females: $N_{tgt,M,t}=\int n_{tgt,M,t}\left(g_{1}\right)dg_{1};N_{tgt,F,t}=\int n_{tgt,F,t}\left(g_{2}\right)dg_{2}$. The offspring genotype follows a normal probability distribution, centered at the mean genotype of the parents with variance equal to half of the genotypic variance at linkage equilibrium *v*_le_ (Turelli & Barton, 1994). Integrating the product of parental encounter probabilities and the offspring genotype distribution over all possible *g*_1_ and *g*_2_ gives rise to the offspring genotype frequency distribution. Given the number of offspring per female *R* and female population size $N_{tgt,F,t}$, the population density distribution of offspring with genotype *g* in the cases of both‐sex and male‐only releases is:

(1) $$\begin{array}{c} n_{tgt,t}^{*}\left(g\right)=RN_{tgt,F,t}\iint \frac{n_{tgt,M,t}\left(g_{1}\right)n_{tgt,F,t}\left(g_{2}\right)}{N_{tgt,M,t}N_{tgt,F,t}}\frac{1}{\sqrt{\pi v_{le}}}e^{\frac{{-\left(g-\frac{g_{1}+g_{2}}{2}\right)}^{2}}{v_{le}}}dg_{1}dg_{2}. \end{array}$$

Larvae of the next generation experience density‐dependent survival modeled by the Beverton–Holt function with the strength of density dependence *α*:

(2) $$\begin{array}{c} n_{tgt,t}^{**}\left(g\right)=\frac{n_{tgt,t}^{*}\left(g\right)}{1+\alpha N_{tgt,t}^{*}}. \end{array}$$

Pupae then go through density‐independent survival with a survival probability of *S*_ind_:

(3) $$\begin{array}{c} n_{tgt,t}^{+}\left(g\right)=S_{ind}n_{tgt,t}^{**}\left(g\right). \end{array}$$

For natural selection on VC in the adult stage, phenotypes instead of genotypes determine fitness, so we first translate the population genotype distribution into phenotypic distribution. An individual's phenotype *f* is normally distributed around its genotype *g*, with a variance equal to the environmental variance $v_{e}$. The population experiences fitness‐dependent mortality given stabilizing selection for an optimum phenotype *f*_m_ and with a selection variance (inverse of the selection strength) *v*_s_. Integrating the population density after selection over all phenotypes leads to the genotype distribution:

(4) $$\begin{array}{c} n_{tgt,t}^{++}\left(g\right)=\int e^{-\frac{{\left(f-f_{m}\right)}^{2}}{2v_{s}}}\frac{1}{\sqrt{2\pi v_{e}}}e^{-\frac{{\left(f-g\right)}^{2}}{2v_{e}}}n_{tgt,t}^{+}\left(g\right)df=\sqrt{\frac{v_{s}}{v_{s}+v_{e}}}e^{-\frac{{\left(g-f_{m}\right)}^{2}}{2\left(v_{s}+v_{e}\right)}}n_{tgt,t}^{+}\left(g\right). \end{array}$$

In the Mendelian models, we use frequency‐dependent selection to model stabilizing selection (mathematical details in Appendix 2 and 3).

Release and migration also happen at the adult stage, and in the “SR‐” scenario, after natural selection. The release population has a normal distribution of the genotype frequency $\psi_{rel}\left(g\right)$with mean VC of *r*_m_ (<*f*_m_) and standard deviation of *r_sd_*. Releases occur every *τ*_rel_ generations (e.g., when *τ*_rel_ = 2, we release every other generation). The size of release in generation *t* is a fraction $p_{rel,t}]]>$ of the size of the prerelease target population $N_{tgt,equ}$, where $p_{rel,t}=0$ when no release occurs in a generation. The release program lasts for *l*_rel_ number of releases; thus, the total number of generations over which releases occur is* τ*_rel_ × *l*_rel_. Irrespective of the VC genotypes, the released mosquitoes experience higher mortality than the wild mosquitoes due to potential laboratory domestication, leading to a relative survival probability $s_{rel}<1$. A constant number of immigrants ${N_{m}=\int n}_{ext}\left(g\right)dg$ from the external wild populations also join the target population in each generation. The external population has the same genotype frequency distribution $n_{ext}\left(g\right)$/$N_{m}$ as the prerelease target population. When both males and females are released without feeding, the population density after release and immigration is:

(5) $$\begin{array}{c} n_{tgt,t+1}\left(g\right)=n_{tgt,t}^{++}\left(g\right)+p_{rel,t}{N_{tgt,equ}s}_{rel}\psi_{rel}\left(g\right)+n_{ext}\left(g\right). \end{array}$$

We then assume the population density distribution of each sex is ${n_{tgt,M,t+1}\left(g\right)=n_{tgt,F,t+1}\left(g\right)=\frac{1}{2}n}_{tgt,t+1}\left(g\right)$ given a 1:1 sex ratio in all populations (target, external, release). Appendix S1 describes how this and other dynamics change with different release strategies.

### 2.3 Model implementation and analysis

We numerically implement the models by iterating Equations (1), (2), (3), (4), (5) in R 3.2.2 (R Development Core Team, 2016). In the quantitative polygenic model, the VC genotype range 0–1 is discretized with a step size of 2^−9^ and we apply Simpson's rule for any integral. We first demonstrate how VC genotype reduces throughout releases using the default parameter values in Table 1. These default parameter values are informed by empirical studies when available.

**Table 1:** Descriptions and values of model parameters in the quantitative polygenic model

Parameter	Description	Default	Range	Ref.^a^
*f*_m_	Optimal VC in the field	0.55	0.10–0.90^b^	(1)
*v*_p_	Total phenotypic variance of VC^c^	0.01	0.0001–0.25	(2)
*h* ^2^	Heritability of VC^c^	0.4	0.01–1	(2)
*R*	Mean number of offspring per female	40	5–150	(3)
*Α*	Beverton–Holt density‐dependent saturation constant	10^−4^	10^−5^–10^−3^	(4)
*v*_s_	Selectional variance (1/selection strength)	1	0.01–100	(4)
*S*_ind_	Density‐independent survival probability	0.7	0.2–1	AP
*N*_m_	Number of immigrants from external population	0	0–500	AP
*r*_m_	Mean VC of the release population	0.2	(0–1) *f*_m_	MD
*r*_sd_	Standard deviation of VC in the release population	0.05	(0–1)$\sqrt{v_{le}}$	MD
*p*_rel,t_	Relative size of the release at generation *t*	0.1^d^	0.01–0.5^d^	MD
*s*_rel_	Mean survival probability of released individuals	0.75	0.01–1	MD
*l*_rel_	Number of releases	20	1–50	MD
*τ*_rel_	Release frequency: number of generations between releases	1	1–5	MD
*R*_rel_	Mean number of offspring per blood‐fed released female	50	20–150	AP

a Data sources: (1) the mean VC measured from 32 wild *Ae. aegypti* populations (Bennett et al., 2002; Gonçalves et al., 2014); (2) Bosio et al. (1998); (3) Christophers (1960); and (4) because no empirical estimation is available for *Ae. aegypti*, we used the same value as in Baskett et al. (2013) and tried a broad range of values in the sensitivity analysis; AP: author opinion—these parameters depend mostly on the target population with little empirical data, so we estimated the value and range based on experience. MD: manager decision—these variables determine the scale of the release program.

b In the local sensitivity analysis, *f*_m_ ranges from 0.2 to 0.9, as *f*_m_ ≥ *r*_m_ for our model to be relevant to genetic shifting with the purpose of reducing vector competence.

c The genetic variance at linkage equilibrium $v_{le}=v_{p}h^{2}$; the environmental variance $v_{e}=v_{p}\left(1-h^{2}\right)$.

d *p*_rel,t_ = 0 when no release occurs at generation *t*.

Each simulation contains three stages: prerelease initialization, release, and postrelease recovery. In the prerelease initialization, we generate an initial VC distribution of the target population. In the quantitative polygenic model, the prerelease target population has a population distribution of *n*_tgt,0_(*g*) with mean *f*_m_, that is, centered at the optimum under natural selection. We then iterate Equations (1), (2), (3), (4), (5) and allow the distribution to reach equilibrium without releases (*p*_rel,t_ = 0). We define the equilibrium state as when the proportional change of *n*(*g*) for any genotype *g* is smaller than 10^−5^. We then start the *l*_rel_ releases each *τ*_rel_ time steps (generations). After the releases end, we follow the population for 20 more generations to examine the selection‐driven recovery of VC in the target population. Fast reduction of VC during the release stage and slow recovery during the postrelease recovery stage indicate greater efficacy. We use the same default parameter values in all three release strategies and all three life cycle orders, which allows direct comparisons. We also standardize the parameter values when possible across the polygenic models and the two Mendelian models (compare Tables 1, Table S5, and Table S13).

We summarize the release outcome with four metrics. First, we calculate the relative mean VC of the target population after releases ($\mu_{shift}$; Equation S4). This represents the shift of VC mean relative to the mean of the initial target population ${\overset{-}{g}}_{tgt,0}$ and the release population *r*_m_, with $\mu_{shift}=1$ indicating that the postrelease population has the same mean VC as the prerelease population, and $\mu_{shift}=0$ indicating that the postrelease population has the same mean VC as the laboratory‐bred release population. The second metric is the number of standard deviations shifted ($\sigma_{shift}$ ; Equation S5). This metric indicates the extent of reduction in population mean VC relative to the standard deviation of the prerelease population and therefore considers changes in both the mean and the variance of VC distribution. The third metric is the ratio of population size after release and before release ($N_{R}/N_{0}$ ; Equation S6). The last metric is the proportion of remaining integrated VC ($p_{VC}$ ; Equation S7). This is the ratio of the population's total VC after releases to that before releases. It accounts for changes of both the genotypic frequency and the population size and is likely most relevant to disease transmission (Ferguson et al., 2015; Focks et al., 2000). The equations of these metrics are in Appendix 1. Smaller $\mu_{shift}$ and $p_{VC}$ and larger $\sigma_{shift}$ indicate a greater reduction of VC in the postrelease population, that is, higher efficacy of genetic shifting. We calculated all four metrics every generation right before the release and migration step (Figure 1, gray arrows).

We perform local and global sensitivity analyses on all model parameters. For simplicity, we focus on the VC distribution at the end of the release step in all sensitivity analysis. Local sensitivity analysis (LSA) allows only one parameter value to change while keeping the others the same as in the default case. The range of each parameter is in Table 1. Although LSA directly illustrated how each parameter affects the model predictions given biologically realistic values for all other parameters, such effects likely depend on the values of other variables. To explore the relative effect of each parameter independent of other parameter values, we also perform a global sensitivity analysis (GSA) where all parameters vary simultaneously. In our GSA, we iterated the models with 100,000 combinations of parameter values randomly sampled from their ranges (Table 1). We then use Random Forest (RF), a stochastic regression and classification algorithm, to calculate importance value (“*%IncMSE*”) of all parameters in predicting each efficacy metric (Black et al., 2002; Harper, Stella, & Fremier, 2011). This parameter importance (PI) value represents the effect of a parameter after accounting for the variation of the rest of the parameters. We calculated PI using increasing numbers of simulations (20,000–100,000) to confirm that the rank of PI is relatively stable. For each set of simulations, we calculate the importance values three times. We performed the GSA simulations and RF analysis for all life cycle orders and release strategies using the same 100,000 samples of parameter values. In addition to calculating parameter importance, we also compare all four efficacy metrics directly across these different model scenarios using the Friedman rank‐sum test, and post hoc pairwise comparison using the Wilcoxon signed‐rank test, as the data do not follow a normal distribution. We repeat all LSA and GSA in the Mendelian models in addition to the quantitative polygenic models.

## 3 RESULTS

### 3.1 Default case

**Figure 2:**
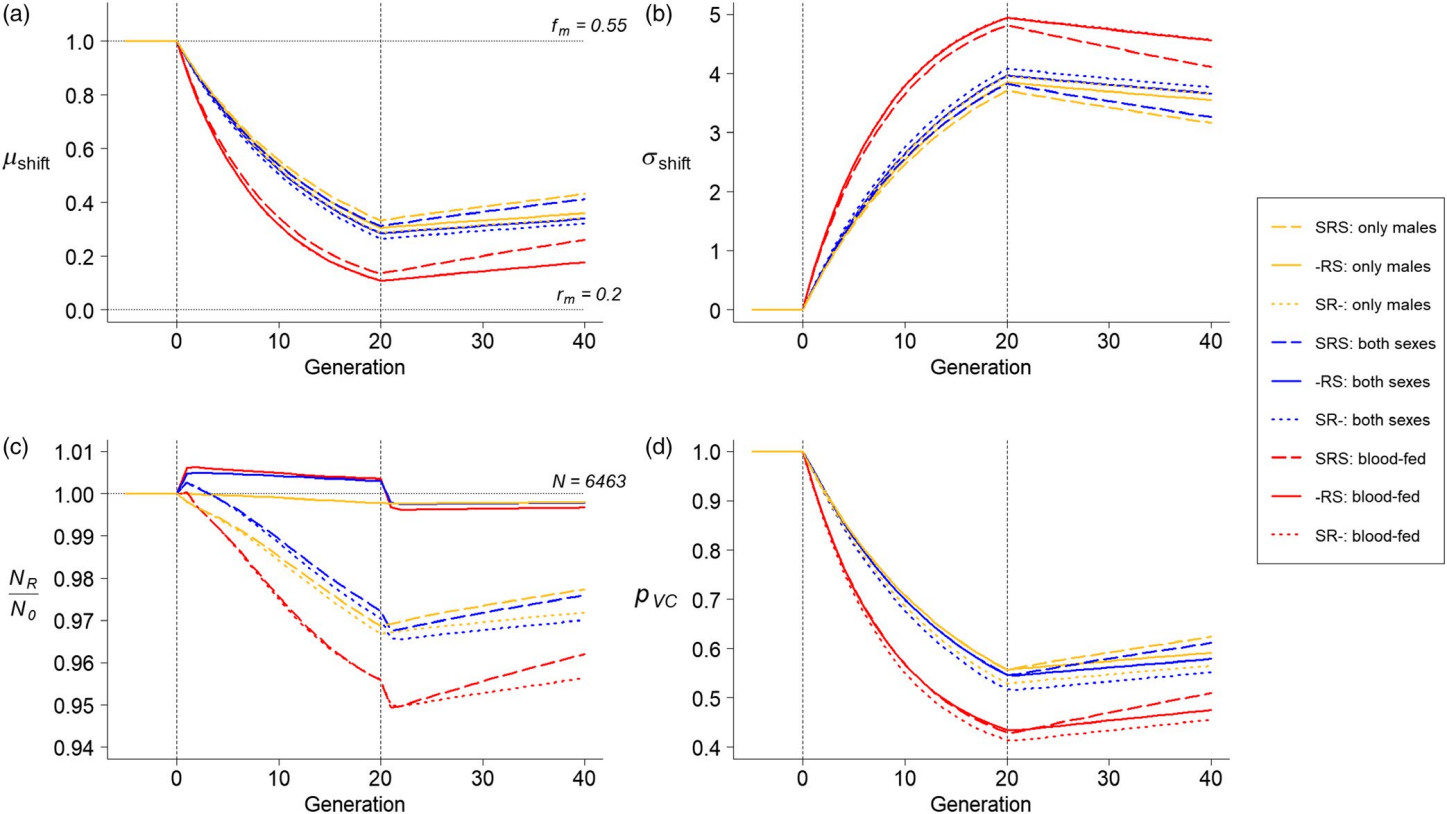
Change of VC during 20 generations of releases and 20 generations of recovery in the quantitative polygenic model. The model followed the changes of (a) relative mean of VC in the postrelease population ($\mu_{shift}$), (b) number of SDs shifted by the VC mean ($\sigma_{shift}$), (c) ratio of population size between the postrelease and prerelease population ($N_{R}/N_{0}$), and (d) the proportion of remaining integrated VC ($p_{VC}$) in the target population. Colors of the lines indicate release strategies: The yellow lines represent releasing only males, the blue lines represent releasing both sexes without blood‐feeding, and the red lines represent releasing prefed females with males. Line types indicate different orders between release and selection: The dashed, solid, and dotted lines represent the “SRS,” “‐RS,” and “SR‐” scenarios, respectively. The first and second dashed vertical lines indicate the start and the end of the releases. Five generations before the release started are also shown to demonstrate the equilibrium state of the prerelease population. The horizontal lines in (a) indicate the selection optimum (*f*_m_) and the mean VC of the release population (*r*_m_). The horizontal line in (c) indicates the size of the prerelease population. We model all scenarios using the default parameter values in Table 1. Note that the vertical axes do not start from 0 in panels (c) and (d)

In the quantitative polygenic model, across all release strategies and life cycle orders and under the default parameter values, VC in the target population decreased rapidly during the 20 releases (Figure 2). The mean VC genotype dropped from 0.55 to 0.25–0.3 depending on the scenario (Figure 2a), which equals to shifting 3–5 standard deviations of the prerelease population (Figure 2b). The integrated VC of the target population decreased by roughly half (Figure 2d). The population size had little change in all release strategies and life cycle orders (Figure 2c). The relative order of selection and release did not have a strong effect on any of the efficacy metrics in the release stage, except that population size was more stable in the “‐RS” scenario than in other scenarios. During the postrelease recovery stage, VC of the target population slowly recovered, but the population's mean VC remained at least two standard deviations lower than that of the original population for at least 80 generations, and 1 standard deviation lower for at least 160 generations (Figure S1). The target population recovered faster when selection happens both before and after release (“SRS,” dashed lines in Figures 2 and Figure S1).

In the analysis of different release strategies, releasing blood‐fed females together with males was more effective than the other two strategies, resulting in lower mean VC ($\mu_{shift}$), larger numbers of standard deviation shifted ($\sigma_{shift}$), and a smaller proportion of integrated VC remained ($p_{VC}$) (Figure 2a,b,d). Releasing only males was the least efficient strategy, but including unfed females only slightly improved the release outcome (compare yellow and red lines in Figure 2). The three release strategies were analogous in their effects on population size, which likely resulted from females having high fecundity and the total number of offspring usually exceeding the carrying capacity, such that the mosquito population size was primarily regulated by density‐dependent survival rather than reproduction. This result suggests that if strong density dependence occurs early in the life cycle, releasing females is unlikely to increase vector population size or biting rates in the long run.

The fast reduction of both populations’ mean VC and integrated VC with little change in population size, and the greater efficiency of releasing blood‐fed female, occurred for all genetic architectures (Figures S10 and S21). However, the order of selection and release had stronger effects on VC in the Mendelian models than in the quantitative polygenic models: When selection happens both before and after releasing (“SRS”), reduction of VC was slower. Compared to the single‐locus Mendelian model, this disparity among different selection–release orders was smaller in the two‐locus Mendelian model, which represents a first step toward the quantitative polygenic model.

### 3.2 Global sensitivity analysis

**Figure 3:**
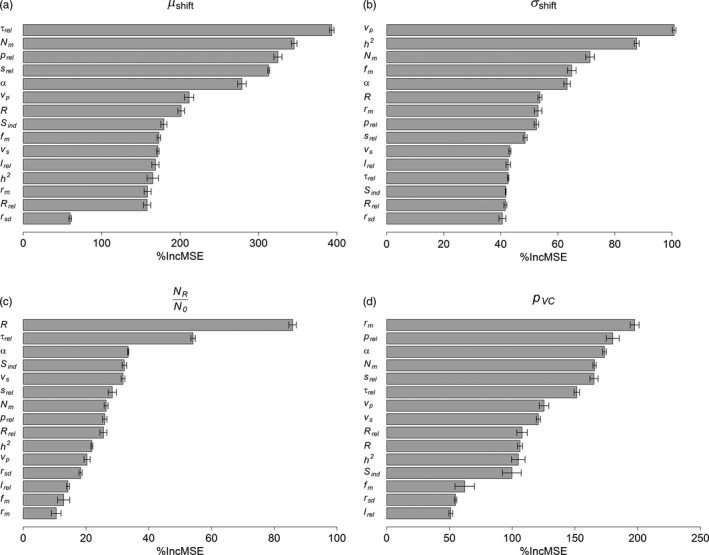
Parameter importance (PI) in determining the four efficacy metrics in the scenario “SRS: blood‐fed” through global sensitivity analysis: (a) relative mean of VC in the postrelease population ($\mu_{shift}$)**,** (b) number of SDs shifted by the VC mean ($\sigma_{shift}$), (c) ratio of population size between the postrelease and prerelease population ($N_{R}/N_{0}$), and (d) the proportion of remaining integrated VC ($p_{VC}$). The error bars represent standard errors calculated from the three replicates. Parameters are ordered decreasingly according to their PI value in each panel. PI in other scenarios is shown in Appendix 1

**Figure 4:**
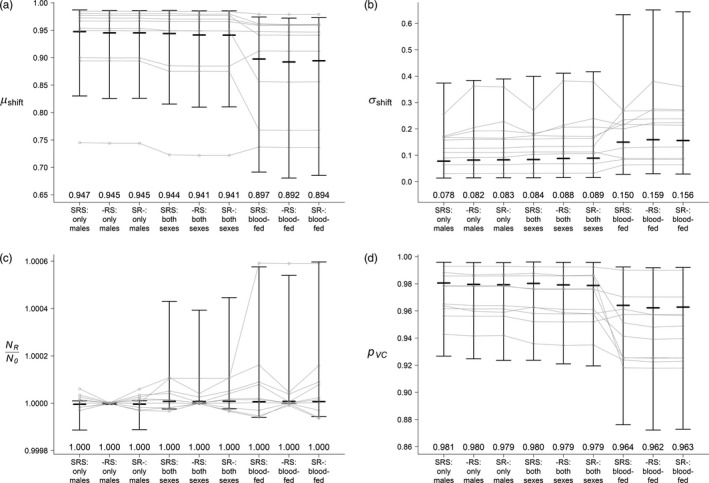
Median and interquartile range of each efficacy metric for all nine model scenarios, calculated at *t* = $l_{rel}\times \tau_{rel}$ from the 100,000 GSA simulations: (a) relative mean of VC in the postrelease population ($\mu_{shift}$)**,** (b) number of SDs shifted by the VC mean ($\sigma_{shift}$), (c) ratio of population size between the postrelease and prerelease population ($N_{R}/N_{0}$), and (d) the proportion of remaining integrated VC ($p_{VC}$). The three bars in each scenario represent the 25% quantile, the median, and the 75% quantile, respectively. The gray lines represent 10 randomly selected simulations. The numbers at the bottom of each figure show the median values of the 100,000 simulations

The proportion of integrated VC ($p_{VC}$) was most sensitive to parameters controlling the release, such as mean VC of the release population (*r_m_*), size of each release (*p*_rel_), relative survival of the released animals (*s*_rel_), and the frequency of releases (*τ*_rel_). The strength of density‐dependent survival (*α*) and the size of immigration from external populations (*N*_m_) also had strong impacts on $p_{VC}$ (Figure 3d). These parameters were also the most influential parameters in determining the relative mean VC after releases ($\mu_{shift}$) (Figure 3a), except *r*_m_, the variation of which is already accounted for in the calculation of $\mu_{shift}$ (Equation S4). This consistency between the PI ranks of $p_{VC}$ and $\mu_{shift}$ could be explained by the little change of population size through releases (Figure 4c), such that $p_{VC}$ depends mostly on the change of the VC genotype distribution indicated by $\mu_{shift}$. The number of SDs shifted ($\sigma_{shift}$) was also sensitive to density dependence (*α*) and immigration from external populations (*N_m_*), yet unlike $p_{VC}$ and $\mu_{shift}$, release‐related parameters were less influential for $\sigma_{shift}$. Instead, $\sigma_{shift}$ was more sensitive to parameters describing the distribution of VC genotypes in the original wild population, including total phenotype variance of VC (*v_p_*), heritability of VC (*h* ^2^), and optimal VC in the wild (*f_m_*) (Figure 3b). This likely results from the definition of this metric (we calculate $\sigma_{shift}$ from VC mean and variance, so by definition they strongly influence this metric). Lastly, population size ratio ($N_{R}/N_{0}$) was most sensitive to demographic parameters such as reproduction (*R*), density‐dependent survival (*α*), density‐independent survival (*S*_ind_), selectional variance (*v*_s_), and the frequency of releases (*τ*_rel_) which describes migration from the laboratory‐bred population (Figure 3c).

When comparing across release strategies and different life cycle orders, the parameter ranks of $\mu_{\mathrm{shift}}$, $\sigma_{shift}$, and $p_{VC}$ were relatively stable (Figures S2, S3, and S5). PI ranks for the population size ratio ($N_{R}/N_{0}$) were less consistent (Figure S4), possibly due to the relatively small variations of population size changes in all simulations (Figure 4c). In the Mendelian models, the PI ranks for all parameters were generally less consistent, but the general pattern remained: $\mu_{shift}$ and $p_{VC}$ were most sensitive to parameters controlling the release, and $N_{R}/N_{0}$ was most sensitive to demographic parameters (one‐locus model: Figures S11–S14; two‐locus model: Figures S22–S25). $\sigma_{shift}$ was more sensitive to release‐related parameters (e.g., *τ*_rel_) in the Mendelian models compared to that the quantitative models (comparing Figures S12, S23 and S3). The parameter ranks were relatively stable with increasing the number of simulations for all models (Figures S6, S15, and S26).

In our comparison across all nine model scenarios (combinations of release strategies and life cycle orders) using the 100,000 GSA simulations, the Friedman rank‐sum test suggested significant differences for all efficacy metrics ($\mu_{shift}$ : ${\sigma }$^2^ = 540,450, *df* = 8, *p* < 0.001; $\sigma_{shift}$: ${\sigma }$^2^ = 512,860, *df* = 8, *p* < 0.001; $N_{R}/N_{0}$: ${\sigma }$^2^ = 110,040, *df* = 8, *p* < 0.001; $p_{VC}$: ${\sigma }$^2^ = 410,700, *df* = 2, *p* < 0.001). Post hoc pairwise comparisons using the Wilcoxon signed‐rank tests with Bonferroni correction showed significant differences for all tests, except for three pairs of population size ratios (Tables S1–S4). Similar to the default simulation, releasing blood‐fed females with males was the most effective strategy (Figure 4), resulting in the lowest population mean VC ($\mu_{shift}$) and integrated VC ($p_{VC}$) and the largest number of SDs shifted ($\sigma_{shift}$). Releasing both sexes without feeding yielded a slightly better outcome than releasing only males. The order of selection and release had little effect. Neither release strategies nor life cycle order had strong effects on the change of population size, despite statistical significance: The median population size ratio was 1, with the interquartile range smaller than 0.001 (Figure 4c). Despite these general patterns across different scenarios, variations exist among individual simulations (see Figure 4, gray lines). This suggests that selecting the optimal release strategy for a specific target population may require knowledge of the local population of mosquitoes and the specific release scheme, if such information is available. The GSA results of the Mendelian models led to analogous conclusions as the quantitative polygenic models (one‐locus model: Appendix 2, Figures S11–S16, Tables [Link], [Link]; two‐locus model: Appendix 3, Figures S22–S27, Tables S14–S18).

### 3.3 Local sensitivity analysis

**Figure 5:**
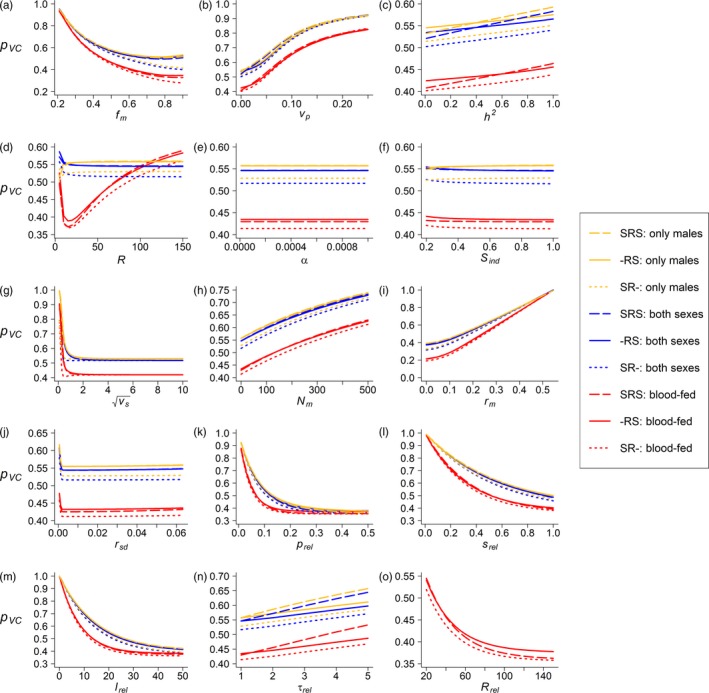
Local sensitivity analysis of the remaining proportion of integrated VC after releases ($p_{VC}$) to each parameter given all other parameters at their default values (see Table 1 for default values and ranges). Selection variance (*v*_s_) was square‐root‐transformed. Note the difference in the y‐axis values across plots. Line types and colors are as in Figure 2

The directional effects of all parameters except *f*_m_ and *r*_m_ on the release efficacy were consistent across different efficacy metrics, where lower $p_{VC}$ and $\mu_{shift}$ and higher $\sigma_{shift}$ indicate more effective releases (Figures 5, S7, and S8). Specifically, release efficacy consistently increased with the size of each release (*p*_rel,t_), the survival of the released mosquitoes (*s*_rel_), and the number of releases (*l*_rel_), but decreased with VC variance in the target population (*v_p_*), heritability of VC (*h* ^2^), number of immigrants (*N*_m_), and number of generations between two releases (*τ*_rel_). The efficacy was relatively independent of the strength of density‐dependent survival (*α*) and density‐independent survival (*S*_ind_). Increasing selectional variance (*v*_s_), that is, decreasing selection strength, increased release efficacy, but only when selection was relatively strong. Similarly, the release efficacy increased with increasing VC variance in the released population (*r*_sd_), but the effects were restricted to small *r*_sd_. The only conflicts between different efficacy metrics were observed for the optimal mean VC in the field (*f*_m_) and the mean VC of the release population (*r*_m_): Increasing *f*_m_ and decreasing *r*_m_ resulted in lower $p_{VC}$ and higher $\sigma_{shift}$ (i.e., higher efficacy), but also an increase in $\mu_{shift}$ (Figure 5a, 5i, Figures S7a, S7i, S8a, and S8i). However, this $\mu_{shift}$ increase was more likely explained by the calculation of this metric (Equation S4), rather than a true indicator of decreasing release efficacy. Larger *f*_m_ and smaller *r*_m_ increase the disparity between the prerelease target population and the release population, which leads to an increase of $\mu_{shift}$ for the same amount of absolute reduction of mean VC. Therefore, for *f*_m_ and *r*_m_, $p_{VC}$ and $\sigma_{shift}$ are more informative metrics than $\mu_{shift}$.

For population size ratio ($N_{R}/N_{0}$), the LSA showed that parameters that increase release efficacy tend to reduce population size (Figure S9), but this pattern was scenario‐dependent. Overall, none of the parameters strongly increase or decrease population size as $N_{R}/N_{0}$ rarely exceeded 1.02 or dropped below 0.9, except for reproduction (*R*), selectional variance (*v*_s_), and the optimal mean VC in the wild (*f*_m_). Selection variance, *v*_s_, showed a nonmonotonic effect on population size (Figure. S9g), which was also observed in Ronce and Kirkpatrick (2001). When selection is weak, fitness‐dependent mortality is low, and the population size is likely regulated by density‐dependent survival instead of selection. On the other hand, when selection is very strong, it could effectively eliminate the effects of the releases. Only with an intermediate selection strength can the wild population accumulate migration load, which then decreases population size.

The order of release and selection had little effect on the release efficacy ($\mu_{shift}$, $\sigma_{shift}$, and$p_{VC}$) (Figures 5, S7, and S8). In comparison, the change of population size, $N_{R}/N_{0}$, was more sensitive to the life cycle order (Figure S9). When selection happens only after releases (“‐RS”), the population size is relatively constant across the ranges of most parameters. This was mainly caused by the model setup where we censused the population before releases, and in the “‐RS” scenario that is right after the density‐dependent and independent survival which determine the population size and mask the effects of selection or releases on the population size.

Regarding release strategies, releasing blood‐fed females showed the highest efficacy across most parameter values (Figure 5, S7, and S8), which is consistent with the default simulations and the GSA. The only exception is the reproductive output per female (*R*). *R* had little effect on model output when releasing only males or releasing both sexes without feeding, yet when blood‐fed females are released, increasing *R* decreased the release efficacy (Figure 5d). We assumed that the released blood‐fed females would produce a different number of offspring (*R*_rel_) from the wild females, so increasing *R* reduced the relative contribution of the released females to the next generation, hence resulting in a reduction in efficacy. Correspondingly, increasing *R*_rel_ while holding *R* constant increased the release efficacy (Figure 5o). Population size was robust among different release strategies (Figure S9), which again suggested that releasing females does not have strong demographic consequences.

LSA in the one‐locus and two‐locus Mendelian model reached similar conclusions as in the quantitative polygenic model (Appendices 2 and 3: Figures S17–S20 and S28–S31). The optimal allele frequency in the wild population (*A*_w_, *A*_w1_, *A*_w2_) and the allele frequency of the release population (A*_r_*, A*_r1_*, A*_r2_*) had similar effects on release efficacy as *f*_m_ and *r*_m_ in the quantitative polygenic model. A noticeable difference between the Mendelian models and the quantitative polygenic model was that *R* and *S*_ind _had larger effects on the release efficacy (Figures S20 and S31), possibly due to their larger effects on population size (Figure S19 and S30). The strength of frequency‐dependent selection (*s*_fd_) also had a stronger and more linear effect on release efficacy, compared to that of the selectional variance (*v*_s_) in the quantitative polygenic model (comparing Figure 5g and Figures S20f and S31h). This suggests that the mechanism of selection in the wild may play a role in determining the outcome of the release program. Unique to the Mendelian models, the dominance of the susceptible alleles (*d*, *d*_1_, *d*_2_) had a negative effect on release efficacy; that is, increasing *d*, *d*_1_, or *d*_2_ resulted in less reduction of VC. This is expected as *d*, *d*_1_, and *d*_2_ directly determine the VC of heterozygotes in the mosquito population.

## 4 DISCUSSION

Our models predict that releasing mosquitos selected to have lowered ability to transmit pathogens can rapidly reduce vector competence of the wild vector populations. With the best‐estimated parameter values, with one release per generation, in just 20 generations (where insect vectors can have generation times on the scale of a month) the population's mean VC decreased by more than three standard deviations and the integrated VC of the entire population decreased by roughly half. Significant reduction persists for about 80 generations after releases are ended (~7 years) with some levels of reduction persisting for 160 generations (~13 years). The reduction was consistent across models assuming different genetic architectures of vector competence (quantitative polygenic model or Mendelian models) and was insensitive to different timing of the releases relative to the natural selection. These results suggested that genetic shifting could be effective in reducing disease transmission capability of wild mosquito populations with modest time and resource investment, and implementation does not rely heavily on understanding the genetic architecture of vector competence, which is currently unavailable for most arthropod vectors.

### 4.1 Determinants of release frequency

Both local and global sensitivity analyses suggested that parameters directly related to the management of the release program had strong effects on the efficacy of the releases, especially for the proportion of the population's integrated VC remained after releases ($p_{VC}$). High sensitivity of $p_{VC}$ to the mean VC of the release population (*r*_m_), the size of each release (*p*_rel_), the survival rate of the released mosquitoes (*s*_rel_), and the frequency of releases (*τ*_rel_) (Figure 3d) suggests that, empirically, these four parameters require the most attention in management decisions. *r*_m_ and *s*_rel_ largely depend on the result of selective breeding prior to the releases, and their effects on the release efficacy were relatively linear (Figure 5i, 5l). Therefore, maximizing the efficacy of the selective breeding program (i.e., deriving a release strain with the lowest competency possible while minimizing adverse fitness effects like inbreeding) is critical for the success of later releases. The size of each release (*p*_rel_) and the release frequency (*τ*_rel_), on the other hand, determine the scale of the release program. *τ*_rel_ had moderate PI ranks in the quantitative polygenic model and a top rank in the Mendelian models with a linear effect on $<![CDATA\left[p_{VC}\right]]>$ indicating that when resources are available, releasing mosquitoes as frequently as possible can enhance program success. Despite the importance of *p*_rel_, LSA suggested a diminishing effect which saturates around 0.3 (i.e., 30% of the size of the wild population).

How large of a release is feasible depends on the target population size. For example, the effective population size (*N*_e_) of wild *Ae. aegypti* ranges from as low as 50 up to about 600 (Endersby et al., 2011; Olanratmanee et al., 2013; Rašić et al., 2015; Saarman et al., 2017). These estimates are for local demes that may extend over only a few hundred meters consistent with the low migration behavior of this mosquito (Harrington et al., 2005; Maciel‐De‐Freitas, Codeco, & Lourenco‐De‐Oliveira, 2007; Muir & Kay, 1998). Measured census population sizes range from about 1,000 (Lounibos, 2004) to 5,500 (Carvalho et al., 2015). Rearing a release population of a few thousand every month is practical for most facilities and mosquito research laboratories (Zhang et al., 2018). The other parameter describing the scale of the release program is the duration (*l*_rel_) of releases. Its relatively weak and diminishing effect on $p_{VC}$ suggested that releases can be effective in a relatively short period (~20 releases) and program managers have the flexibility to adjust the duration according to resource availability without strongly influencing the outcome.

When comparing different release strategies with regard to which sex to release, releasing prefed females together with males always yielded the highest efficacy of reducing the target population's VC. This high efficacy likely resulted from the fact that prefed females are already mated before released and ready to lay eggs, so they are more likely to contribute to the next generation before experiencing the stabilizing selection in the adult stage and other sources of mortality. Because of the low survival rates of mosquito adults in the wild (Brady et al., 2013; Harrington et al., 2001; Maciel‐De‐Freitas et al., 2007; Reiter, 2007) and the low VC of the released females, we do not expect this strategy to significantly increase the disease transmission compared to releasing only males. Simulations of the effect of releasing blood‐fed females on disease transmission confirm this and will be the subject of a forthcoming publication. Another advantage of releasing prefed females is that the optimal rearing environment likely increases the fecundity of these females (*R*_rel_), which can further increase the efficacy of releases (Figure 5o). Between the other two release strategies, the slightly lower efficacy of releasing only males is expected, because releasing only males biases sex ratio and intensifies mating competition, which reduces the likelihood of reproduction for the released males. We did not consider strategies in which only females (with or without blood‐feeding) are released, as separating sexes requires more resources (Araújo et al., 2015) and the public is less likely to accept an all‐female release.

In addition to parameters and decisions related to the release scheme, characteristics of the target population and the local environments also influenced the release efficiency and therefore inform decisions on whether it is time‐ and resource‐efficient to implement a genetic shifting program in a particular locality or for a particular vector. The relatively high importance of immigration (*N*_m_) and density dependence (*α*) for all efficacy metrics (Figure 3) suggests that these two parameters are most crucial to consider before implementing the release programs. The importance of immigrants from external populations was also shown in a spatially explicit model by Okamoto, Robert, Lloyd, and Gould (2013). For *Ae. aegypti*, release programs will be more effective in isolated mosquito populations, such as on islands or in rural villages, than in well‐connected populations like in large cities. On the other hand, the small N*_e_* and short dispersal range for this species suggest a metapopulation structure with relatively small and geographically limited demes, that is, relatively isolated subpopulation. This implies that releases of even of a few hundred mosquitoes spaced over an area at a short interval (e.g., ~300 m) could potentially be effective in larger areas like cities.

The effect of density‐dependent survival in the larval stage on release efficacy is less straightforward, because the LSA indicated that changing *α* alone had little effect (Figure 5e). This disparity suggests that density dependence may influence release outcomes through interactions with other life cycle events, such as natural selection, or it may show stronger effects under different values of other parameters. The potentially complex effects of density‐dependent survival are suggested in Gomulkiewicz, Holt, and Barfield (1999). These potential interactions may also cause the RF analysis in the GSA to underestimate the importance of some parameters, which could partially explain the unexpected low parameter importance of selection strength. Under default parameter values of all other parameters, only very strong selection (i.e., small *v*_s_) diminished release efficacies (Figure 5g). We are unaware of any empirical evidence supporting such strong selection pressure on vector competence in mosquitoes. In addition, if there is strong selection against incompetent mosquitoes, one would not expect the high level of these individuals (10%–70%) often observed in populations (e.g., Gonçalves et al., 2014; Souza‐Neto et al., 2019). Therefore, natural selection on mosquitoes might not hinder the efficacy of genetic shifting.

Lastly, our comparison across different life cycle orders and genetic architectures (Mendelian models vs. the quantitative polygenic model) of VC did not find large effects on release efficacy in most cases, except that selection had stronger effects in Mendelian models. They also did not alter how each parameter influences the release outcomes. Therefore, implementing genetic shifting is not likely to require detailed knowledge on the number of loci that contribute to VC. In sum, among all parameters characterizing the wild population and the habitat, measuring the strength of density‐dependent survival and the gene flow from external populations will help to accurately predict release outcomes and to evaluate whether a target population is suitable for genetic shifting.

### 4.2 Release as migration

The releases in our model resemble one‐way migrations from the selective‐breeding population with lower VC into the target population with higher VC. In a generic model of migrations between two populations experiencing stabilizing selection for different trait values, Ronce and Kirkpatrick (2001) showed that increasing difference between the two populations’ optima, increasing migration rate, and decreasing strength of selection increased maladaptation in the receiving population (“migration load”) and decreased the equilibrium population size. Although our model focused on the short‐term changes of the phenotype instead of the equilibrium states as in Ronce and Kirkpatrick (2001), we observed similar effects: Decreasing VC of the breeding population (i.e., distance between the two populations’ optima), increasing release size (i.e., migration rate), and decreasing selection strength resulted in a higher number of SDs shifted by the population VC mean (a proxy for maladaptation, sensu Ronce & Kirkpatrick, 2001) and a lower population size (Figures S8 and S9). However, in contrast to the generic model predicting accumulation of maladaptation (migration load) (Lenormand, 2002; Ronce & Kirkpatrick, 2001), our models did not predict a large reduction in the population size. This difference may be explained by the relatively high reproductive rate typical of small insects like mosquitoes (*R* ranged between 5 and 150) in our models compared to that in the generic model (*R* ≈ 1) (Ronce & Kirkpatrick, 2001). This high productivity allowed the population to reach carrying capacity in just one generation, even when mortality from the maladaptation was strong.

Previous migration‐load models demonstrated that the outcome of migration depended largely on the timing of migration relative to selection, reproduction, and density dependence. Migration has the greatest effect on population size and phenotypic distribution when it happens after selection within a single generation (Baskett et al., 2013; Baskett & Waples, 2013; Ronce & Kirkpatrick, 2001). Consistent with these results, we observed a similar pattern in our model as the “SR‐” scenario showed slightly higher efficiency than the other two life cycle orders. However, the disparities among different orders were minor in our model compared to similar coupled genetic–demographic models with one‐way migration (Baskett et al., 2013), possibly due to the weaker selection pressure and the larger genetic variance assumed in our models, which reduced the efficiency of selection in removing maladapted individuals. Empirical studies of VC in wild mosquito population usually report larger variance (Kristine E. Bennett et al., 2002; Gonçalves et al., 2014; Gubler & Rosen, 1976; Souza‐Neto et al., 2019), and no evidence has supported very strong natural selection on VC in the wild. Therefore, in the case of genetic shifting for disease vectors, the time of releases within a life cycle may be less important.

The outcomes of releases (i.e., migrations) not only depend on the timing of releases but also which sex and life stage are released. To our knowledge, this model is the first to examine the effect of sex‐dependent dispersal on the migration load. Sex‐biased dispersal is common in nature; for example, in mammals it is usually the males who disperse, while in birds female dispersal is more common (Greenwood, 1980; Handley & Perrin, 2007). Our model provided an example that sex‐dependent migration could alter the expectation of migration load, particularly when coupled with the life history of the species (here, blood‐fed females vs. unfed females). Incorporating sex‐biased migration into models could refine model predictions and even provide new insights into our understanding of natural systems.

### 4.3 Model assumptions

Predictions from our model inevitably depend on model assumptions, so it is important to examine them to assess the generality and accuracy of our conclusions. First, for the genetic architecture of vector competence, we assumed either an infinite number of genes with additive small effects (the quantitative polygenic model) or one or two Mendelian genes. They represent the two extreme genetic architectures, and consistency among the three models implied that our conclusions are likely to hold with the actual genetic architecture of VC in various vectors. However, uneven contribution of genes (i.e., genes of major and minor effect), dominance, and epistasis, ignored in our model, may affect the speed of adaptation and therefore may influence the interaction between selection and migration (Gomulkiewicz, Holt, Barfield, & Nuismer, 2010). Future models incorporating more complex genetic architectures could potentially increase the accuracy of quantitative predictions. We also assumed random mating in all models. Relaxing this assumption may reduce release efficiency if released individuals experience mating discrimination from the target population possibly due to favoring local adaptation (Baskett et al., 2013; Lenormand, 2002). However, genetic shifting selects the release strain from the local wild populations, which reduces the risk of mating discrimination by wild mosquitoes and ensures high local fitness (Powell & Tabachnick, 2014). Therefore, assortative mating is not likely to be strong. Thirdly, we assumed nonoverlapping generations while wild mosquitoes have overlapping generations. In another study, Yang et al. found that including overlapping generations by adding age structure did not substantially alter the expected demographic and genetic dynamics for a wild population experiencing input from a captive population ([Link]). This provides indirect support that conclusions from our model will likely hold when considering overlapping generations. Furthermore, the model lacks demographic stochasticity or mutation. Débarre, Ronce, and Gandon (2013) explored these factors in a model featuring two habitats connected by migration and showed that both population density and mean trait values were robust to demographic stochasticity. Mutation had little effects on trait values but could alter population density. However, their model focused on the equilibrium states while our model examined the effects of releases in the short term (< 50 generations), during which mutation in the vectors is not likely to accumulate. Lastly, we assumed that migration from external populations and releases always occur at the same time in the adult stage (Figure 1). In reality, mosquitoes often migrate in other life stages such as eggs, particularly assisted by human transportation. Our model predicted that releasing blood‐fed females, which essentially approximates releasing eggs, reduced VC of the target population more than releasing unfed females and males. Likewise, immigrants from external populations could have larger effects if they come as eggs or larvae instead of adults. Therefore, investigations into how different life stages of immigrants may influence release efficacy warrant future study.

Relaxation of other model assumptions may provide insights into additional management approaches to further increase the efficacy of genetic shifting. For example, we assumed that the field conditions remain consistent throughout the period of releases. In reality, most mosquito populations experience seasonality resulting in fluctuating population size due primarily to temperature fluctuations and/or marked rainy and dry seasons (Scott et al., 2000). Our models predicted that the releases will be more efficient during the dry/cold season when the target population is small (release cohorts represent a larger percentage of the target population; Figure 5k). The likelihood of fixation of the resistant alleles is also predicted to be higher with smaller target populations (Okamoto et al., 2013). Analogous to taking advantage of seasonality, traditional vector control methods can actively reduce the wild population size. However, such measures may have undesirable outcomes. For example, using insecticides to reduce populations prior to or during releases is less likely to promote the efficacy of genetic shifting because the release strain would need to be resistant to the insecticide used, which would then introduce insecticide resistance into the target population. However, methods such as standing water reduction can both reduce the wild population and increase the efficacy of genetic shifting to synergistically enhance overall vector control efficacy.

Another model assumption is that we treated the target population as a single homogeneous group and ignore spatial structure, yet empirically a large target population may actually contain several subgroups (Maciel‐De‐Freitas et al., 2007; Muir & Kay, 1998; Olanratmanee et al., 2013). This could hinder the efficacy of releases as resistance is constrained in a subset of the target population. A spatially explicit model like Skeeter Buster (Magori et al., 2009) could provide guidance on selecting the optimal release approaches, such as multiple local releases instead of a single or few concentrated releases (Magori et al., 2009). Additionally, our models only explored the change of VC distribution over a few years or tens of generations. After the release program stopped, natural selection will drive the VC back toward the optima (Figure 2). Future models monitoring the long‐term efficacy of release and incorporating additional actions, such as low‐frequency maintenance releases, can greatly extend the longevity of effectiveness of the control program.

Finally, we did not consider the possible interaction between different pathogen genotypes and different vector genotypes, that is, GxG interactions (Lambrechts, 2011; Lambrechts, Fellous, & Koella, 2006; Severson & Behura, 2016). The existence of multiple pathogen genotypes could undermine the efficacy of genetic shifting, especially if the GxG interactions undermine the ability to select for low VC to multiple pathogen strains; for example, lower competency to one pathogen genotype leads to higher competency to another. If different pathogen genotypes occur in different locations, an effective program would require selecting the release strain from mosquitoes collected from the target population as well as using a pathogen for selection isolated from the target area (Powell & Tabachnick, 2014). Whether the selected strain would have reduced VC for pathogens not specifically selected for, either different genotypes of the same pathogen or other pathogens, is another unknown. GxGxE (environmental) interactions would be important if the environment in the target area changed or the releases were moved to a different locality. In addition to GxGxE interactions, we ignored the possible adaptation of pathogens to the increase of resistance in the vector population during releases, which could diminish the efficacy of releases. One could potentially reduce the effects of pathogen adaptation by regularly renewing the release population of vectors, that is, repeating the selective breeding during the course of releases using the newly evolved pathogen as the selective agent. Additional models incorporating the coevolution between vectors and pathogens could further increase the accuracy in predicting the outcome of genetic shifting.

### 4.4 Broad application of genetic shifting

Our model provided theoretical support for the potential to use genetic shifting in reducing the capacity of a wild mosquito population to transmit diseases, but how does it compare to other vector control approaches? One of the most promising and successful proposals is to use the intracellular bacteria *Wolbachia* (Caragata, Dutra, & Moreira, 2016; Hoffmann et al., 2011). Infection of *Wolbachia* reduces disease capacity of the mosquitoes and also introduces cytoplasmic incompatibility (CI) which provides frequency‐dependent advantages for infected female mosquitoes over wild females (Barton & Turelli, 2011; Caragata et al., 2016; Turelli, 2010; Turelli & Hoffmann, 1991). This CI‐induced advantage was hoped to potentially allow the *Wolbachia* infection, and thus resistance to pathogens, to spontaneously spread in the wild populations. This would be a major improvement from most existing mosquito control approaches, including genetic shifting, which suffer from the lower fitness of the released mosquitoes in the wild. The example of a successful establishment of *Wolbachia* in Australia also demonstrated that it is a practical approach for persistent control of disease transmission (Hoffmann et al., 2011; Schmidt et al., 2017). However, *Wolbachia* infection also comes with a fitness cost of lower viability and fecundity of the mosquitoes (McMeniman & O'Neill, 2010; Ross, Endersby, & Hoffmann, 2016). The ratio of CI‐induced reproductive advantage and infection‐induced fitness cost determines a frequency threshold only above which *Wolbachia* infection can be stabilized in the target population (Barton & Turelli, 2011; Schmidt et al., 2017; Turelli, 2010; Turelli & Barton, 2017). This limits the spread of the infection (Schmidt et al., 2017). In contrast, our results suggest that genetic shifting does not have a strong threshold effect, rendering it a more general mosquito control approach. Furthermore, genetic shifting does not rely on the host–symbiont association with characteristics of *Wolbachia*, so the method is easier to transfer to different vectors.

The models explicated here could also be coupled with *Wolbachia* infection to achieve an even higher efficacy of vector control. For instance, achieving the threshold frequency for *Wolbachia* infection in the target population requires initial releases of laboratory bacteria‐infected mosquitoes into the target population, a process similar to releasing selectively bred mosquitoes with low VC. Incorporating our models could potentially provide useful information for optimizing the initial release steps. In addition, the concept of genetic shifting could be applied to selectively breed mosquitoes that have a higher susceptibility to *Wolbachia* infection, lower fitness cost, and stronger dispersal ability, and release them back to modify the target population to further facilitate the establishment of *Wolbachia*.

Although in the current study we primarily focused on *Ae. aegypti* control, our models can be easily adapted to assess release programs for other disease vectors, such as *Anopheles* mosquitoes and tsetse flies *Glossina spp*., as long as we know their basic life history and empirical measurements of a few key parameters such as density‐dependent survival (Powell & Tabachnick, 2014). More broadly, the idea of genetic shifting and the models we presented here can apply with careful adaptations to any situation where modification of certain traits in the wild population is desired. For example, in rescuing endangered species, conservation biologists and managers can potentially select for resistance to environmental disturbance (e.g., climate change) or to diseases afflicting the endangered species, and then release them back to increase the population's overall resilience. This has been called “assisted evolution,” where one suggested use is to improve corals’ survival in rising sea temperature (van Oppen, Oliver, Putnam, & Gates, 2015). Kelly and Phillips (2016) also proposed a framework of “targeted gene flow” to assist conservation of endangered species, which involves moving individuals with beneficial traits (e.g., stress resistance) to areas under threat (also termed “assisted gene flow” in the specific context of climate change; Aitken & Whitlock, 2013). This idea is similar to genetic shifting but focuses on utilizing variance across populations in different geographic areas, instead of enhancing the within‐population variance as proposed here. A case study suggested it as a promising method to reduce the negative effects of invasive toxic toads on native *Dasyurus hallucatus* (northern quolls) in Australia (Kelly & Phillips, 2018). It is worth noting that applying genetic shifting or targeted gene flow in conservation may have different goals as well as different challenges than in vector control. Instead of aiming to increase a possible maladaptation and decrease population size in the wild vector populations, conservation programs usually desire the opposite. Preserving genetic diversity may also be important and, in conservation management, the source and target populations may be more sensitive to demographic changes and stochasticity due to small population sizes. Moreover, releases and translocation of individuals may be more expensive and limited by resources. These diverse goals and challenges encourage further research to carefully assess the efficacy of genetic shifting in each specific application, both theoretically using similar coupled genetic–demographic models as the one we presented and empirically by pilot experiments.

## CONFLICT OF INTEREST

None declared.

## DATA ARCHIVING STATEMENT

Because this is a modeling paper, we did not collect or store raw data.
