# Supplementary material for: Quantifying the efficacy of genetic shifting in control of mosquito‐borne diseases
Source: Evol Appl. 2019 Jun 14;12(8):1552–68. doi: 10.1111/eva.12802 (PMC6708429; doi:10.1111/eva.12802)
Supplement: Supplementary file 1 [file EVA-12-1552-s001.docx]

**Appendix 1:** **Supplementary mathematical details, figures and tables of the quantitative polygenic model**

*Mathematical details*

Here we describe modifications made to the mathematical details to accommodate different life cycle orders and release strategies. We model different orders of selection and release by rearranging Equation 4 and 5 as in Figure 1. Models for different release strategies vary in the reproduction step (Equation 1) and the release and migration step (Equation 5). We use the same equations to model density-dependent survival (Equation 2), density-independent survival (Equation 3), and stabilizing selection (Equation 4) for all release strategies.

When releasing only males, reproduction is the same as releasing both sexes without feeding (Equation 1). In the release and migration step, we assume a 1:1 sex ratio in the target and the external population but 100% males in the release populations. Males and females are model separately:

$$\begin{aligned} n_{tgt,M,t+1}\left( g \right)=\frac{n_{tgt,t}^{++}\left( g \right)}{2}+ p_{rel,t} {N_{tgt,equ} s}_{rel} \psi_{rel}\left( g \right)+\frac{n_{ext}\left( g \right)}{2},\# \left( S1-1 \right) \end{aligned}$$

$\begin{aligned} n_{tgt,F,t+1}\left( g \right)=\frac{n_{tgt,t}^{++}\left( g \right)}{2}+ \frac{n_{ext}\left( g \right)}{2}.\# \left( S1\text{-}2 \right) \end{aligned}$We replace Equation 5 with Equation S1 when modeling this release strategy.

In the scenario where both males and pre-fed females are releases, we model reproduction differently. Releasing blood-fed females roughly equals to releasing their offspring, as the females are ready to lay eggs. The per-capita reproductive output of blood-fed females is *R_rel_* ≥ *R* as the pre-fed females feed better in the lab and are expected to have slightly larger fecundity than their wild counterparts. Because the females mate within the lab-bred population, we assume that the offspring of the released females have the same genotype frequency distribution as the release population: $\psi_{rel}\left( g \right)$. Assuming we release $N_{rel,F}=\int n_{rel,F}(g)$ females each time, the combined offspring population density distribution for the blood-fed female release strategy is:

$$\begin{aligned} n_{tgt,t}^{*}\left( g \right)=R N_{tgt,F,t}\iint\frac{n_{tgt,M, t}\left( g_{1} \right)}{N_{tgt,M, t}}\frac{n_{tgt,F, t}\left( g_{2} \right)}{N_{tgt, F,t}} \frac{1}{\sqrt{\pi v_{le}}} e^{-\frac{{-\left( g-\frac{g_{1}+g_{2}}{2} \right)}^{2}}{v_{le}}}dg_{1}dg_{2}+ R_{rel}N_{rel,F}\psi_{rel}\left( g \right).\#\left( S2 \right) \end{aligned}$$

In the release step, we assume a 1:1 sex ratio of all populations but keep track of released females separately from the females in the target population, as they have different reproductive contribution (Equation S2). Therefore, the population densities after each release and immigration under this release strategy is:

$$\begin{aligned} n_{tgt,M,t+1}\left( g \right)=\frac{n_{tgt,t}^{++}\left( g \right)}{2}+ \frac{p_{rel,t}}{2} {N_{tgt,equ} s}_{rel} \psi_{rel}\left( g \right)+\frac{n_{ext}\left( g \right)}{2},\# \left( S3\text{-}1 \right) \end{aligned}$$

$$\begin{aligned} n_{tgt,F,t+1}\left( g \right)=\frac{n_{tgt,t}^{++}\left( g \right)}{2}+ \frac{n_{ext}\left( g \right)}{2},\# \left( S3\text{-}2 \right) \end{aligned}$$

$\begin{aligned} n_{rel,F}\left( g \right)= \frac{p_{rel,t}}{2} {N_{tgt,equ} s}_{rel} \psi_{rel}\left( g \right).\# \left( S3\text{-3} \right) \end{aligned}$When implementing the model for this release strategy, we replace Equation 1 with Equation S2, and replace Equation 5 with Equation S3.

Lastly, we summarize the outcome of each simulation using the four efficacy metrics:

$\begin{aligned} \mu_{shift}=\frac{\bar{g}_{tgt,l_{rel}\times\tau_{rel}} - r_{m}}{\bar{g}_{tgt,0} - r_{m}} ,\#\left( S4 \right) \end{aligned}$

$\begin{aligned} \sigma_{shift}=\frac{\bar{g}_{tgt,0} - \bar{g}_{tgt,l_{rel}\times\tau_{rel}}}{\sigma_{tgt,0}} ,\#\left( S5 \right) \end{aligned}$

$\begin{aligned} \frac{N_{R}}{N_{0}}=\frac{N_{tgt,l_{rel}\times\tau_{rel}}}{N_{tgt,0}},\#\left( S6 \right) \end{aligned}$

$\begin{aligned} p_{VC}=\frac{\int g n_{tgt,l_{rel}\times\tau_{rel}}\left( g \right)dg}{\int{g n}_{tgt,0}\left( g \right)dg}, \#\left( S7 \right) \end{aligned}$

where $g$ represents VC genotype. $\bar{g}_{tgt,t}$ is the mean genotype in the target population at generation t ($\bar{g}_{tgt,t}=\int gn_{tgt,t}\left( g \right)dg/N_{tgt,t}$) and $\sigma_{tgt,0}$ is the standard deviation of the VC distribution in the pre-release population ($\sigma_{tgt,0}=\sqrt{\int\left( g-\bar{g}_{tgt,0} \right)^{2}n_{tgt,0}\left( g \right)dg/N_{tgt,0}}$).

*Supplementary figures and tables*
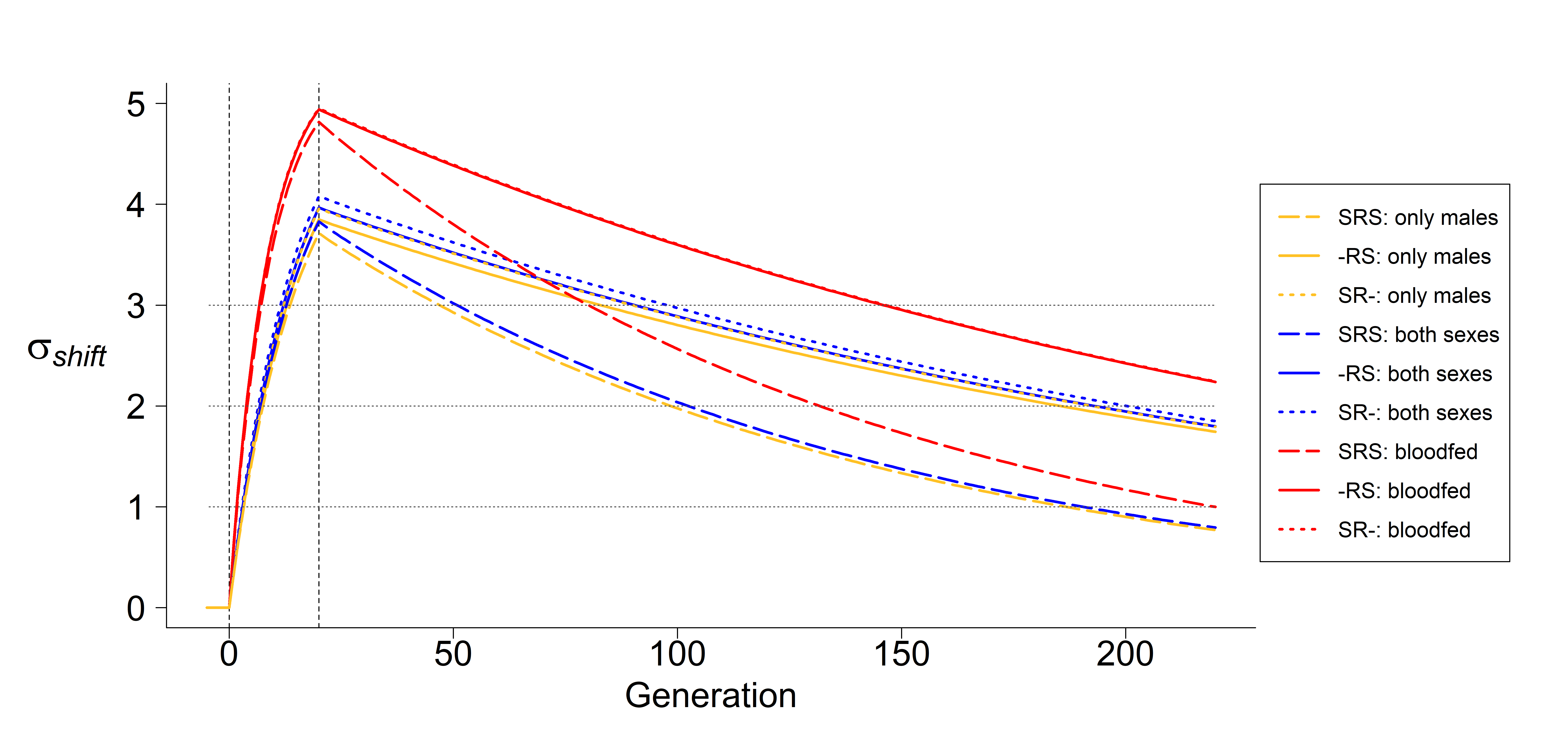


**Figure S1.** Number of SDs shifted by the VC mean ($\sigma_{shift}$) during 20 generations of releases and 200 generations of recovery in the quantitative polygenic model. Line types and colors are as in Figure 2. The first and second dashed vertical lines indicate the start and the end of the releases. Five generations before the release started are also shown to demonstrate the equilibrium state of the pre-release population. We model all scenarios using the default parameter values in Table 1.

**
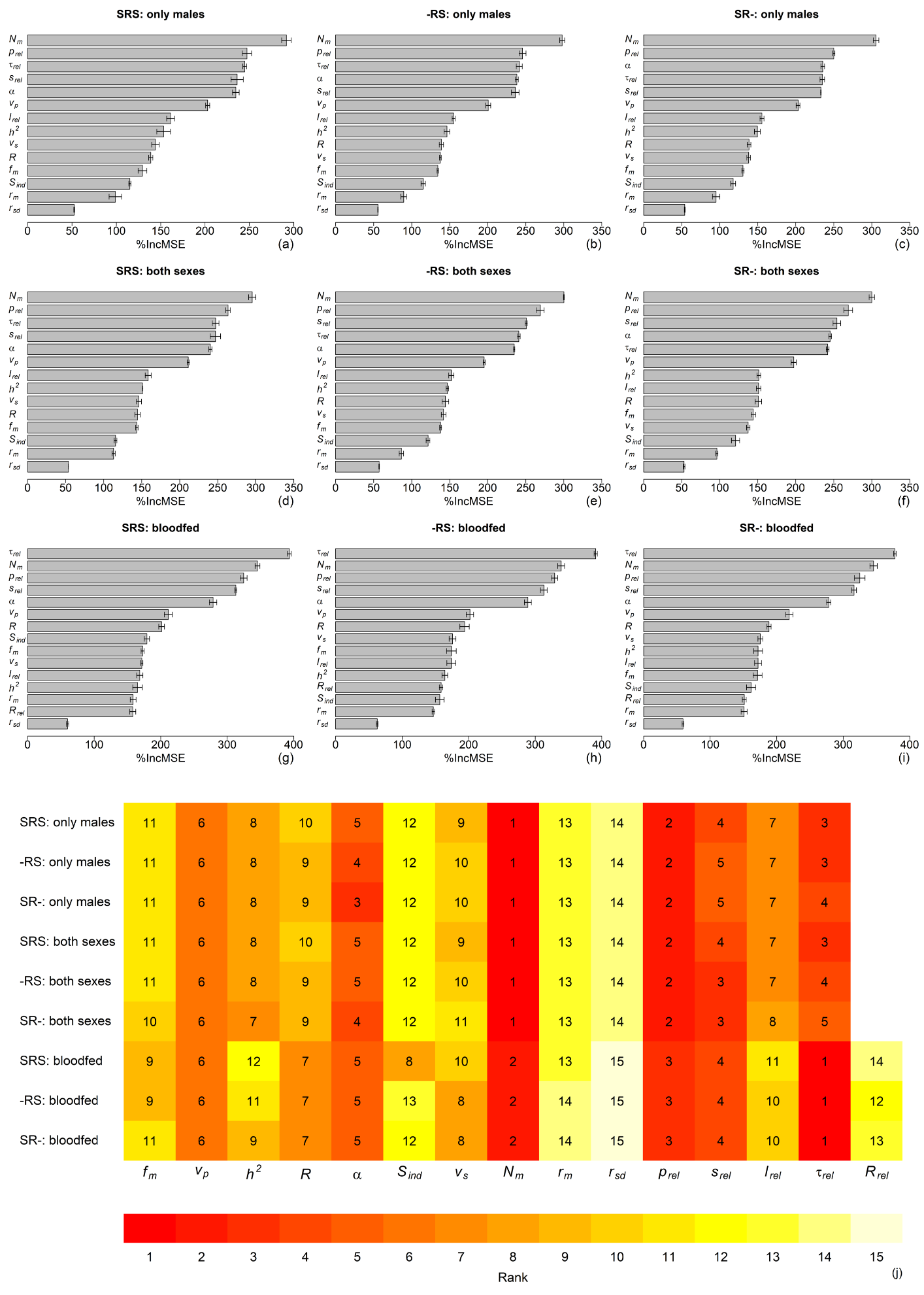
**

**Figure S2.** Parameter importance (PI) in determining the relative mean of VC in the post-release population ($\mu_{shift}$) in all nine model scenarios in the quantitative polygenic model. (a)-(i) PI values of all parameters in each scenario. The error bars represent the standard errors calculated from the three replicates. Parameters are ordered decreasingly according to their PI value in each panel. (h) Heat plot of PI ranks in all nine scenarios. Ranks are shown as numbers in grids as well as colors.

**
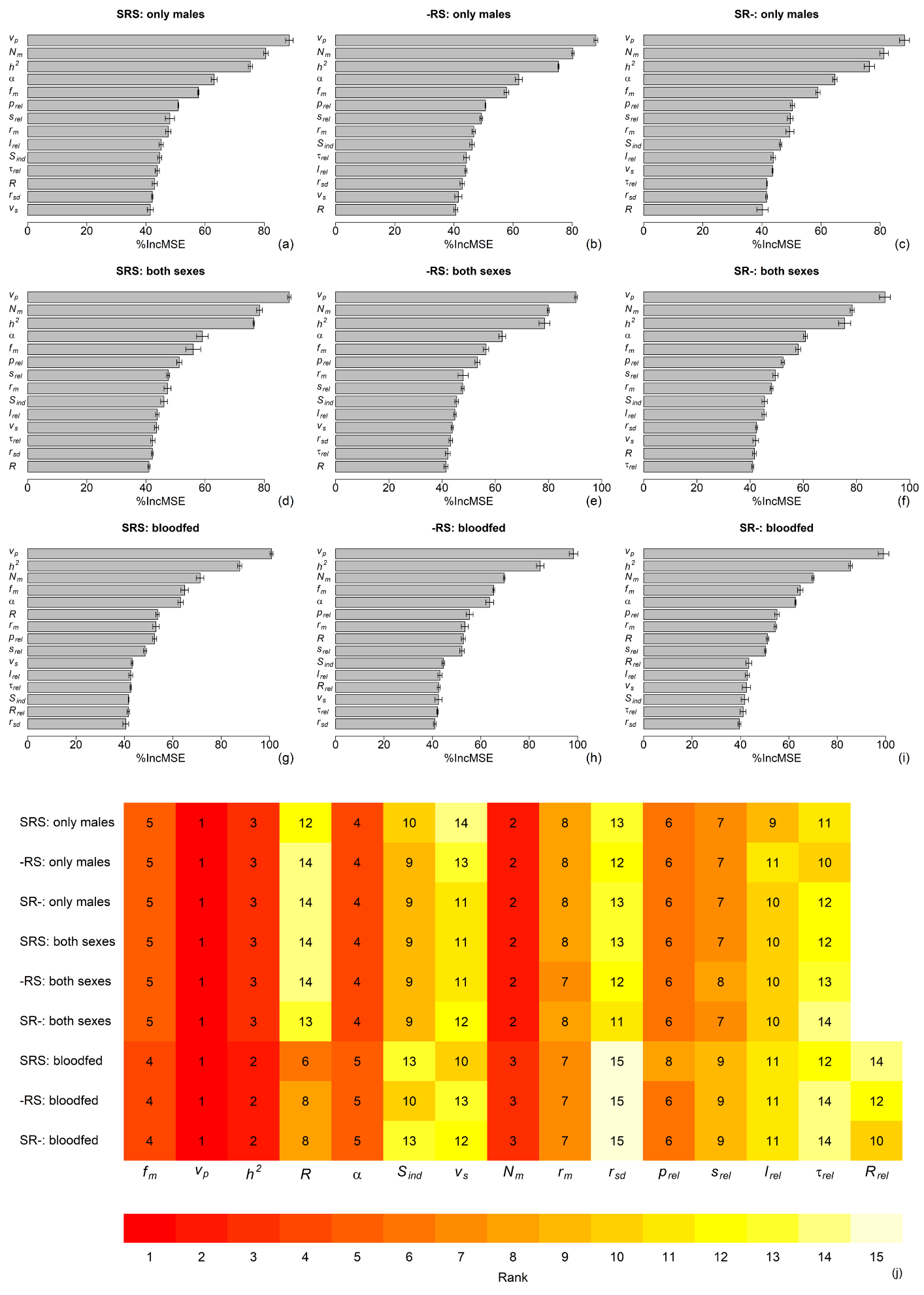
**

**Figure S3.** Parameter importance (PI) in determining the number of SDs shifted by the VC mean ($\sigma_{shift}$) in all nine model scenarios in the quantitative polygenic model. (a)-(i) PI values of all parameters in each scenario. The error bars represent the standard errors calculated from the three replicates. Parameters are ordered decreasingly according to their PI value in each panel. (h) Heat plot of PI ranks in all nine scenarios. Ranks are shown as numbers in grids as well as colors.

**
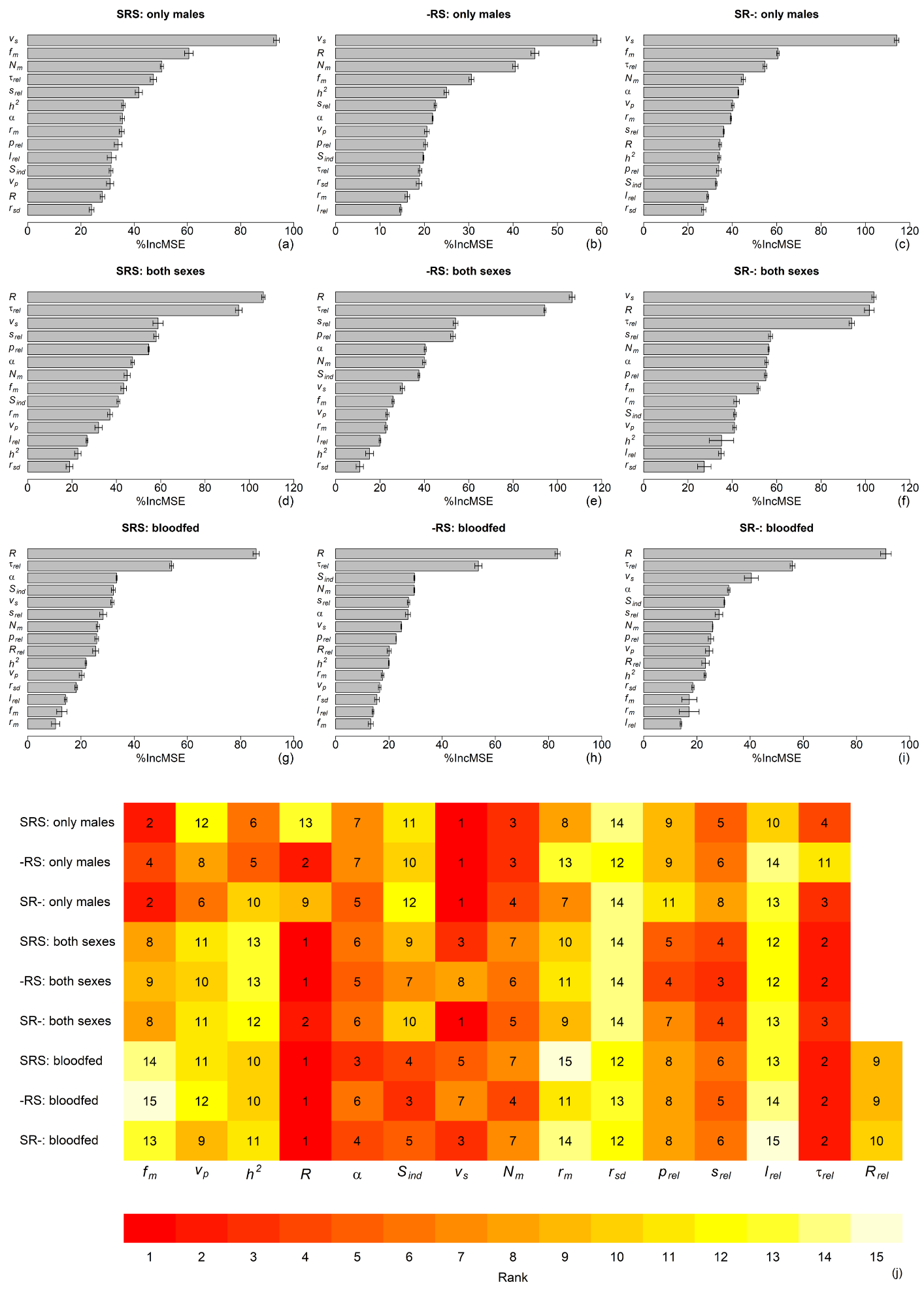
**

**Figure S4.** Parameter importance (PI) in determining the ratio of population size between the post-release and pre-release population ($N_{R}/N_{0}$) in all nine model scenarios in the quantitative polygenic model. (a)-(i) PI values of all parameters in each scenario. The error bars represent the standard errors calculated from the three replicates. Parameters are ordered decreasingly according to their PI value in each panel. (h) Heat plot of PI ranks in all nine scenarios. Ranks are shown as numbers in grids as well as colors.


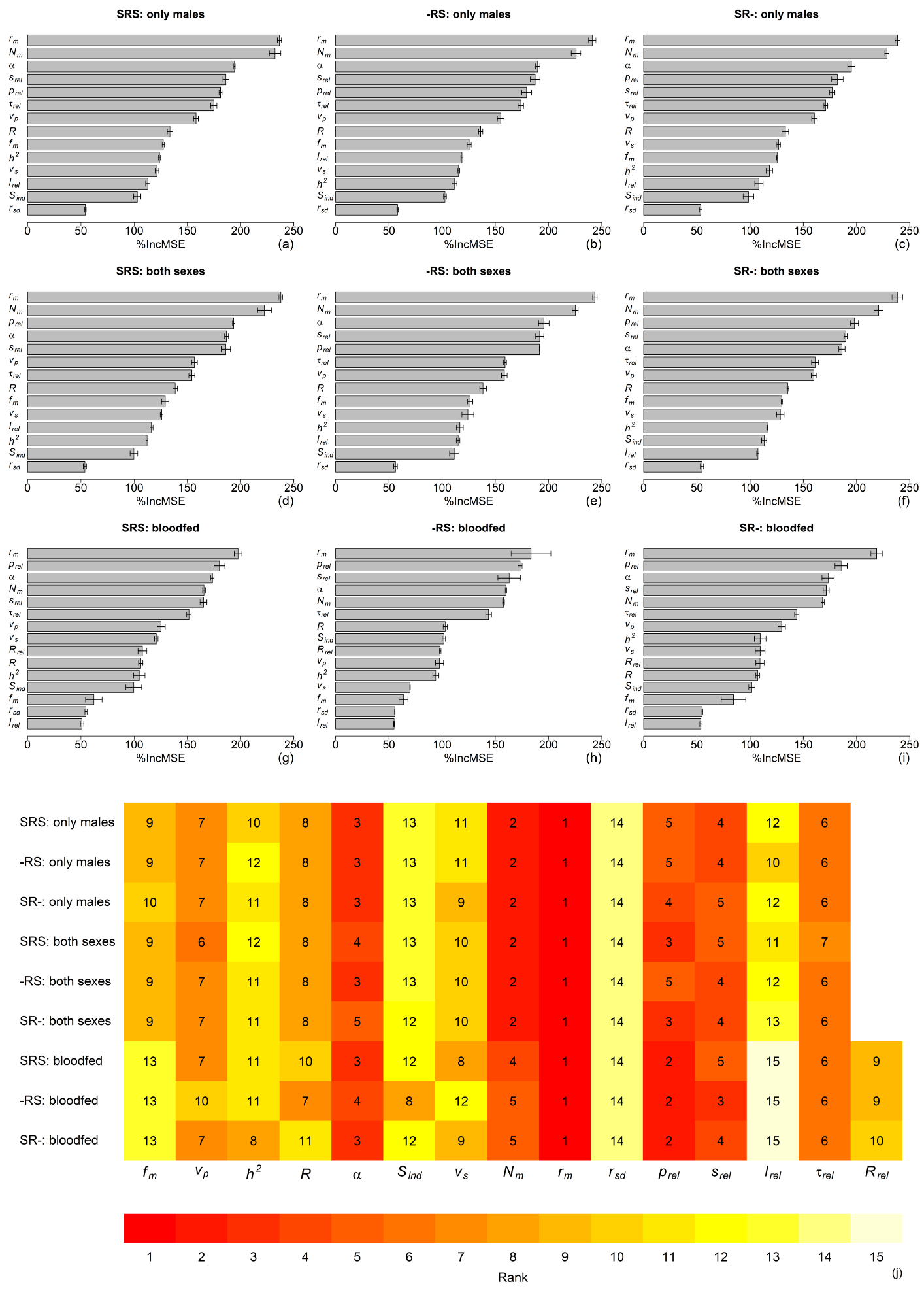


**Figure S5.** Parameter importance (PI) in determining the proportion of remaining integrated VC ($p_{VC}$) in all nine model scenarios in the quantitative polygenic model. (a)-(i) PI values of all parameters in each scenario. The error bars represent the standard errors calculated from the three replicates. Parameters are ordered decreasingly according to their PI value in each panel. (h) Heat plot of PI ranks in all nine scenarios. Ranks are shown as numbers in grids as well as colors.

**

**

**Figure S6.** Parameter importance (PI) scores calculated with increasing numbers of simulations (20,000, 40,000, 60,000, 80,000, 100,000) in the quantitative polygenic model. Each row contains results from the same model scenario (combination of release-selection order and release strategy), which is labeled on the right of each row. Each column contains results for the same efficacy metrics. Error bars represent the standard errors calculated from the three replicates. Parameters in each panel are ranked from highest PI (red lines) to lowest PI (blue lines).


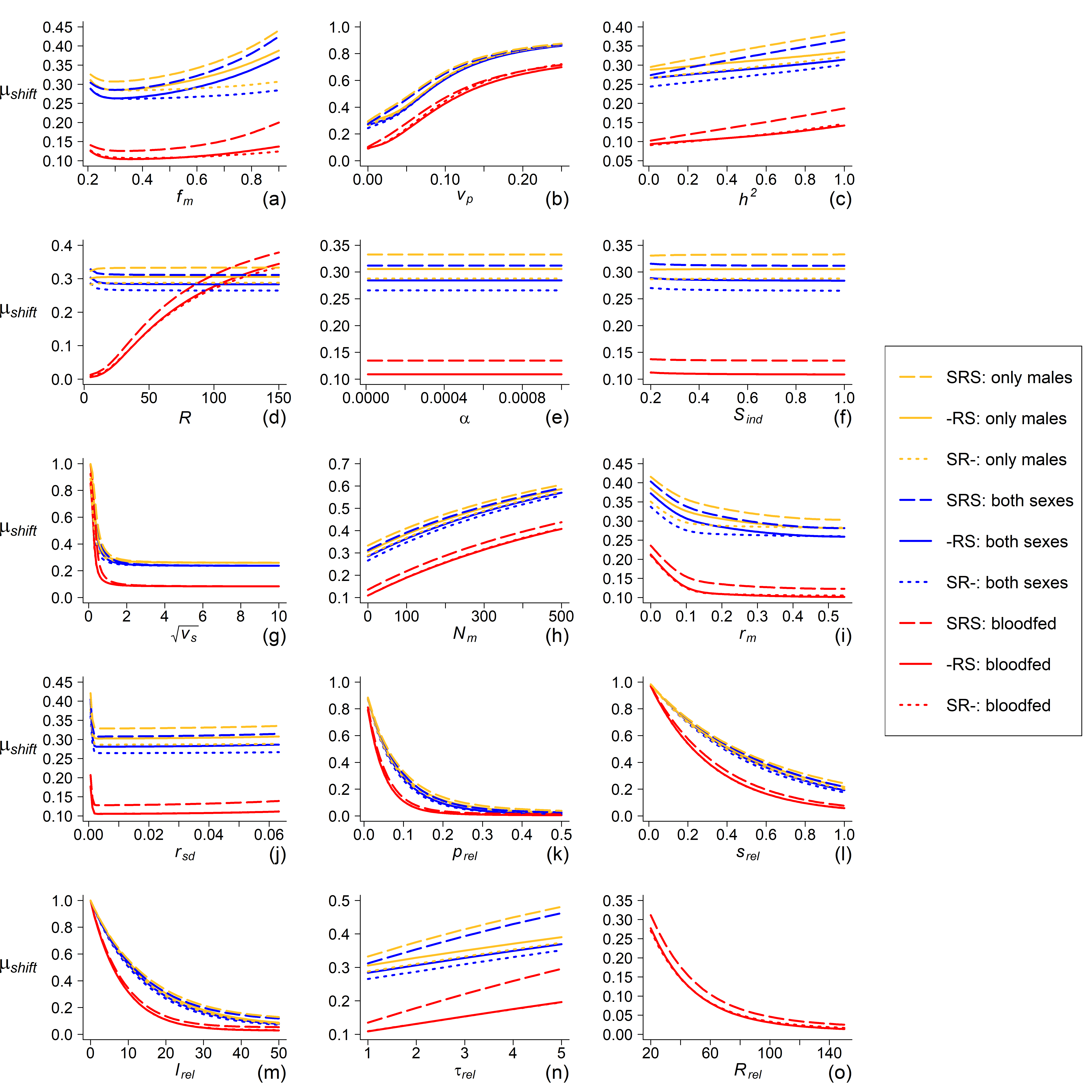
**Figure S7.** Local sensitivity analysis (LSA) of the relative mean of VC in the post-release population ($\mu_{shift}$) to each parameter given all other parameters at their default values (see Table 1 for default values and ranges). Selection variance (*v_s_*) was square-root transformed. Note the difference in the y-axis values across plots. Line types and colors are as in Figure 2.


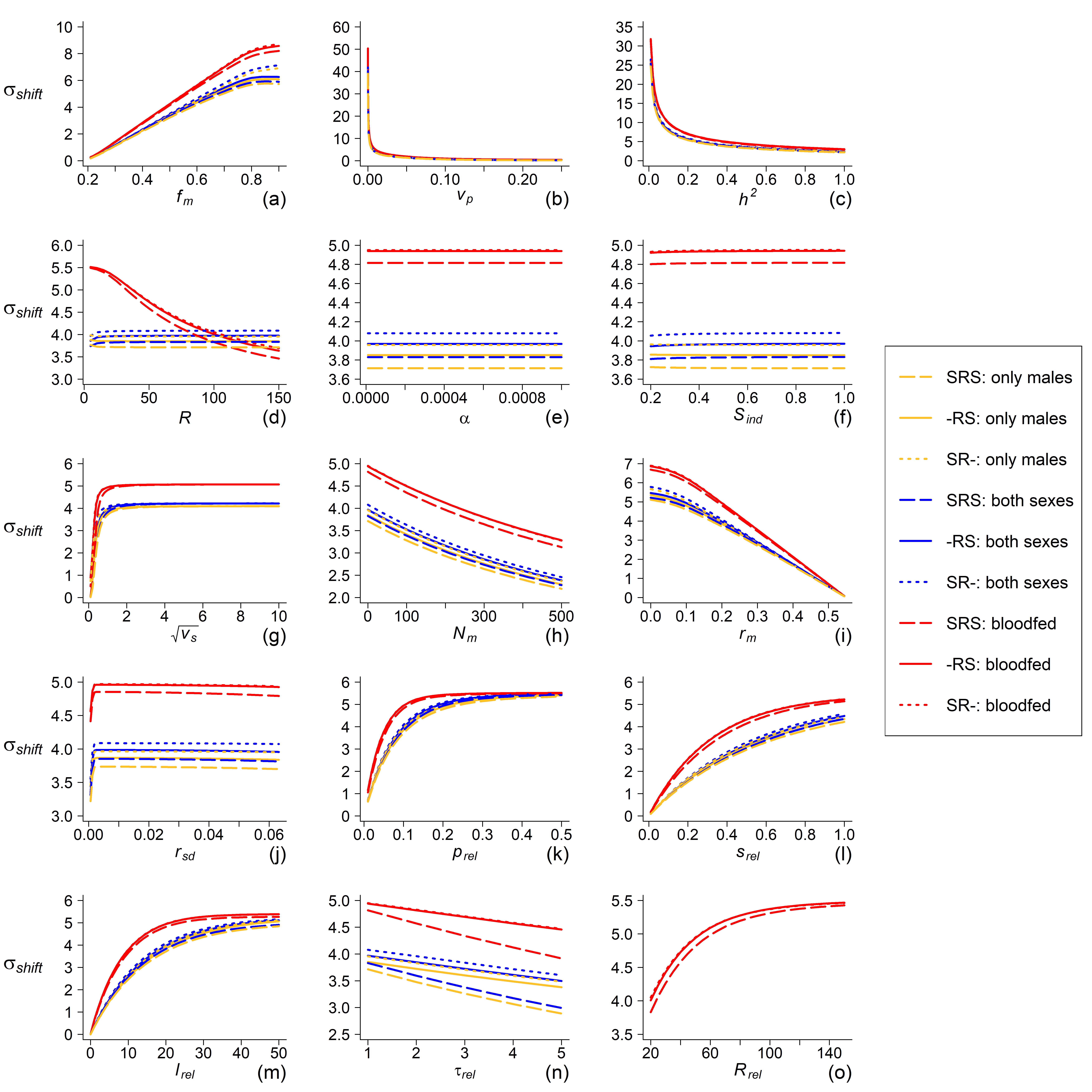


**Figure S8.** Local sensitivity analysis (LSA) of the number of SDs shifted by the VC mean ($\sigma_{shift}$) to each parameter given all other parameters at their default values (see Table 1 for default values and ranges). Selection variance (*v_s_*) was square-root transformed. Note the difference in the y-axis values across plots. Line types and colors are as in Figure 2.


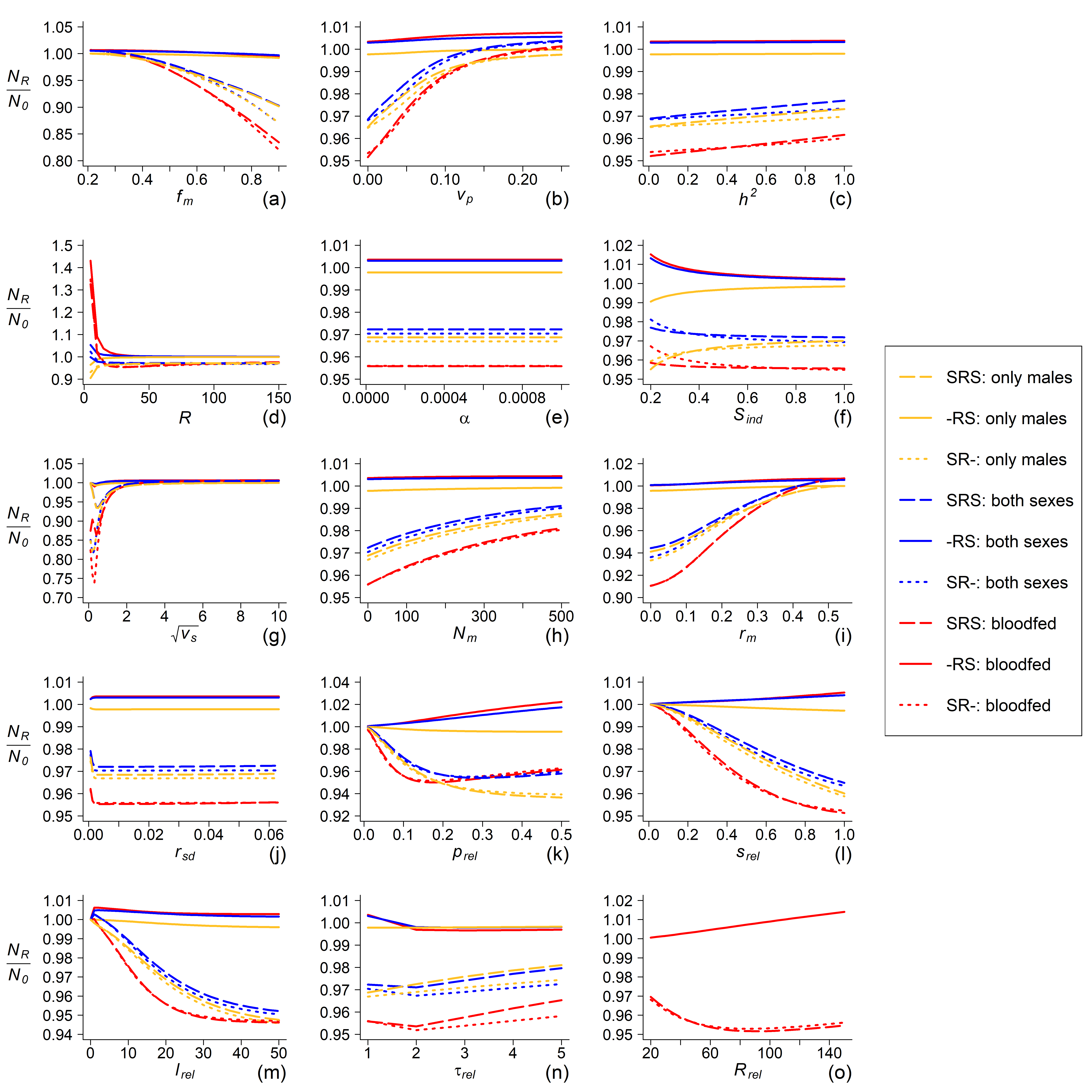


**Figure S9.** Local sensitivity analysis (LSA) of the ratio of population size between the post-release and pre-release population ($N_{R}/N_{0}$) to each parameter given all other parameters at their default values (see Table 1 for default values and ranges). Selection variance (*v_s_*) was square-root transformed. Note the difference in the y-axis values across plots. Line types and colors are as in Figure 2.

**Table S1.** Post-hoc pairwise comparisons of relative mean VC ($\mu_{shift}$) among model scenarios in the quantitative polygenic model

| $\bar{\mu_{shift}}$ | SRS  only males | -RS  only males | SR-  only males | SRS  both sexes | -RS  both sexes | SR-  both sexes | SRS  bloodfed | -RS  bloodfed | SR-  bloodfed |
| --- | --- | --- | --- | --- | --- | --- | --- | --- | --- |
| SRS  only males | 0.871^*^ | V = 4.85E9^**^ p < 0.001 | V = 4.82E9 p < 0.001 | V = 4.98E9 p < 0.001 | V = 4.97E9 p < 0.001 | V = 4.97E9 p < 0.001 | V = 4.77E9 p < 0.001 | V = 4.81E9 p < 0.001 | V = 4.80E9 p < 0.001 |
| -RS  only males |  | 0.868 | V = 1.93E9 p < 0.001 | V = 4.13E9 p < 0.001 | V = 4.98E9 p < 0.001 | V = 4.81E9 p < 0.001 | V = 4.68E9 p < 0.001 | V = 4.78E9 p < 0.001 | V = 4.75E9 p < 0.001 |
| SR-  only males |  |  | 0.867 | V = 4.16E9 p < 0.001 | V = 4.80E9 p < 0.001 | V = 4.99E9 p < 0.001 | V = 4.67E9 p < 0.001 | V = 4.76E9 p < 0.001 | V = 4.75E9 p < 0.001 |
| SRS  both sexes |  |  |  | 0.861 | V = 4.84E9 p < 0.001 | V = 4.82E9 p < 0.001 | V = 4.61E9 p < 0.001 | V = 4.68E9 p < 0.001 | V = 4.66E9 p < 0.001 |
| -RS  both sexes |  |  |  |  | 0.858 | V = 1.99E9 p < 0.001 | V = 4.50E9 p < 0.001 | V = 4.62E9 p < 0.001 | V = 4.59E9 p < 0.001 |
| SR-  both sexes |  |  |  |  |  | 0.858 | V = 4.49E9 p < 0.001 | V = 4.61E9 p < 0.001 | V = 4.59E9 p < 0.001 |
| SRS  bloodfed |  |  |  |  |  |  | 0.796 | V = 4.82E9 p < 0.001 | V = 4.36E9 p < 0.001 |
| -RS  bloodfed |  |  |  |  |  |  |  | 0.791 | V = 5.81E8 p < 0.001 |
| SR-  bloodfed |  |  |  |  |  |  |  |  | 0.793 |

^*^ Values in the diagonal are the mean $\mu_{shift}$ of scenarios calculated from all GSA simulations.

^**^ Wilcoxon signed rank tests with Bonferroni correction.

**Table S2.** Post-hoc pairwise comparisons of number of SDs shifted ($\sigma_{shift}$) among model scenarios in the quantitative polygenic model

| $\bar{\sigma_{shift}}$ | SRS  only males | -RS  only males | SR-  only males | SRS  both sexes | -RS  both sexes | SR-  both sexes | SRS  bloodfed | -RS  bloodfed | SR-  bloodfed |
| --- | --- | --- | --- | --- | --- | --- | --- | --- | --- |
| SRS  only males | 0.681^*^ | V = 5.99E8^**^ p < 0.001 | V = 5.87E8 p < 0.001 | V = 1.88E6 p < 0.001 | V = 2.05E7 p < 0.001 | V = 1.75E7 p < 0.001 | V = 2.99E8 p < 0.001 | V = 2.47E8 p < 0.001 | V = 2.51E8 p < 0.001 |
| -RS  only males |  | 0.690 | V = 2.37E9 p < 0.001 | V = 8.40E8 p < 0.001 | V = 2.07E6 p < 0.001 | V = 1.28E8 p < 0.001 | V = 3.86E8 p < 0.001 | V = 2.82E8 p < 0.001 | V = 2.95E8 p < 0.001 |
| SR-  only males |  |  | 0.699 | V = 8.79E8 p < 0.001 | V = 2.55E8 p < 0.001 | V = 2.90E6 p < 0.001 | V = 4.16E8 p < 0.001 | V = 3.13E8 p < 0.001 | V = 3.21E8 p < 0.001 |
| SRS  both sexes |  |  |  | 0.726 | V = 6.08E8 p < 0.001 | V = 5.88E8 p < 0.001 | V = 4.64E8 p < 0.001 | V = 3.96E8 p < 0.001 | V = 4.04E8 p < 0.001 |
| -RS  both sexes |  |  |  |  | 0.736 | V = 2.33E9 p < 0.001 | V = 5.73E8 p < 0.001 | V = 4.45E8 p < 0.001 | V = 4.64E8 p < 0.001 |
| SR-  both sexes |  |  |  |  |  | 0.746 | V = 6.04E8 p < 0.001 | V = 4.80E8 p < 0.001 | V = 4.95E8 p < 0.001 |
| SRS  bloodfed |  |  |  |  |  |  | 0.989 | V = 6.03E8 p < 0.001 | V = 1.08E9 p < 0.001 |
| -RS  bloodfed |  |  |  |  |  |  |  | 1.008 | V = 4.20E9 p < 0.001 |
| SR-  bloodfed |  |  |  |  |  |  |  |  | 1.006 |

^*^ Values in the diagonal are the mean $\sigma_{shift}$ of scenarios calculated from all GSA simulations.

^**^ Wilcoxon signed rank tests with Bonferroni correction.

**Table S3.** Post-hoc pairwise comparisons of population size ratio ($N_{R}/N_{0}$) among model scenarios in the quantitative polygenic model

| $\bar{\frac{N_{R}}{N_{0}}}$ | SRS  only males | -RS  only males | SR-  only males | SRS  both sexes | -RS  both sexes | SR-  both sexes | SRS  bloodfed | -RS  bloodfed | SR-  bloodfed |
| --- | --- | --- | --- | --- | --- | --- | --- | --- | --- |
| SRS  only males | 0.999^*^ | V = 1.71E9^**^ p < 0.001 | V = 1.79E9 p < 0.001 | V = 9.71E8 p < 0.001 | V = 7.48E8 p < 0.001 | V = 1.02E9  p < 0.001 | V = 1.58E9 p < 0.001 | V = 7.73E8 p < 0.001 | V = 1.56E9 p < 0.001 |
| -RS  only males |  | 1.000 | V = 3.27E9 p < 0.001 | V = 1.71E9 p < 0.001 | V = 4.29E8 p < 0.001 | V = 1.72E9 p < 0.001 | V = 1.90E9 p < 0.001 | V = 8.62E8 p < 0.001 | V = 1.88E9 p < 0.001 |
| SR-  only males |  |  | 0.999 | V = 1.03E9 p < 0.001 | V = 7.77E8 p < 0.001 | V = 1.01E9 p < 0.001 | V = 1.55E9 p < 0.001 | V = 7.79E8 p < 0.001 | V = 1.54E9 p < 0.001 |
| SRS  both sexes |  |  |  | 1.001 | V = 1.72E9 p < 0.001 | V = 2.17E9 p < 0.001 | V = 2.55E9 p < 0.001 | V = 1.48E9 p < 0.001 | V = 2.52E9 **p = 1** |
| -RS  both sexes |  |  |  |  | 1.002 | V = 3.33E9 p < 0.001 | V = 2.67E9 p < 0.001 | V = 2.26E9 p < 0.001 | V = 2.65E9 p < 0.001 |
| SR-  both sexes |  |  |  |  |  | 1.001 | V = 2.51E9 **p = 1** | V = 1.47E9 p < 0.001 | V = 2.50E9 **p = 1** |
| SRS  bloodfed |  |  |  |  |  |  | 1.005 | V = 1.69E9 p < 0.001 | V = 2.17E9 p < 0.001 |
| -RS  bloodfed |  |  |  |  |  |  |  | 1.006 | V = 3.38E9 p < 0.001 |
| SR-  bloodfed |  |  |  |  |  |  |  |  | 1.005 |

^*^ Values in the diagonal are the mean $N_{R}/N_{0}$ of scenarios calculated from all GSA simulations.

^**^ Wilcoxon signed rank tests with Bonferroni correction.

**Table S4.** Post-hoc pairwise comparisons of proportion of integrated VC ($p_{VC}$) among model scenarios in the quantitative polygenic model

| $\bar{p_{VC}}$ | SRS  only males | -RS  only males | SR-  only males | SRS  both sexes | -RS  both sexes | SR-  both sexes | SRS  bloodfed | -RS  bloodfed | SR-  bloodfed |
| --- | --- | --- | --- | --- | --- | --- | --- | --- | --- |
| SRS  only males | 0.938^*^ | V = 4.36E9^**^ p < 0.001 | V = 4.85E9 p < 0.001 | V = 4.20E9 p < 0.001 | V = 4.36E9 p < 0.001 | V = 4.39E9 p < 0.001 | V = 4.64E9 p < 0.001 | V = 4.69E9 p < 0.001 | V = 4.69E9 p < 0.001 |
| -RS  only males |  | 0.936 | V = 2.76E9 p < 0.001 | V = 3.50E9 p < 0.001 | V = 4.22E9 p < 0.001 | V = 4.12E9 p < 0.001 | V = 4.55E9 p < 0.001 | V = 4.65E9 p < 0.001 | V = 4.64E9 p < 0.001 |
| SR-  only males |  |  | 0.936 | V = 3.41E9 p < 0.001 | V = 3.97E9 p < 0.001 | V = 4.24E9 p < 0.001 | V = 4.52E9 p < 0.001 | V = 4.61E9 p < 0.001 | V = 4.62E9 p < 0.001 |
| SRS  both sexes |  |  |  | 0.935 | V = 4.35E9 p < 0.001 | V = 4.86E9 p < 0.001 | V = 4.54E9 p < 0.001 | V = 4.60E9 p < 0.001 | V = 4.60E9 p < 0.001 |
| -RS  both sexes |  |  |  |  | 0.934 | V = 2.82E9 p < 0.001 | V = 4.44E9 p < 0.001 | V = 4.56E9 p < 0.001 | V = 4.54E9 p < 0.001 |
| SR-  both sexes |  |  |  |  |  | 0.933 | V = 4.40E9 p < 0.001 | V = 4.50E9 p < 0.001 | V = 4.51E9 p < 0.001 |
| SRS  bloodfed |  |  |  |  |  |  | 0.906 | V = 4.37E9 p < 0.001 | V = 4.44E9 p < 0.001 |
| -RS  bloodfed |  |  |  |  |  |  |  | 0.904 | V = 1.94E9 p < 0.001 |
| SR-  bloodfed |  |  |  |  |  |  |  |  | 0.904 |

^*^ Values in the diagonal are the mean $p_{VC}$ of scenarios calculated from all GSA simulations.

^**^ Wilcoxon signed rank tests with Bonferroni correction.
