# Supplementary material for: Quantifying the efficacy of genetic shifting in control of mosquito‐borne diseases
Source: Evol Appl. 2019 Jun 14;12(8):1552–68. doi: 10.1111/eva.12802 (PMC6708429; doi:10.1111/eva.12802)
Supplement: Supplementary file 2 [file EVA-12-1552-s002.docx]

**Appendix 2: Mathematical details and results of the one-locus Mendelian model**

*Mathematical details*

In the one-locus Mendelian model, an individual’s VC depends on a single gene with two alleles (susceptible allele *S* and resistant allele *R*) and three possible genotypes: *SS* (*g*=1), *SR* (*g*=*d*) and *RR* (*g*=0), where *d* is the degree of dominance of the susceptible allele (*S*). The population density distribution *n_i,t_(g)* of population *i* at generation *t* therefore comprises three discrete values: *n_i,t_(1), n_i,t_(d), n_i,t_(0)*. The total population size *N_i,_*_t_ = ∑ *n_i,t_(g)*. The allele frequencies *q_i,t_(S)* and *q_,t_(R)* are:

$$\begin{aligned} \left\{ \begin{aligned} q_{i,t}\left( S \right)=\frac{n_{i,t}\left( 1 \right)+0.5*n_{i,t}\left( d \right)}{N_{i,t}} \\ q_{i,t}\left( R \right)=\frac{n_{i,t}\left( 0 \right)+0.5*n_{i,t}\left( d \right)}{N_{i,t}} \end{aligned} \right.\#(S8) \end{aligned}$$

Similar to the quantitative polygenic model, we separate males (*M*) and females (*F*) in the reproduction and release steps. The allele frequencies of *S* and *R* in the parental population represent the gemate frequencies. We assume random mating such that allele frequencies are exactly their encounter likelihood to determine the offspring genotype distribution. Multiplying this offspring genotype distribution by the number of offspring per female *R* and the population size of females $N_{tgt, F,t}$ in the parental generation gives rise to the density distribution in the offspring generation for male-only and both-sex release strategies:

$$\begin{aligned} \left\{ \begin{aligned} &n_{tgt,t}^{*}\left( 1 \right)=R N_{tgt, F,t} q_{i, F,t}\left( S \right)q_{i, M,t}\left( S \right) \\ &n_{tgt,t}^{*}\left( d \right)=R N_{tgt, F,t} \left( q_{i,F,t}\left( S \right)q_{i,M,t}\left( R \right)+q_{i,F,t}\left( R \right)q_{i,M,t}\left( S \right) \right) \\ &n_{tgt,t}^{*}\left( 0 \right)=R N_{tgt, F,t} q_{i, F,t}\left( R \right)q_{i, M,t}\left( R \right) \end{aligned} \right..\#(S9-1) \end{aligned}$$

When releasing blood-fed females, we separately account for the released females’ reproductive contribution *R_rel_* as in the quantitative polygenic model:

$$\begin{aligned} \left\{ \begin{aligned} &n_{tgt,t}^{*}\left( 1 \right)=RN_{tgt, F,t}q_{i, F,t}\left( S \right)q_{i, M,t}\left( S \right)+R_{rel}N_{rel,F}\psi_{rel}\left( 1 \right) \\ &n_{tgt,t}^{*}\left( d \right)=RN_{tgt, F,t}\left( q_{i,F,t}\left( S \right)q_{i,M,t}\left( R \right)+q_{i,F,t}\left( R \right)q_{i,M,t}\left( S \right) \right)+R_{rel}N_{rel,F}\psi_{rel}\left( d \right) \\ &n_{tgt,t}^{*}\left( 0 \right)=RN_{tgt, F,t}q_{i, F,t}\left( R \right)q_{i, M,t}\left( R \right)+R_{rel}N_{rel,F}\psi_{rel}\left( 0 \right) \end{aligned} \right.\#(S9-2) \end{aligned}$$

We model density-dependent survival in the larval stage and density-independent survival in the pupae stage the same as in the quantitative polygenic model (Equations 2 and 3). The stabilizing selection in the adult stage is modeled as a frequency-dependent selection with selection strength $s_{fd}$:

$$\begin{aligned} n_{tgt, t}^{++}\left( g \right)=\left( 0.5-\left( \frac{n_{tgt,t}\left( g \right)}{N_{tgt,t}}-f_{opt}\left( g \right) \right)s_{fd} \right)n_{tgt, t}^{+}\left( g \right), \#\left( S10 \right) \end{aligned}$$

where $f_{opt}(g)$ is the optimal genotype frequency determined by the optimal allele frequency *A_w_* following Hardy-Weinberger equilibrium: $f_{opt}\left( 1 \right)=A_{w}^{2}$, $f_{opt}\left( d \right)=2A_{w}(1-A_{w})$, and $f_{opt}\left( 0 \right)={(1-A_{w})}^{2}$.

Release and migration in the adult stage follows Equations 5 (when releasing both sexes without feeding) or Equation S1 (when releasing only males) or Equation S3 (when releasing blood-fed females with males), as in the quantitative polygenic model. The release population has a lower allele frequency of the susceptible allele (*A_r_*) than the wild population. The genotype frequency $f_{rel}\left( g \right)$ follows the Hardy-Weinberger equilibrium: $f_{rel}\left( 1 \right)=A_{r}^{2}$, $f_{rel}\left( d \right)=2A_{r}(1-A_{r})$, and $f_{rel}\left( 0 \right)={(1-A_{r})}^{2}$. The immigrant population has the same genotype frequency distribution as the pre-release target population.

We numerically implement the model by iterating through the life cycle of mosquitoes, rearranging the orders of equations according to different orders of release and selection (Fig. 1). We use the same values and ranges for parameters shared with the quantitative polygenic model (Table S5). *A_w_* and *A_r_* has the same values and ranges as *f_m_* and *r_m_* (Table 1). We summarize the release efficacy with the same four metrics ($\mu_{shift}$, $\sigma_{shift}$, $\frac{N_{R}}{N_{0}}$, and $p_{VC}$, Equation S4-S7).

**Table S5.** Descriptions and values of model parameters in the one-locus Mendelian model

| Parameter | Description | Default | Range |
| --- | --- | --- | --- |
| *A_w_* | Optimal frequency of the susceptible allele (S) in the wild | 0.55 | 0.1 – 0.9^+^ |
| *d* | Dominance of the susceptible alleles (S) | 0.5 | 0 – 1 |
| *R* | Mean number of offspring per female | 40 | 5 – 150 |
| *α* | Beverton-Holt density-dependent saturation constant | 10^-4^ | 10^-5^ – 10^-3^ |
| *S_ind_* | Density-independent survival probability | 0.7 | 0.2 – 1 |
| *s_fd_* | Strength of frequency-dependent selection | 0.1^#^ | 0 – 0.5^#^ |
| *N_m_* | Number of immigrants from external population | 0 | 0 – 500 |
| *A_r_* | Frequency of susceptible allele (S) in the release population | 0.2 | (0 – 1) *A_w_* |
| *p_rel,t_* | Relative size of the releasing population at generation *t* | 0.1*^++^* | 0.01 – 0.5*^++^* |
| *s_rel_* | Mean survival probability of releasing individuals | 0.75 | 0.01 – 1 |
| *l_rel_* | Number of releases | 20 | 1 – 50 |
| *τ_rel_* | Release frequency: number of generations between releases | 1 | 1 – 5 |
| *R_rel_* | Reproductive output of blood-fed releasing female | 50 | 20 – 150 |

^+^ In the local sensitivity analysis, *A_w_* ranges from 0.2 – 0.9, as the susceptible allele frequency should be lower in the release population than in the wild population.

^++^ *p_rel,t_* = 0 when no release occurs at generation *t*.

^#^ Little empirical data exist so we determine the value and the range based on experience.

*Results*


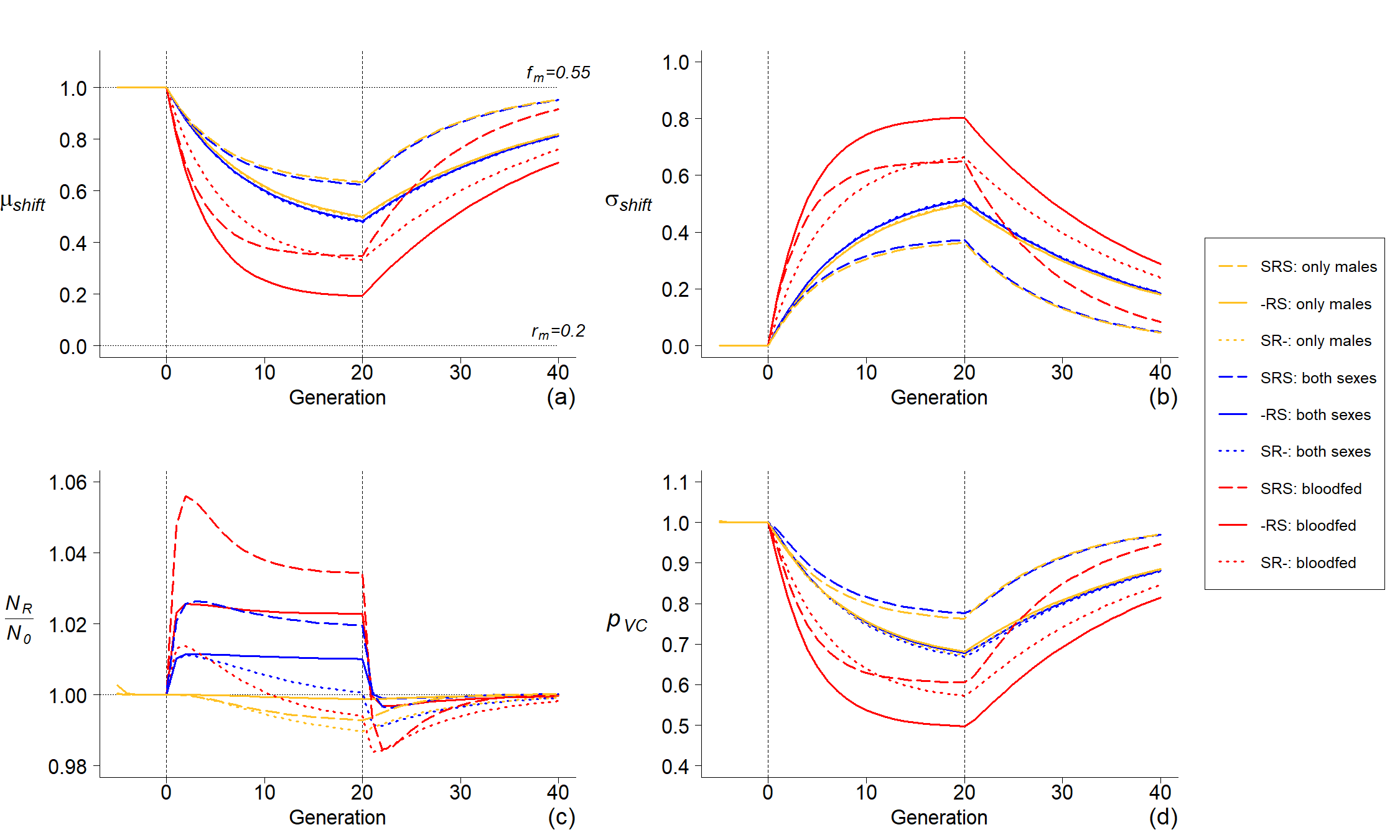


**Figure S10.** Change of VC during 20 generations of releases and 20 generations of recovery in the one-locus Mendelian model. The model followed the changes of (a) relative mean of VC in the post-release population ($\mu_{shift}$)**,** (b) number of SDs shifted by the VC mean ($\sigma_{shift}$), (c) ratio of population size between the post-release and pre-release population ($N_{R}/N_{0}$), and (d) the proportion of remaining integrated VC ($p_{VC}$) in the target population. Line types and colors are as in Figure 2. The first and second dashed vertical lines indicate the start and the end of the releases. Five generations before the release started are also shown to demonstrate the equilibrium state of the pre-release population. The horizontal lines in (a) indicate the selection optimum (*f_m_*) and the mean VC of the release population (*r_m_*). We model all scenarios using the default parameter values in Table S5. Note that the vertical axes do not start from 0 in panels (c) and (d).


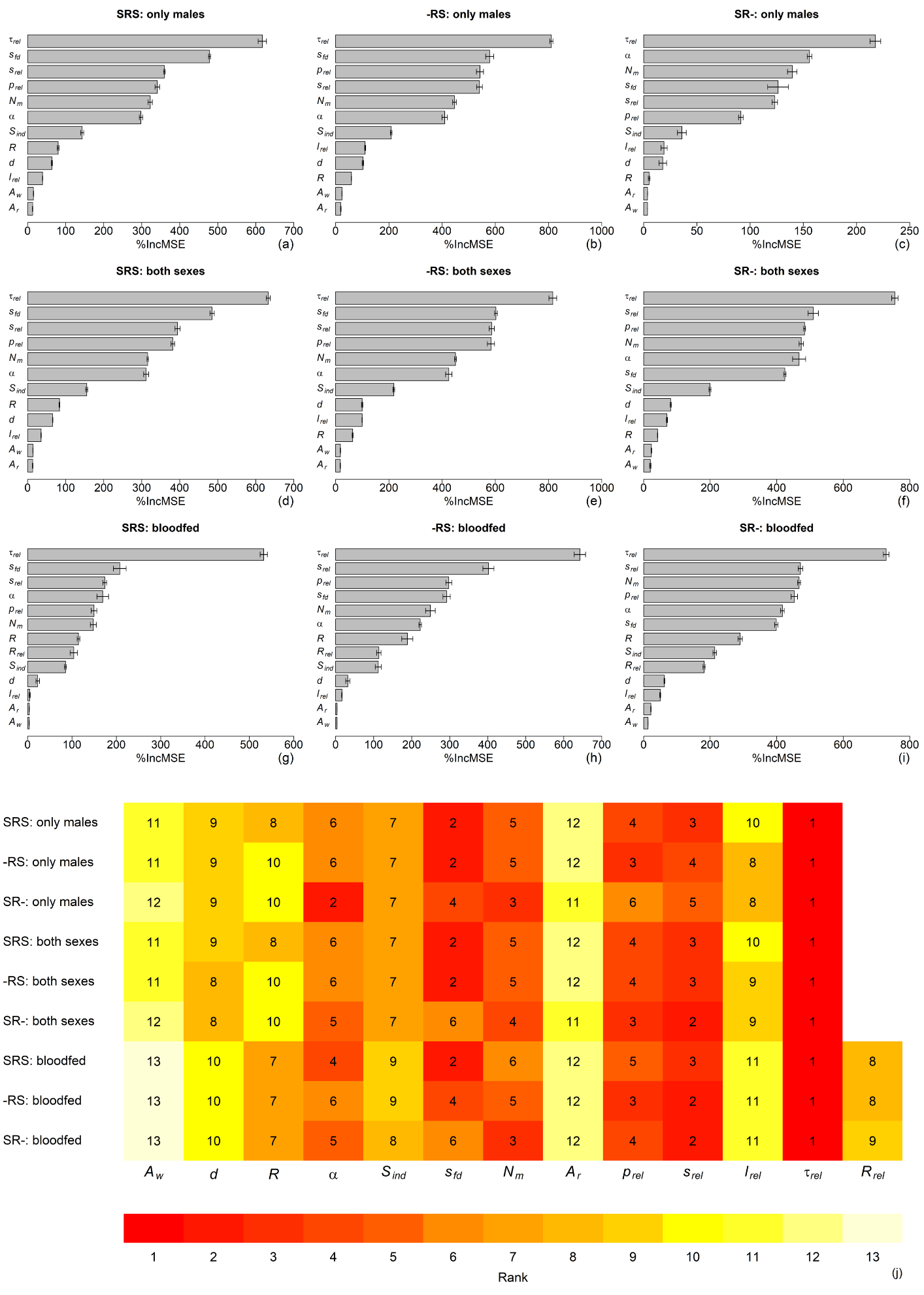


**Figure S11.** Parameter importance (PI) in determining the relative mean of VC in the post-release population ($\mu_{shift}$) in all nine model scenarios in the one-locus Mendelian model. (a)-(i) PI values of all parameters in each scenario. The error bars represent the standard errors calculated from the three replicates. Parameters are ordered decreasingly according to their PI value in each panel. (h) Heat plot of PI ranks in all nine scenarios. Ranks are shown as numbers in grids as well as colors.


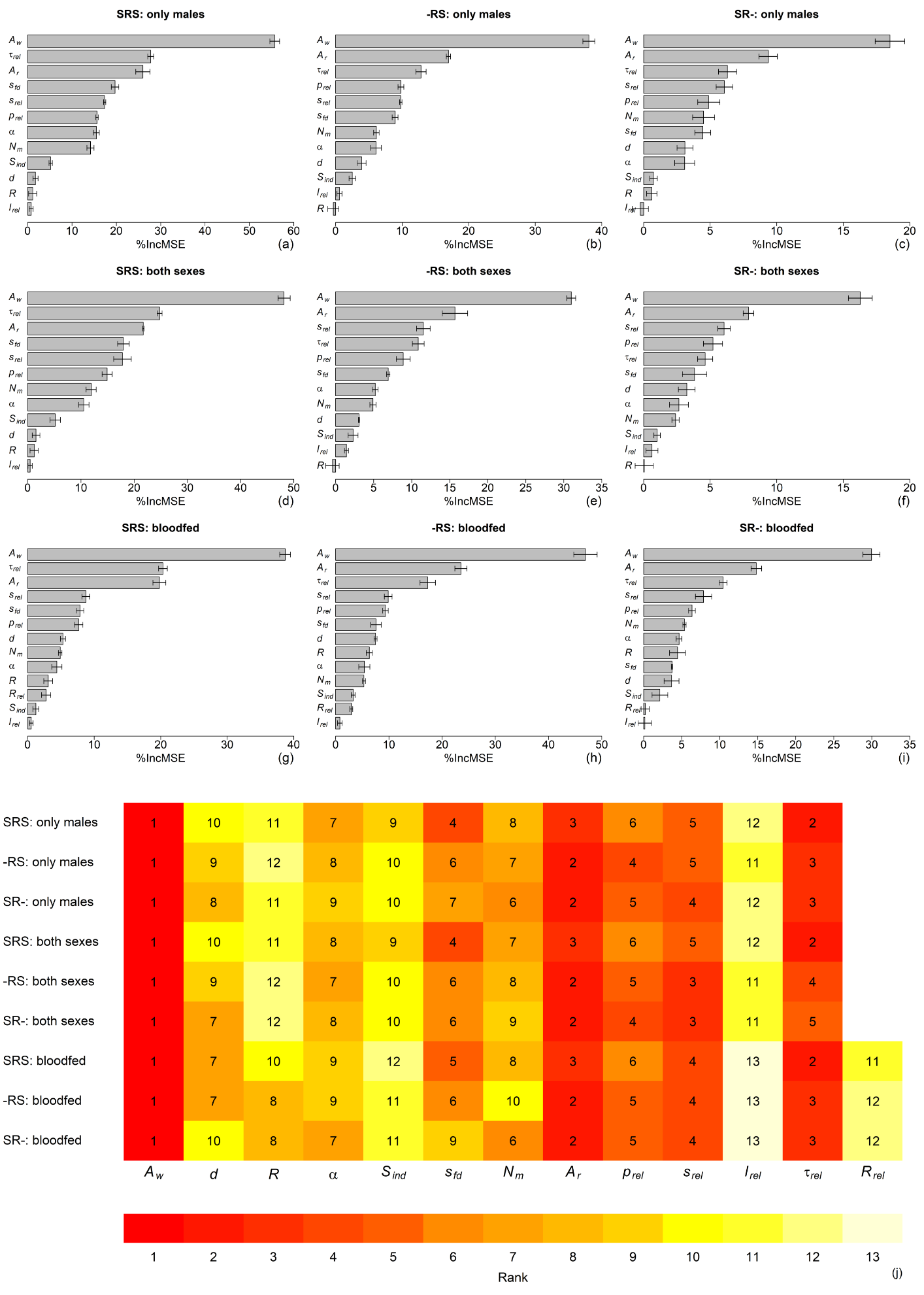


**Figure S12.** Parameter importance (PI) in determining the number of SDs shifted by the VC mean ($\sigma_{shift}$) in all nine model scenarios in the one-locus Mendelian model. (a)-(i) PI values of all parameters in each scenario. The error bars represent the standard errors calculated from the three replicates. Parameters are ordered decreasingly according to their PI value in each panel. (h) Heat plot of PI ranks in all nine scenarios. Ranks are shown as numbers in grids as well as colors.

**
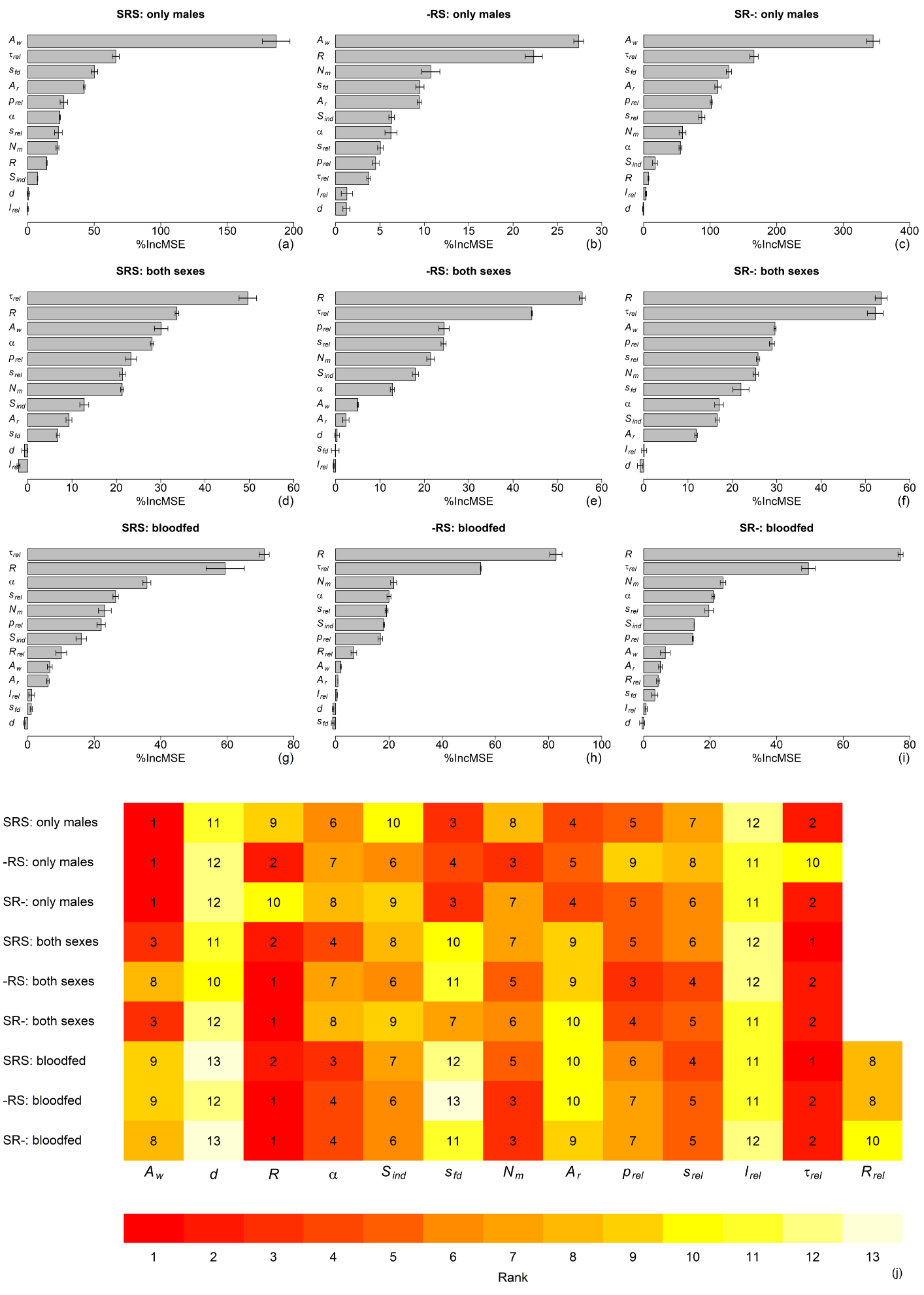
**

**Figure S13.** Parameter importance (PI) in determining the ratio of population size between the post-release and pre-release population ($N_{R}/N_{0}$) in all nine model scenarios in the one-locus Mendelian model. (a)-(i) PI values of all parameters in each scenario. The error bars represent the standard errors calculated from the three replicates. Parameters are ordered decreasingly according to their PI value in each panel. (h) Heat plot of PI ranks in all nine scenarios. Ranks are shown as numbers in grids as well as colors.

**
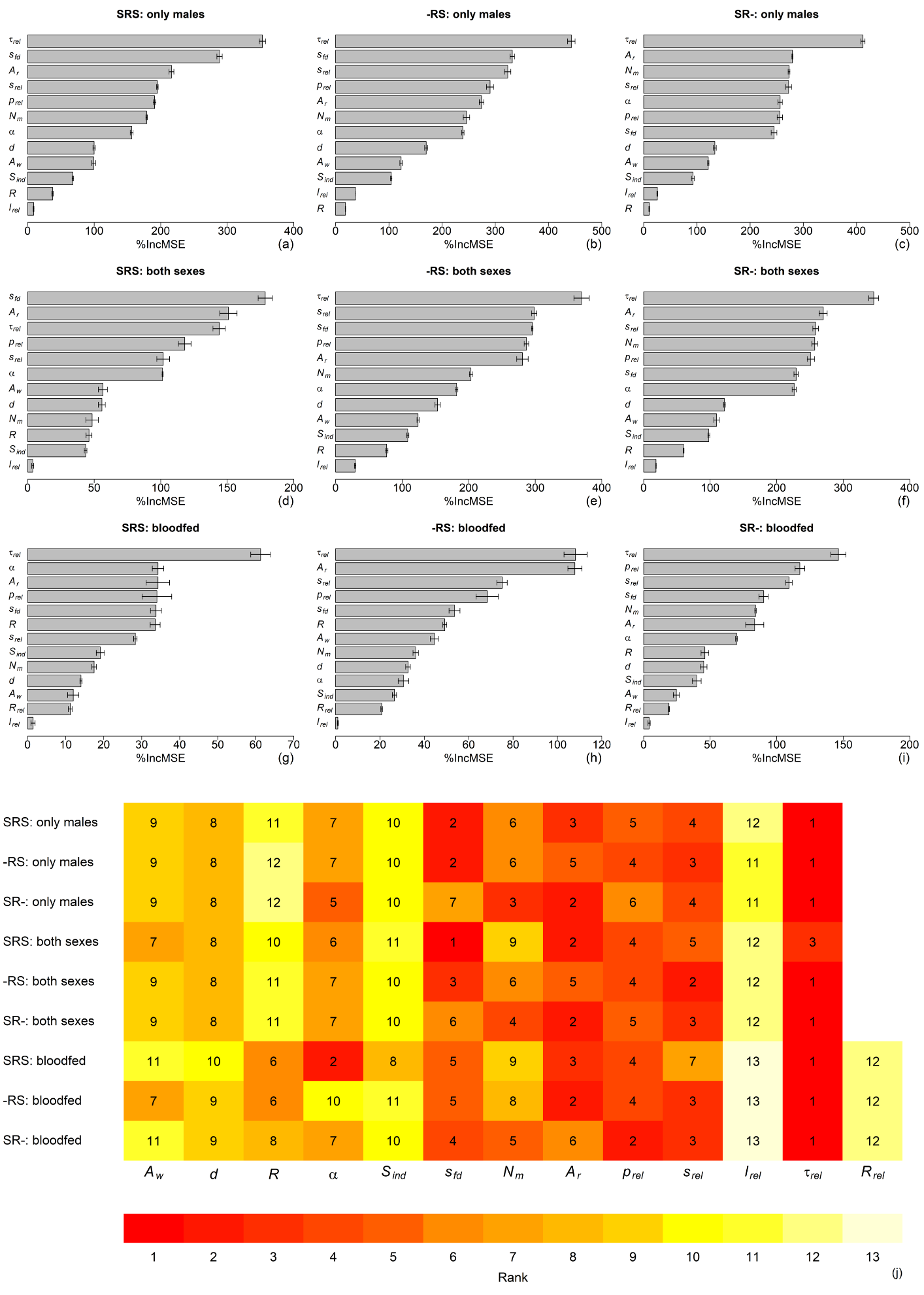
**

**Figure S14.** Parameter importance (PI) in determining the proportion of remaining integrated VC ($p_{VC}$) in all nine model scenarios in the one-locus Mendelian model. (a)-(i) PI values of all parameters in each scenario. The error bars represent the standard errors calculated from the three replicates. Parameters are ordered decreasingly according to their PI value in each panel. (h) Heat plot of PI ranks in all nine scenarios. Ranks are shown as numbers in grids as well as colors.

**
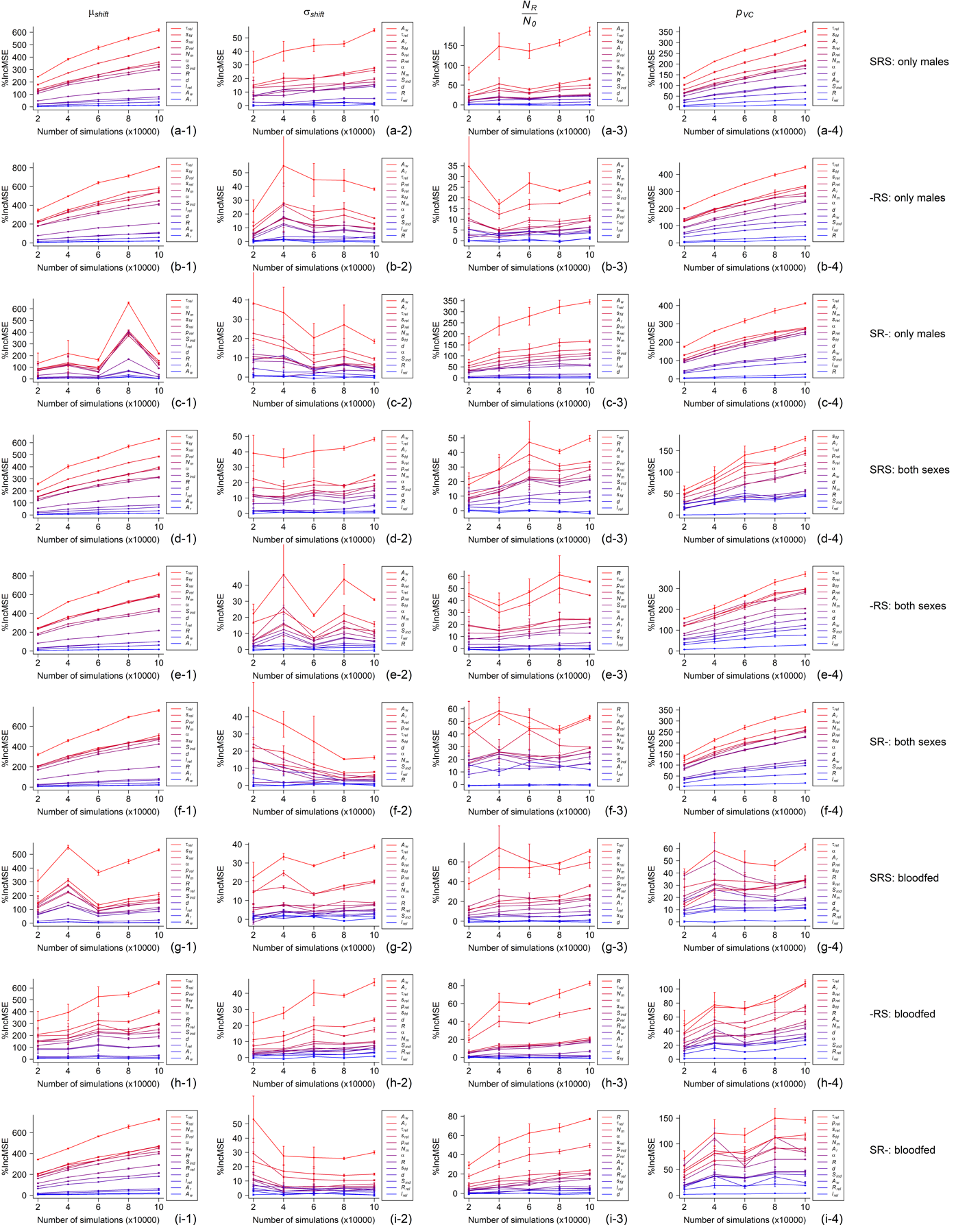
**

**Figure S15.** Parameter importance (PI) scores calculated with increasing numbers of simulations (20,000, 40,000, 60,000, 80,000, 100,000) in the one-locus Mendelian model. Each row contains results from the same model scenario (combination of release-selection order and release strategy), which is labeled on the right of each row. Each column contains results for the same efficacy metrics. Error bars represent the standard errors calculated from the three replicates. Parameters in each panel are ranked from highest PI (red lines) to lowest PI (blue lines).


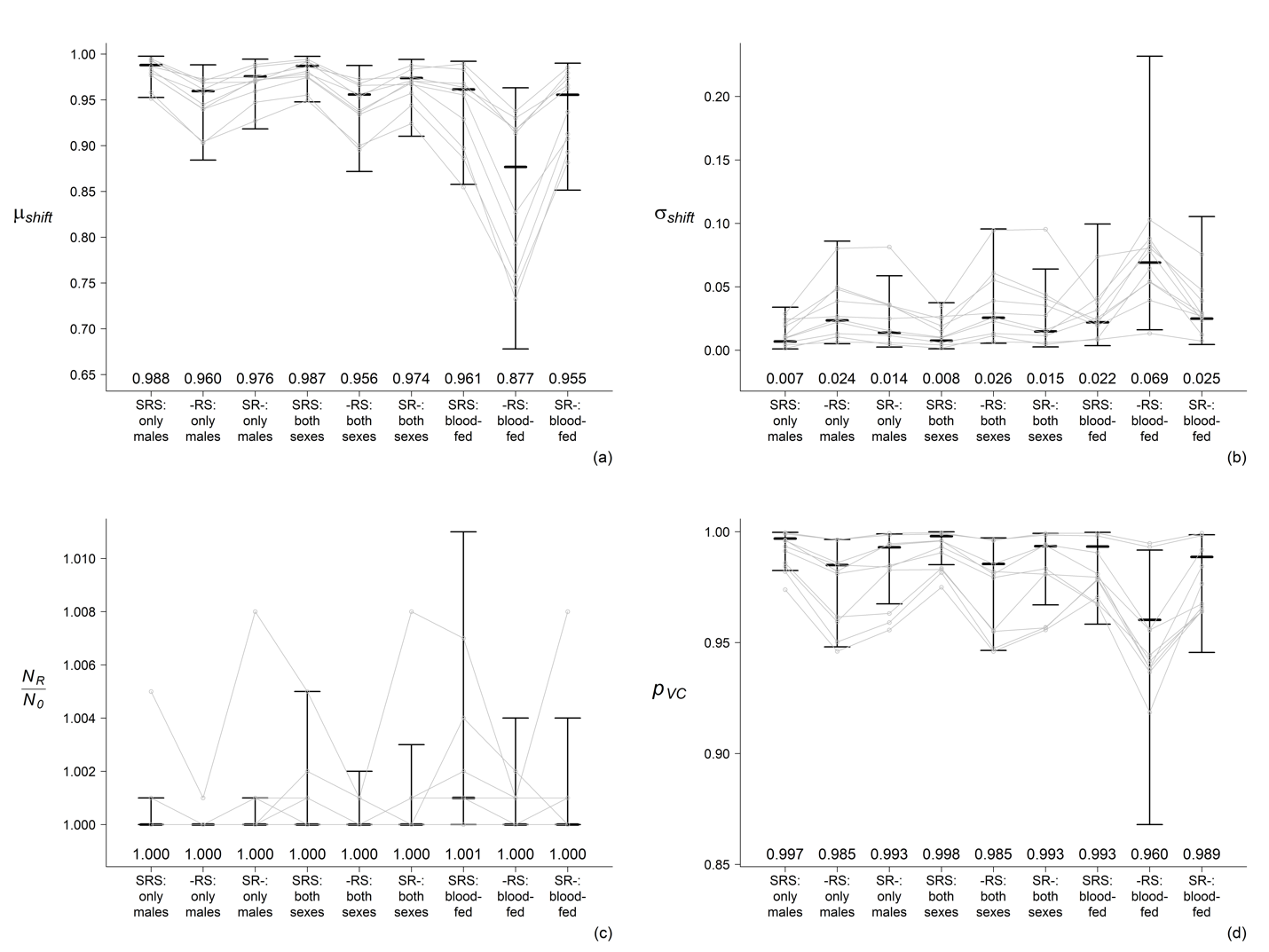


**Figure S16.** Median and interquartile range of each efficacy metrics for all nine model scenarios, calculated at *t* = $l_{rel}\times\tau_{rel}$ from the 100,000 GSA simulations in the one-locus Mendelian model: (a) relative mean of VC in the post-release population ($\mu_{shift}$)**,** (b) number of SDs shifted by the VC mean ($\sigma_{shift}$), (c) ratio of population size between the post-release and pre-release population ($N_{R}/N_{0}$), and (d) the proportion of remaining integrated VC ($p_{VC}$). The three bars in each scenario represent the 25% quantile, the median and the 75% quantile, respectively. The gray lines represent 10 randomly selected simulations. The numbers at the bottom of each figure show the median values of the 100,000 simulations.


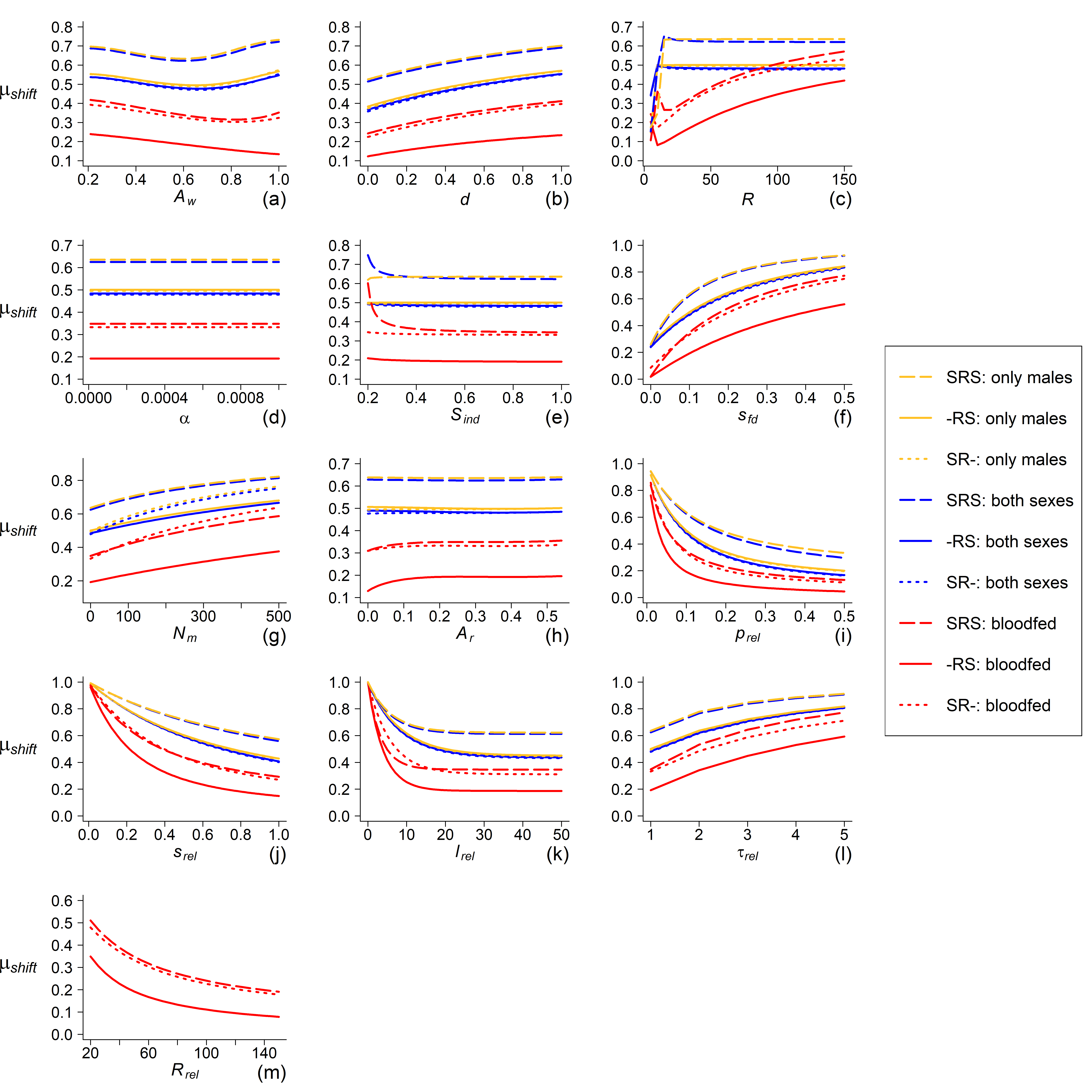


**Figure S17.** Local sensitivity analysis (LSA) of the relative mean of VC in the post-release population ($\mu_{shift}$) to each parameter given all other parameters at their default values (see Table S5 for default values and ranges) in the one-locus Mendelian model. Note the difference in the y-axis values across plots. Line types and colors are as in Figure 2.


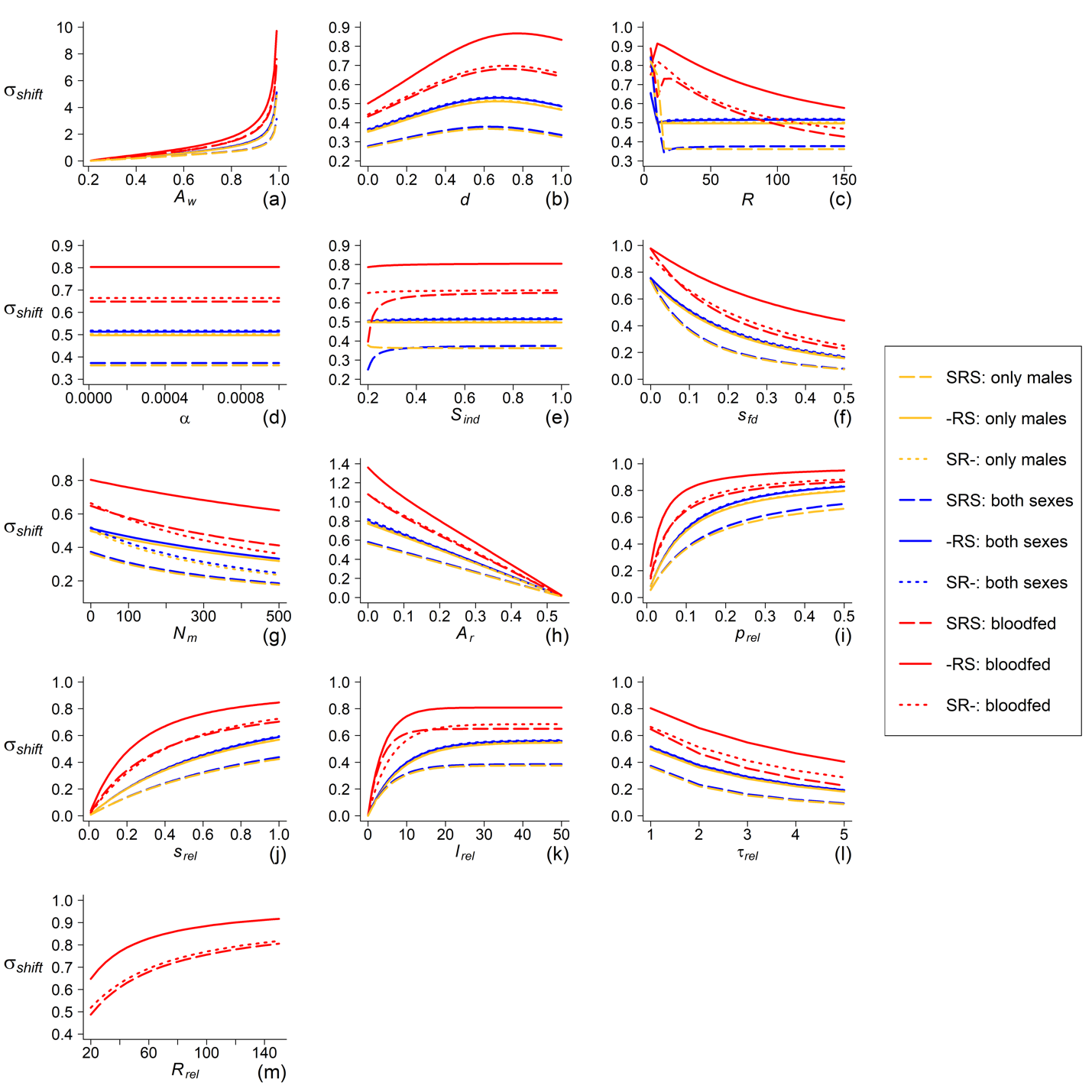


**Figure S18.** Local sensitivity analysis (LSA) of the number of SDs shifted by the VC mean ($\sigma_{shift}$) to each parameter given all other parameters at their default values (see Table S5 for default values and ranges) in the one-locus Mendelian model. Note the difference in the y-axis values across plots. Line types and colors are as in Figure 2.


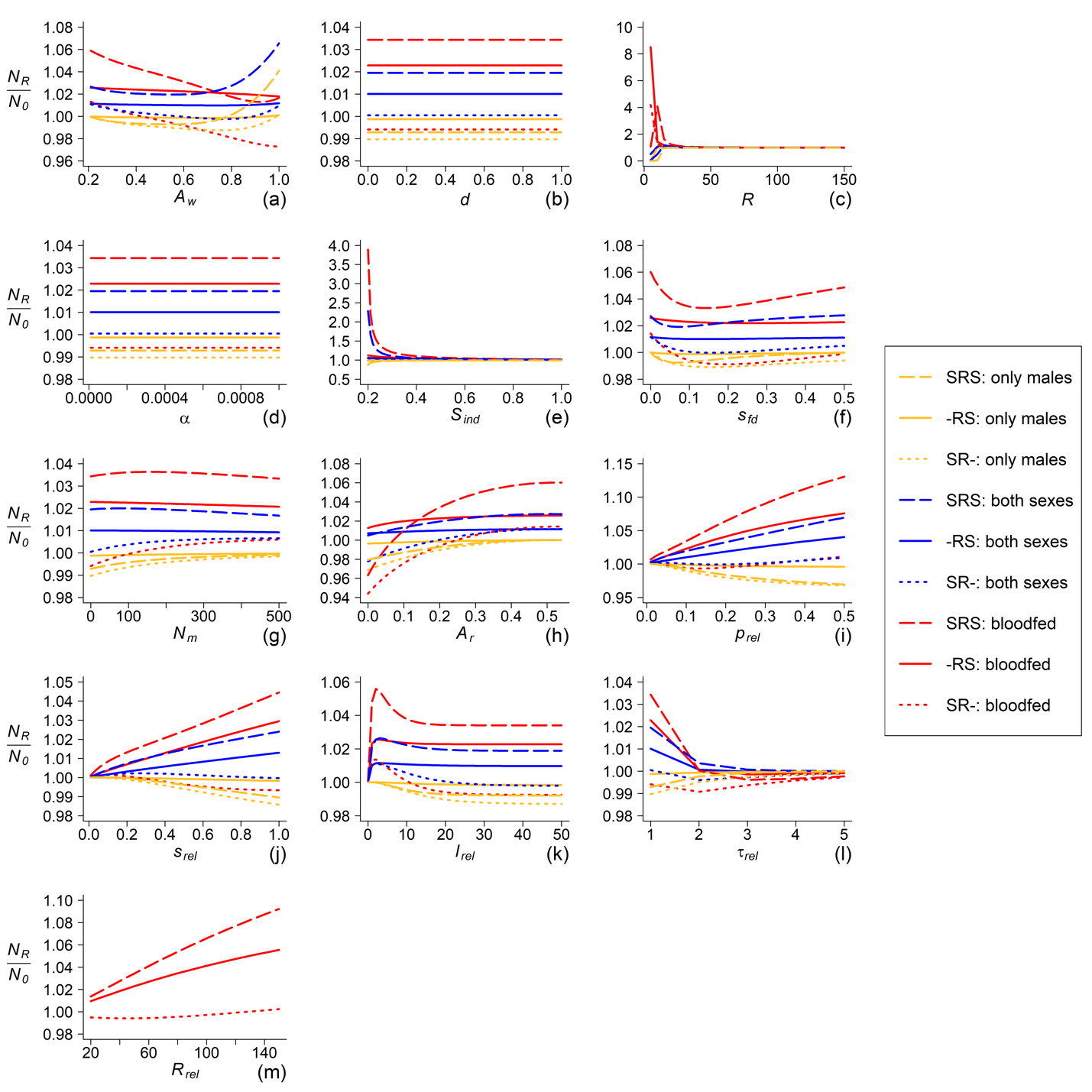


**Figure S19.** Local sensitivity analysis (LSA) of the ratio of population size between the post-release and pre-release population ($N_{R}/N_{0}$) to each parameter given all other parameters at their default values (see Table S5 for default values and ranges) in the one-locus Mendelian model. Note the difference in the y-axis values across plots. Line types and colors are as in Figure 2.


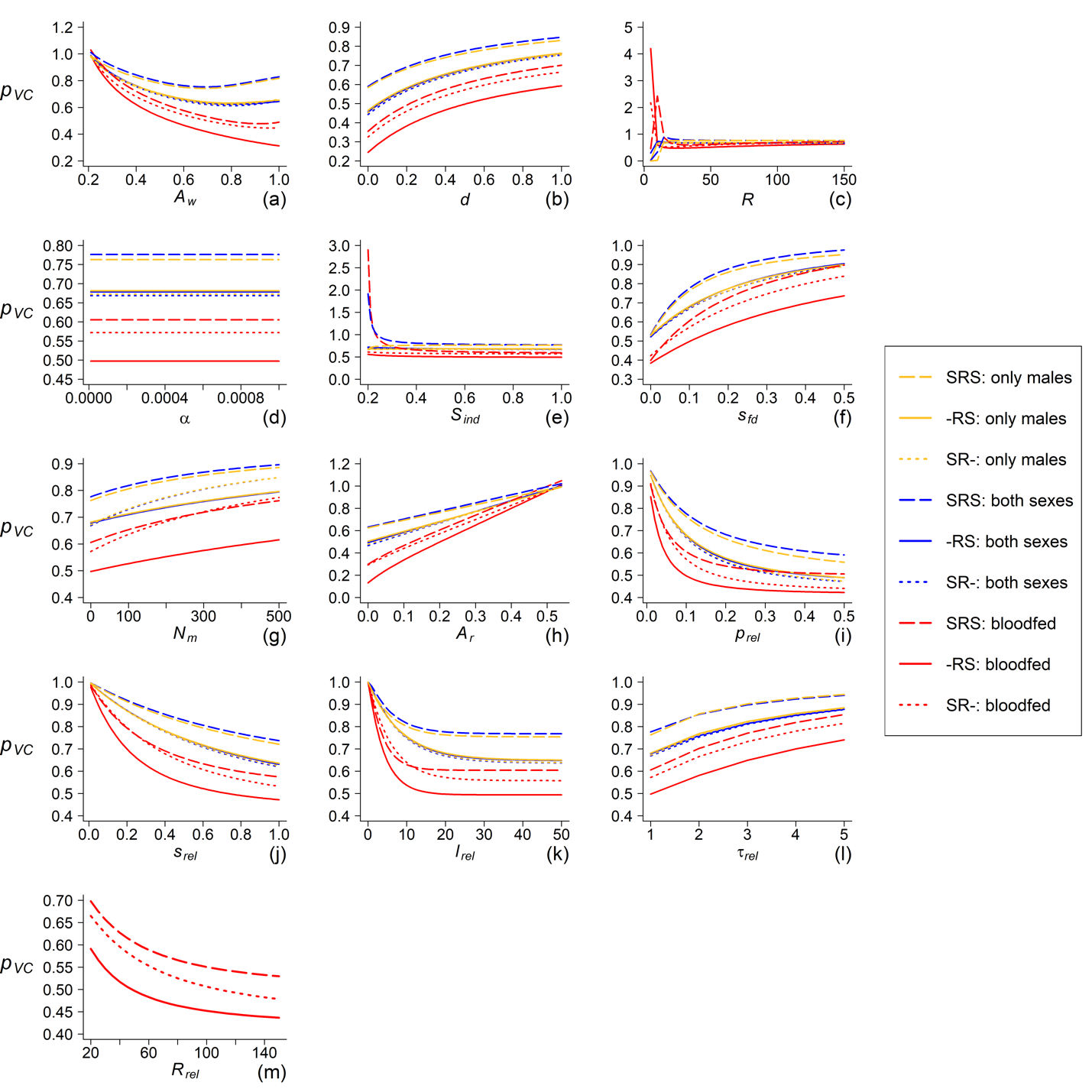


**Figure S20.** Local sensitivity analysis (LSA) of the remaining proportion of integrated VC after releases ($p_{VC}$) to each parameter given all other parameters at their default values (see Table S5 for default values and ranges) in the one-locus Mendelian model. Note the difference in the y-axis values across plots. Line types and colors are as in Figure 2.

**Table S6.** Friedman rank sum test comparing all model scenarios for all four efficacy metrics

| Efficacy metrics | ꭓ^2^ | df | p |
| --- | --- | --- | --- |
| $\mu_{shift}$ | 665,990 | 8 | <0.001 |
| $\sigma_{shift}$ | 665,160 | 8 | <0.001 |
| $N_{R}/N_{0}$ | 121,890 | 8 | <0.001 |
| $p_{VC}$ | 527,140 | 8 | <0.001 |

**Table S7.** Post-hoc pairwise comparisons of relative mean VC ($\mu_{shift}$) among model scenarios in the one-locus Mendelian model

| $\bar{\mu_{shift}}$ | SRS  only males | -RS  only males | SR-  only males | SRS  both sexes | -RS  both sexes | SR-  both sexes | SRS  bloodfed | -RS  bloodfed | SR-  bloodfed |
| --- | --- | --- | --- | --- | --- | --- | --- | --- | --- |
| SRS  only males | 0.955^*^ | V = 4.89E9^**^ p < 0.001 | V = 4.89E9 p < 0.001 | V = 4.88E9 p < 0.001 | V = 4.89E9 p < 0.001 | V = 4.89E9 p < 0.001 | V = 4.89E9 p < 0.001 | V = 4.90E9 p < 0.001 | V = 4.88E9 p < 0.001 |
| -RS  only males |  | 0.909 | V = 8.11E7 p < 0.001 | V = 1.04E7 p < 0.001 | V = 4.89E9 p < 0.001 | V = 4.27E8 p < 0.001 | V = 2.71E9 p < 0.001 | V = 4.89E9 p < 0.001 | V = 2.99E9 p < 0.001 |
| SR-  only males |  |  | 0.931 | V = 7.44E7 p < 0.001 | V = 4.88E9 p < 0.001 | V = 4.89E9 p < 0.001 | V = 4.18E9 p < 0.001 | V = 4.90E9 p < 0.001 | V = 4.60E9 p < 0.001 |
| SRS  both sexes |  |  |  | 0.951 | V = 4.89E9 p < 0.001 | V = 4.89E9 p < 0.001 | V = 4.87E9 p < 0.001 | V = 4.90E9 p < 0.001 | V = 4.86E9 p < 0.001 |
| -RS  both sexes |  |  |  |  | 0.9 | V = 8.15E7 p < 0.001 | V = 2.31E9 p < 0.001 | V = 4.88E9 p < 0.001 | V = 2.57E9 p < 0.001 |
| SR-  both sexes |  |  |  |  |  | 0.924 | V = 3.93E9 p < 0.001 | V = 4.90E9 p < 0.001 | V = 4.40E9 p < 0.001 |
| SRS  bloodfed |  |  |  |  |  |  | 0.887 | V = 4.90E9 p < 0.001 | V = 2.81E9 p < 0.001 |
| -RS  bloodfed |  |  |  |  |  |  |  | 0.785 | V = 9.90E4 p < 0.001 |
| SR-  bloodfed |  |  |  |  |  |  |  |  | 0.884 |

^*^ Values in the diagonal are the mean $\mu_{shift}$ of scenarios calculated from all GSA simulations.

^**^ Wilcoxon signed rank tests with Bonferroni correction.

**Table S8.** Post-hoc pairwise comparisons of number of SDs shifted ($\sigma_{shift}$) among model scenarios in the one-locus Mendelian model

| $\bar{\sigma_{shift}}$ | SRS  only males | -RS  only males | SR-  only males | SRS  both sexes | -RS  both sexes | SR-  both sexes | SRS  bloodfed | -RS  bloodfed | SR-  bloodfed |
| --- | --- | --- | --- | --- | --- | --- | --- | --- | --- |
| SRS  only males | 0.051^*^ | V = 5.43E6^**^ p < 0.001 | V = 6.16E6 p < 0.001 | V = 1.47E7 p < 0.001 | V = 5.59E6 p < 0.001 | V = 6.30E6 p < 0.001 | V = 1.47E7 p < 0.001 | V = 6.44E5 p < 0.001 | V = 2.40E7 p < 0.001 |
| -RS  only males |  | 0.105 | V = 4.80E9 p < 0.001 | V = 4.88E9 p < 0.001 | V = 7.03E6 p < 0.001 | V = 4.47E9 p < 0.001 | V = 2.28E9 p < 0.001 | V = 8.87E6 p < 0.001 | V = 2.01E9 p < 0.001 |
| SR-  only males |  |  | 0.079 | V = 4.81E9 p < 0.001 | V = 1.54E7 p < 0.001 | V = 6.47E6 p < 0.001 | V = 7.47E8 p < 0.001 | V = 1.00E6 p < 0.001 | V = 3.29E8 p < 0.001 |
| SRS  both sexes |  |  |  | 0.057 | V = 6.41E6 p < 0.001 | V = 7.16E6 p < 0.001 | V = 3.76E7 p < 0.001 | V = 1.20E6 p < 0.001 | V = 4.63E7 p < 0.001 |
| -RS  both sexes |  |  |  |  | 0.116 | V = 4.80E9 p < 0.001 | V = 2.66E9 p < 0.001 | V = 2.76E7 p < 0.001 | V = 2.42E9 p = 0.0443 |
| SR-  both sexes |  |  |  |  |  | 0.087 | V = 9.99E8 p < 0.001 | V = 1.73E6 p < 0.001 | V = 5.30E8 p < 0.001 |
| SRS  bloodfed |  |  |  |  |  |  | 0.133 | V = 1.96E6 p < 0.001 | V = 2.10E9 p < 0.001 |
| -RS  bloodfed |  |  |  |  |  |  |  | 0.255 | V = 4.89E9 p < 0.001 |
| SR-  bloodfed |  |  |  |  |  |  |  |  | 0.136 |

^*^ Values in the diagonal are the mean $\sigma_{shift}$ of scenarios calculated from all GSA simulations.

^**^ Wilcoxon signed rank tests with Bonferroni correction.

**Table S9.** Post-hoc pairwise comparisons of population size ratio ($N_{R}/N_{0}$) among model scenarios in the one-locus Mendelian model

| $\bar{\frac{N_{R}}{N_{0}}}$ | SRS  only males | -RS  only males | SR-  only males | SRS  both sexes | -RS  both sexes | SR-  both sexes | SRS  bloodfed | -RS  bloodfed | SR-  bloodfed |
| --- | --- | --- | --- | --- | --- | --- | --- | --- | --- |
| SRS  only males | 1.001^*^ | V = 6.55E8^**^ p < 0.001 | V = 3.75E8 p < 0.001 | V = 9.50E6 p < 0.001 | V = 5.21E8 p < 0.001 | V = 2.68E8 p < 0.001 | V = 2.48E8 p < 0.001 | V = 4.58E8 p < 0.001 | V = 4.83E8 p < 0.001 |
| -RS  only males |  | 1.000 | V = 5.18E8 p < 0.001 | V = 1.30E8 p < 0.001 | V = 1.73E6 p < 0.001 | V = 3.67E8 p < 0.001 | V = 3.23E8 p < 0.001 | V = 9.92E7 p < 0.001 | V = 5.45E8 p < 0.001 |
| SR-  only males |  |  | 1.001 | V = 1.62E8 p < 0.001 | V = 6.17E8 p < 0.001 | V = 5.11E7 p < 0.001 | V = 1.68E8 p < 0.001 | V = 5.39E8 p < 0.001 | V = 3.23E8 p < 0.001 |
| SRS  both sexes |  |  |  | 1.007 | V = 1.09E9 p < 0.001 | V = 1.12E9 p < 0.001 | V = 3.83E8 p < 0.001 | V = 7.55E8 p < 0.001 | V = 1.06E9 p < 0.001 |
| -RS  both sexes |  |  |  |  | 1.004 | V = 9.02E8 p < 0.001 | V = 3.82E8 p < 0.001 | V = 1.49E8 p < 0.001 | V = 7.85E8 p < 0.001 |
| SR-  both sexes |  |  |  |  |  | 1.004 | V = 1.76E8 p < 0.001 | V = 6.06E8 p < 0.001 | V = 4.75E8 p < 0.001 |
| SRS  bloodfed |  |  |  |  |  |  | 1.031 | V = 1.51E9 p < 0.001 | V = 1.54E9 p < 0.001 |
| -RS  bloodfed |  |  |  |  |  |  |  | 1.024 | V = 1.39E9 p < 0.001 |
| SR-  bloodfed |  |  |  |  |  |  |  |  | 1.011 |

^*^ Values in the diagonal are the mean $N_{R}/N_{0}$ of scenarios calculated from all GSA simulations.

^**^ Wilcoxon signed rank tests with Bonferroni correction.

**Table S10.** Post-hoc pairwise comparisons of proportion of integrated VC ($p_{VC}$) among model scenarios in the one-locus Mendelian model

| $\bar{p_{VC}}$ | SRS  only males | -RS  only males | SR-  only males | SRS  both sexes | -RS  both sexes | SR-  both sexes | SRS  bloodfed | -RS  bloodfed | SR-  bloodfed |
| --- | --- | --- | --- | --- | --- | --- | --- | --- | --- |
| SRS  only males | 0.977^*^ | V = 4.98E9^**^ p < 0.001 | V = 4.91E9 p < 0.001 | V = 2.22E9 p < 0.001 | V = 4.78E9 p < 0.001 | V = 4.60E9 p < 0.001 | V = 4.02E9 p < 0.001 | V = 4.79E9 p < 0.001 | V = 4.64E9 p < 0.001 |
| -RS  only males |  | 0.952 | V = 2.01E8 p < 0.001 | V = 1.37E7 p < 0.001 | V = 3.64E9 p < 0.001 | V = 3.66E8 p < 0.001 | V = 1.49E9 p < 0.001 | V = 4.73E9 p < 0.001 | V = 2.23E9 p < 0.001 |
| SR-  only males |  |  | 0.965 | V = 8.21E7 p < 0.001 | V = 4.52E9 p < 0.001 | V = 3.46E9 p < 0.001 | V = 3.00E9 p < 0.001 | V = 4.77E9 p < 0.001 | V = 4.19E9 p < 0.001 |
| SRS  both sexes |  |  |  | 0.981 | V = 4.93E9 p < 0.001 | V = 4.91E9 p < 0.001 | V = 4.19E9 p < 0.001 | V = 4.84E9 p < 0.001 | V = 4.76E9 p < 0.001 |
| -RS  both sexes |  |  |  |  | 0.951 | V = 3.74E8 p < 0.001 | V = 1.31E9 p < 0.001 | V = 4.76E9 p < 0.001 | V = 2.14E9 p < 0.001 |
| SR-  both sexes |  |  |  |  |  | 0.964 | V = 2.85E9 p < 0.001 | V = 4.80E9 p < 0.001 | V = 4.16E9 p < 0.001 |
| SRS  bloodfed |  |  |  |  |  |  | 0.967 | V = 4.93E9 p < 0.001 | V = 3.70E9 p < 0.001 |
| -RS  bloodfed |  |  |  |  |  |  |  | 0.903 | V = 1.94E8 p < 0.001 |
| SR-  bloodfed |  |  |  |  |  |  |  |  | 0.949 |

^*^ Values in the diagonal are the mean $p_{VC}$ of scenarios calculated from all GSA simulations.

^**^ Wilcoxon signed rank tests with Bonferroni correction.
