# Supplementary material for: Quantifying the efficacy of genetic shifting in control of mosquito‐borne diseases
Source: Evol Appl. 2019 Jun 14;12(8):1552–68. doi: 10.1111/eva.12802 (PMC6708429; doi:10.1111/eva.12802)
Supplement: Supplementary file 3 [file EVA-12-1552-s003.docx]

**Appendix 3: Mathematical details and results of the two-locus Mendelian model**

*Mathematical details*

In the two-locus Mendelian model, each of the two loci has two alleles: the susceptible allele (*S_1_* and *S_2_* for locus 1 and 2) and the resistant allele (*R_1_* and *R_2_* for locus 1 and 2). In each locus, the susceptible homozygote has a genotype value of 1; the resistant homozygote has a genotype value of 0; the heterozygote has a genotype of *d_1_* or *d_2_* for locus 1 and 2. Both loci contribute equally to an individual’s VC, that is, the VC of an individual is the arithmetic mean of the genotype values of the two loci. The nine possible genotypes and their VC values are shown in Table S11. The population density distribution *n_i,t_(g)* of population *i* at generation *t* thus composes nine discrete values. The total population size *N_i,_*_t_ = ∑ *n_i,t_(g)*. The allele frequency in each locus is calculated as:

$$\begin{aligned} \left\{ \begin{aligned} {q_{i,t}\left( S_{1} \right)=\frac{\sum n_{i,t}\left( S_{1}S_{1}X_{2}X_{2} \right)+0.5 \sum n_{i,t}\left( S_{1}R_{1}X_{2}X_{2} \right)}{N_{i,t}} \atop q_{i,t}\left( R_{1} \right)=\frac{\sum n_{i,t}\left( R_{1}R_{1}X_{2}X_{2} \right)+0.5 \sum n_{i,t}\left( S_{1}R_{1}X_{2}X_{2} \right)}{N_{i,t}}} \\ {q_{i,t}\left( S_{2} \right)=\frac{\sum n_{i,t}\left( X_{1}X_{1}S_{2}S_{2} \right)+0.5 \sum n_{i,t}\left( X_{1}X_{1}S_{2}R_{2} \right)}{N_{i,t}} \atop q_{i,t}\left( R_{2} \right)=\frac{\sum n_{i,t}\left( X_{1}X_{1}R_{2}R_{2} \right)+0.5 \sum n_{i,t}\left( X_{1}X_{1}S_{2}R_{2} \right)}{N_{i,t}}} \end{aligned} \right.,\#(S11) \end{aligned}$$

where *X* represent either alleles (*S* or *R*).

In the reproduction step, similar to the one-locus Mendelian model (Appendix 2), we separately model males and females and first sum the genotype frequencies in the parental generation into allele frequencies (Equation S11). Then we calculate the genotype frequency distribution in the offspring generation $f_{tgt,t}^{*}\left( g \right)$given random mating (i.e. random encounter of alleles from the father and the mother population) (Table S12). Multiplying this genotype distribution of the target population by the number of offspring per female *R* and the population size of females $N_{tgt, F,t}$ gives rise to the density distribution in the offspring generation: $n_{tgt,t}^{*}\left( g \right)= R N_{tgt, F,t} f_{tgt,t}^{*}\left( g \right)$. When releasing blood-fed females, we model their offspring density distribution separately and then combine it with the population density distribution of offspring in the wild population, as in the one-locus Mendelian model (Appendix 2).

**Table S11.** Vector competence (*g*) and population frequency distribution of all genotypes

| Genotype | Vector competence $g$ | Optimal frequency  $f_{opt}\left( g \right)$ | Release frequency  $f_{rel}(g)$ |
| --- | --- | --- | --- |
| S_1_S_1_S_2_S_2_ | 1 | $A_{w1}^{2}A_{w2}^{2}$ | $A_{r1}^{2}A_{r2}^{2}$ |
| S_1_S_1_S_2_R_2_ | $\frac{1\text{+}d_{2}}{2}$ | $A_{w1}^{2}2A_{w2}(1-A_{w2})$ | $A_{r1}^{2}2A_{r2}(1-A_{r2})$ |
| S_1_S_1_R_2_R_2_ | 0.5 | ${A_{w1}^{2}(1-A_{w2})}^{2}$ | ${A_{r1}^{2}(1-A_{r2})}^{2}$ |
| S_1_R_1_S_2_S_2_ | $\frac{d_{1}\text{+}1}{2}$ | $2A_{w1}(1-A_{w1})A_{w2}^{2}$ | $2A_{r1}(1-A_{r1})A_{r2}^{2}$ |
| S_1_R_1_S_2_R_2_ | $\frac{d_{1}\text{+}d_{2}}{2}$ | $4A_{w1}(1-A_{w1})A_{w2}(1-A_{w2})$ | $4A_{r1}(1-A_{r1})A_{r2}(1-A_{r2})$ |
| S_1_R_1_R_2_R_2_ | $\frac{d_{1}}{2}$ | $2A_{w1}(1-A_{w1}){(1-A_{w2})}^{2}$ | $2A_{r1}(1-A_{r1}){(1-A_{r2})}^{2}$ |
| R_1_R_1_S_2_S_2_ | 0.5 | ${(1-A_{w1})}^{2}A_{w2}^{2}$ | ${(1-A_{r1})}^{2}A_{r2}^{2}$ |
| R_1_R_1_S_2_R_2_ | $\frac{d_{2}}{2}$ | ${(1-A_{w1})}^{2}2A_{w2}(1-A_{w2})$ | ${(1-A_{r1})}^{2}2A_{r2}(1-A_{r2})$ |
| R_1_R_1_R_2_R_2_ | 0 | ${(1-A_{w1})}^{2}{(1-A_{w2})}^{2}$ | ${(1-A_{r1})}^{2}{(1-A_{r2})}^{2}$ |

**Table S12.** Offspring genotype frequency calculated from the parental allele frequency

| Genotype | Offspring genotype frequency $f_{i,t}^{*}\left( g \right)$ |
| --- | --- |
| S_1_S_1_S_2_S_2_ | $q_{i, F,t}\left( S_{1} \right)q_{i, M,t}\left( S_{1} \right)q_{i, F,t}\left( S_{2} \right)q_{i, M,t}\left( S_{2} \right)$ |
| S_1_S_1_S_2_R_2_ | $q_{i, F,t}\left( S_{1} \right)q_{i, M,t}\left( S_{1} \right)\left( q_{i,F,t}\left( S_{2} \right)q_{i,M,t}\left( R_{2} \right)+q_{i,F,t}\left( R_{2} \right)q_{i,M,t}\left( S_{2} \right) \right)$ |
| S_1_S_1_R_2_R_2_ | $q_{i, F,t}\left( S_{1} \right)q_{i, M,t}\left( S_{1} \right)q_{i, F,t}\left( R_{2} \right)q_{i, M,t}\left( R_{2} \right)$ |
| S_1_R_1_S_2_S_2_ | $\left( q_{i,F,t}\left( S_{1} \right)q_{i,M,t}\left( R_{1} \right)+q_{i,F,t}\left( R_{1} \right)q_{i,M,t}\left( S_{1} \right) \right)q_{i, F,t}\left( S_{2} \right)q_{i, M,t}\left( S_{2} \right)$ |
| S_1_R_1_S_2_R_2_ | $\left( q_{i,F,t}\left( S_{1} \right)q_{i,M,t}\left( R_{1} \right)+q_{i,F,t}\left( R_{1} \right)q_{i,M,t}\left( S_{1} \right) \right)\left( q_{i,F,t}\left( S_{2} \right)q_{i,M,t}\left( R_{2} \right)+q_{i,F,t}\left( R_{2} \right)q_{i,M,t}\left( S_{2} \right) \right)$ |
| S_1_R_1_R_2_R_2_ | $\left( q_{i,F,t}\left( S_{1} \right)q_{i,M,t}\left( R_{1} \right)+q_{i,F,t}\left( R_{1} \right)q_{i,M,t}\left( S_{1} \right) \right)q_{i, F,t}\left( R_{2} \right)q_{i, M,t}\left( R_{2} \right)$ |
| R_1_R_1_S_2_S_2_ | $q_{i, F,t}\left( R_{1} \right)q_{i, M,t}\left( R_{1} \right)q_{i, F,t}\left( S_{2} \right)q_{i, M,t}\left( S_{2} \right)$ |
| R_1_R_1_S_2_R_2_ | $q_{i, F,t}\left( R_{1} \right)q_{i, M,t}\left( R_{1} \right)\left( q_{i,F,t}\left( S_{2} \right)q_{i,M,t}\left( R_{2} \right)+q_{i,F,t}\left( R_{2} \right)q_{i,M,t}\left( S_{2} \right) \right)$ |
| R_1_R_1_R_2_R_2_ | $q_{i, F,t}\left( R_{1} \right)q_{i, M,t}\left( R_{1} \right)q_{i, F,t}\left( R_{2} \right)q_{i, M,t}\left( R_{2} \right)$ |

We model density-dependent survival, density-independent survival, frequency-dependent selection and release-migration the same as in the one-locus Mendelian model (Appendix 2). The optimal genotype frequency in the wild $f_{opt}(g)$ and the genotype frequency of the release population $f_{rel}(g)$ are shown in Table S11. We numerically implement the model as described in the one-locus Mendelian model. We use the same values and ranges for parameters shared with the quantitative polygenic model and the one-locus Mendelian model. We summarize the release efficacy with the same four metrics ($\mu_{shift}$, $\sigma_{shift}$, $\frac{N_{R}}{N_{0}}$, and $p_{VC}$, Equation S4-S7).

**Table S13.** Descriptions and values of model parameters in the two-locus Mendelian model

| Parameter | Description | Default | Range |
| --- | --- | --- | --- |
| *A_w1_* | Optimal frequency of S_1_ in locus 1 in the wild | 0.6 | 0.1 – 1^+^ |
| *A_w2_* | Optimal frequency of S_2_ in locus 2 in the wild | 0.7 | 0.1 – 1^+^ |
| *d_1_* | Dominance of S_1_ in locus 1 | 0.4 | 0 – 1 |
| *d_2_* | Dominance of S_2_ in locus 2 | 0.6 | 0 – 1 |
| *R* | Mean number of offspring per female | 40 | 5 – 150 |
| *α* | Beverton-Holt density-dependent saturation constant | 10^-4^ | 10^-5^ – 10^-3^ |
| *S_ind_* | Density-independent survival probability | 0.7 | 0.2 – 1 |
| *s_fd_* | Strength of frequency-dependent selection | 0.1^#^ | 0 – 0.5^#^ |
| *N_m_* | Number of immigrants from external population | 0 | 0 – 500 |
| *A_r1_* | Frequency of S_1_ in locus 1 in the release population | 0.2 | (0 – 1) *A_w1_* |
| *A_r2_* | Frequency of S_2_ in locus 2 in the release population | 0.3 | (0 – 1) *A_w2_* |
| *p_rel,t_* | Relative size of the releasing population at generation *t* | 0.1*^++^* | 0.01 – 0.5*^++^* |
| *s_rel_* | Mean survival probability of releasing individuals | 0.75 | 0.01 – 1 |
| *l_rel_* | Number of releases | 20 | 1 – 50 |
| *τ_rel_* | Release frequency: number of generations between releases | 1 | 1 – 5 |
| *R_rel_* | Reproductive output of blood-fed releasing female | 50 | 20 – 150 |

^+^ In the local sensitivity analysis, *A_w1_* and *A_w2_* ranges from 0.2 – 1 and 0.3 – 1, respectively, as the susceptible allele frequency should be lower in the release population than in the wild population.

^++^ *p_rel,t_* = 0 when no release occurs at generation *t*.

^#^ Little empirical data exist so we determine the value and the range based on experience.

*Results*


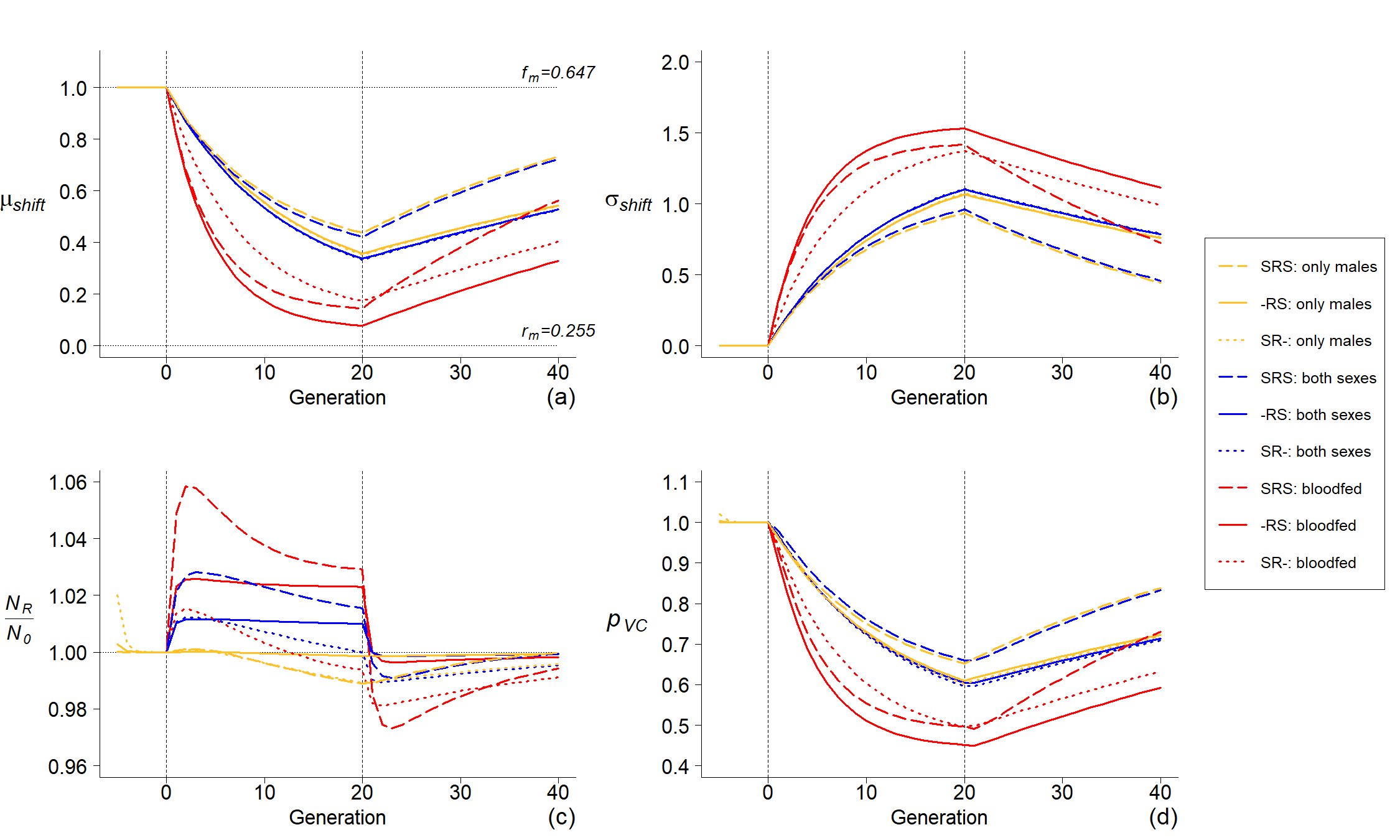


**Figure S21.** Change of VC during 20 generations of releases and 20 generations of recovery in the two-locus Mendelian model. The model followed the changes of (a) relative mean of VC in the post-release population ($\mu_{shift}$)**,** (b) number of SDs shifted by the VC mean ($\sigma_{shift}$), (c) ratio of population size between the post-release and pre-release population ($N_{R}/N_{0}$), and (d) the proportion of remaining integrated VC ($p_{VC}$) in the target population. Line types and colors are as in Figure 2. The first and second dashed vertical lines indicate the start and the end of the releases. Five generations before the release started are also shown to demonstrate the equilibrium state of the pre-release population. The horizontal lines in (a) indicate the selection optimum (*f_m_*) and the mean VC of the release population (*r_m_*). We model all scenarios using the default parameter values in Table S13. Note that the vertical axes do not start from 0 in panels (c) and (d).


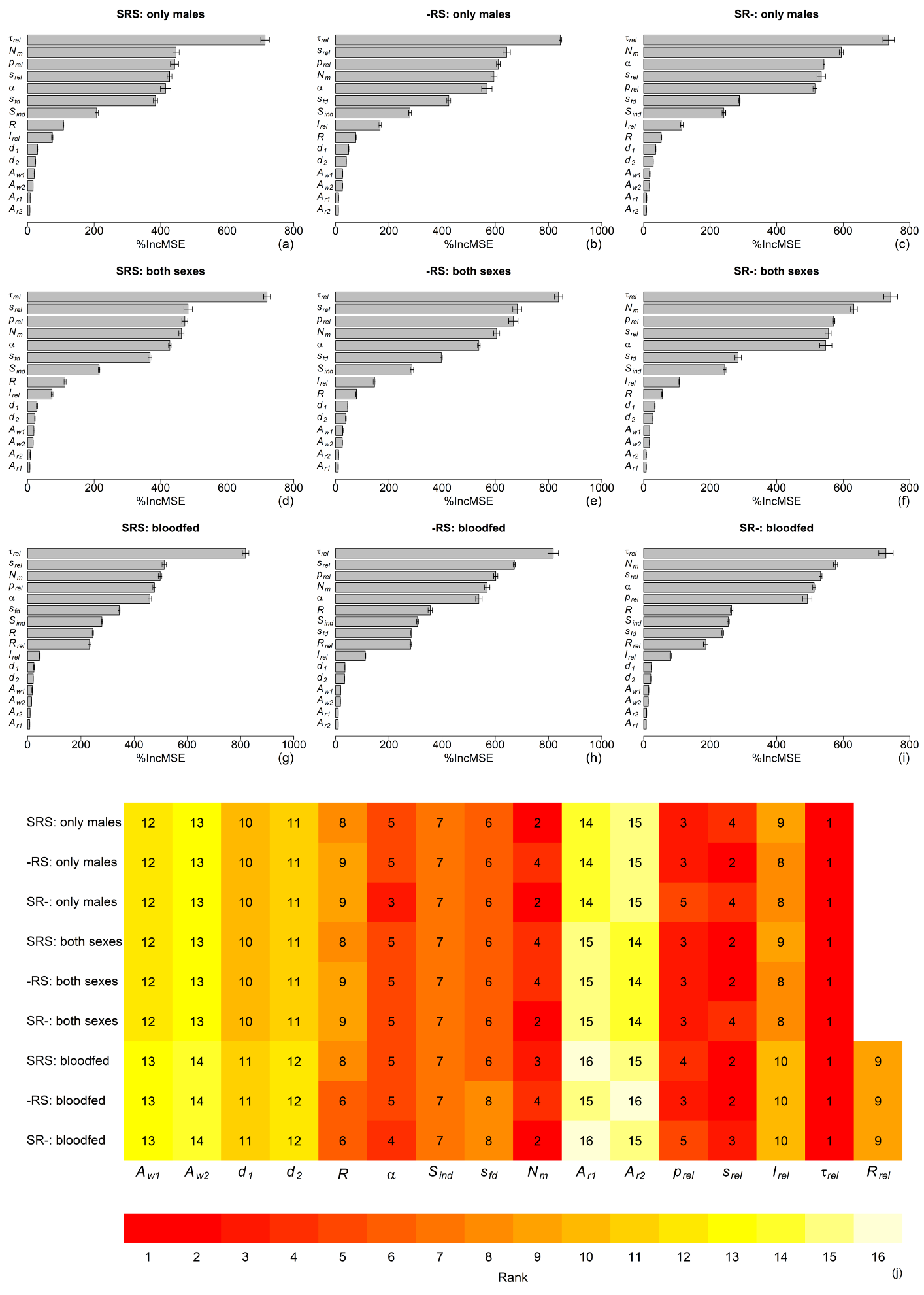


**Figure S22.** Parameter importance (PI) in determining the relative mean of VC in the post-release population ($\mu_{shift}$) in all nine model scenarios in the two-locus Mendelian model. (a)-(i) PI values of all parameters in each scenario. The error bars represent the standard errors calculated from the three replicates. Parameters are ordered decreasingly according to their PI value in each panel. (h) Heat plot of PI ranks in all nine scenarios. Ranks are shown as numbers in grids as well as colors.


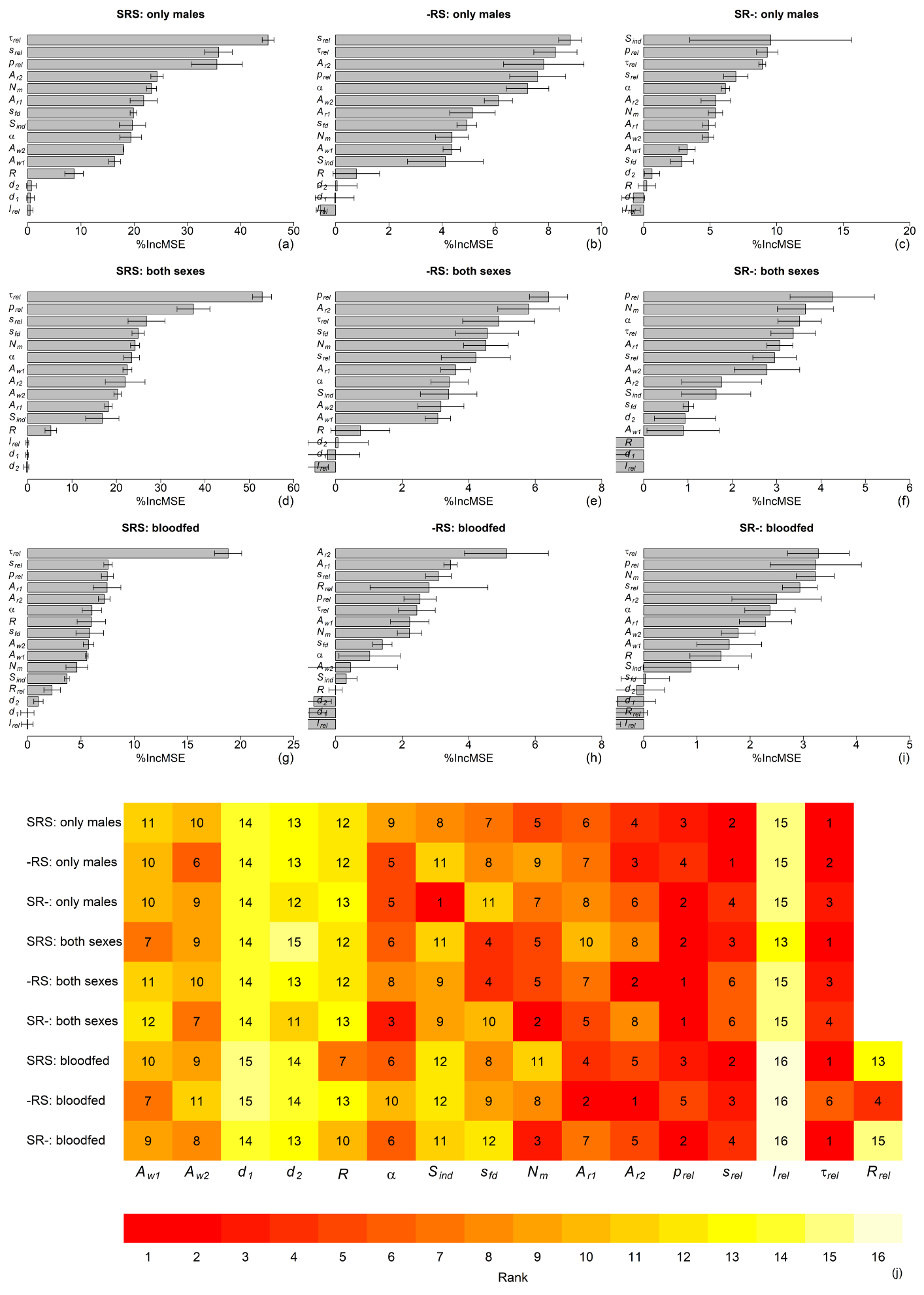


**Figure S23.** Parameter importance (PI) in determining the number of SDs shifted by the VC mean ($\sigma_{shift}$) in all nine model scenarios in the two-locus Mendelian model. (a)-(i) PI values of all parameters in each scenario. The error bars represent the standard errors calculated from the three replicates. Parameters are ordered decreasingly according to their PI value in each panel. (h) Heat plot of PI ranks in all nine scenarios. Ranks are shown as numbers in grids as well as colors.


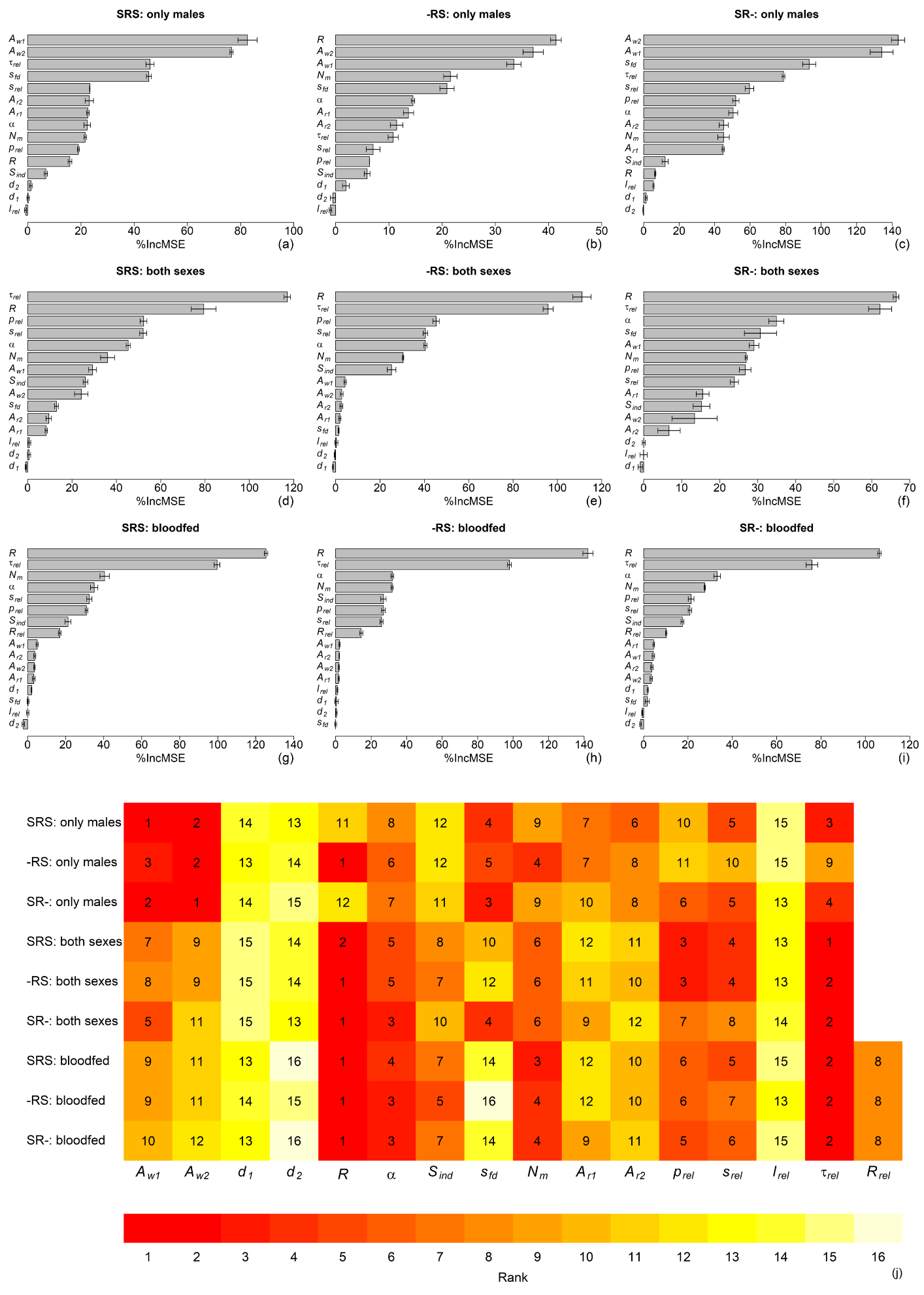


**Figure S24.** Parameter importance (PI) in determining the ratio of population size between the post-release and pre-release population ($N_{R}/N_{0}$) in all nine model scenarios in the two-locus Mendelian model. (a)-(i) PI values of all parameters in each scenario. The error bars represent the standard errors calculated from the three replicates. Parameters are ordered decreasingly according to their PI value in each panel. (h) Heat plot of PI ranks in all nine scenarios. Ranks are shown as numbers in grids as well as colors.

**
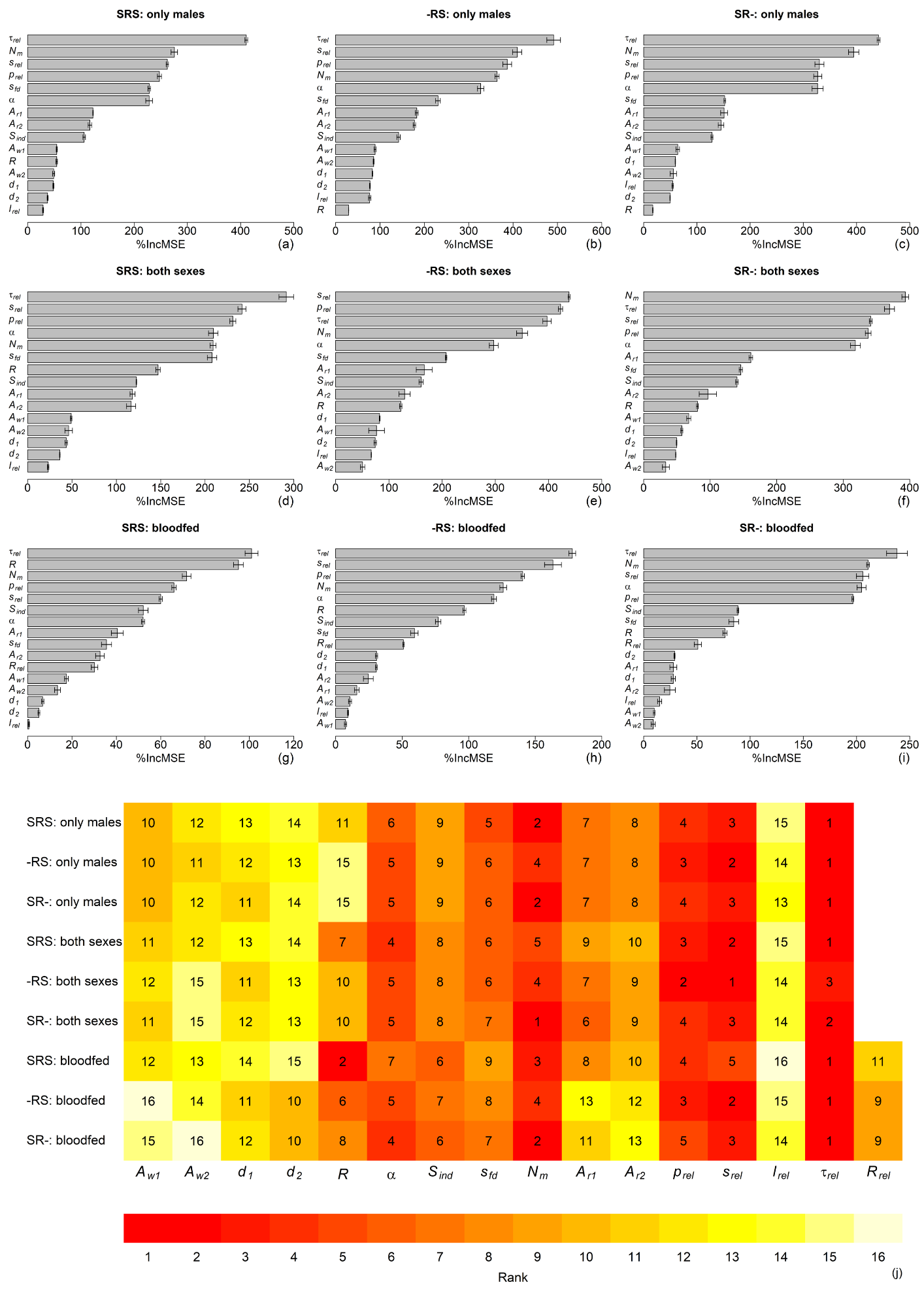
**

**Figure S25.** Parameter importance (PI) in determining the proportion of remaining integrated VC ($p_{VC}$) in all nine model scenarios in the two-locus Mendelian model. (a)-(i) PI values of all parameters in each scenario. The error bars represent the standard errors calculated from the three replicates. Parameters are ordered decreasingly according to their PI value in each panel. (h) Heat plot of PI ranks in all nine scenarios. Ranks are shown as numbers in grids as well as colors.


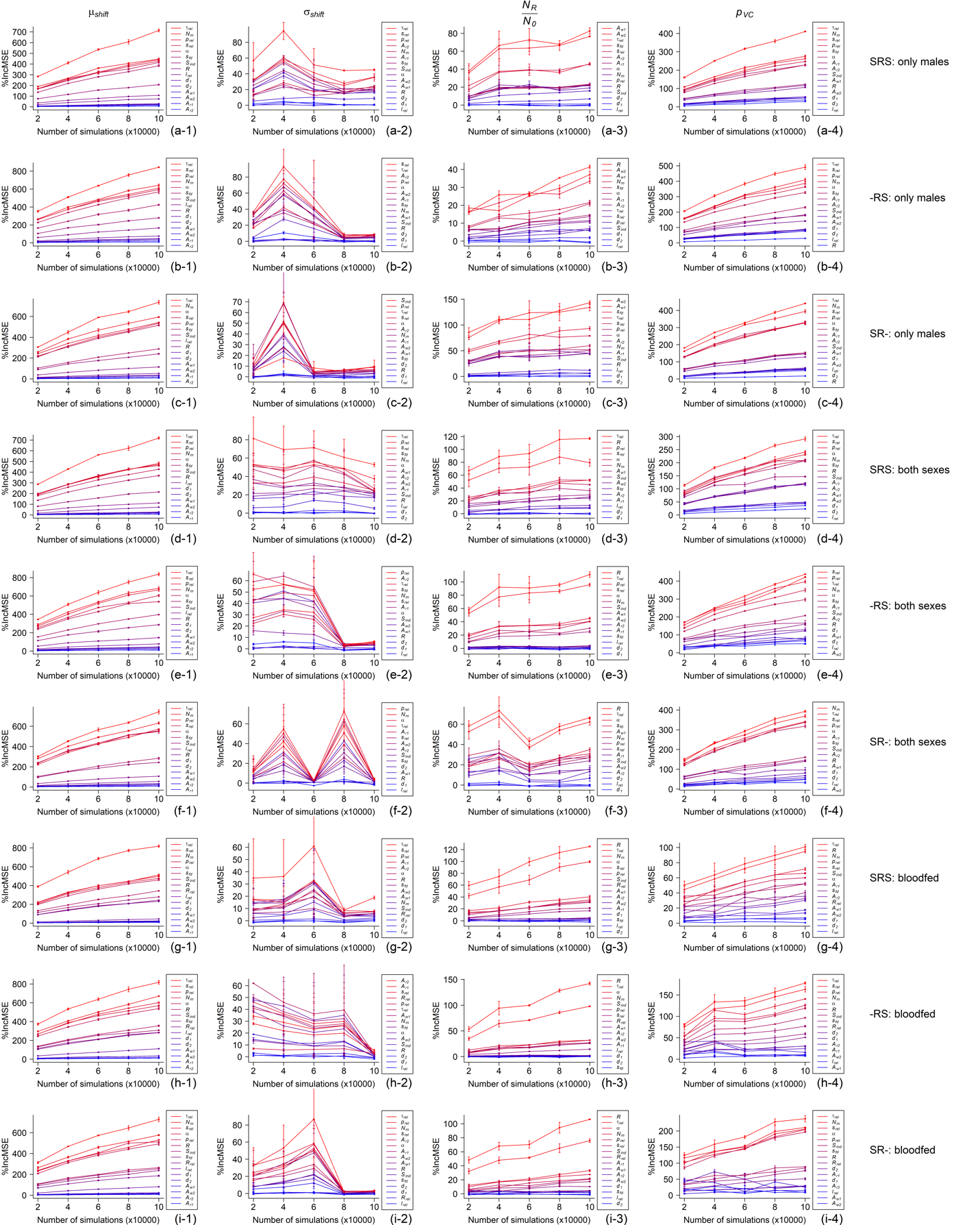


**Figure S26.** Parameter importance (PI) scores calculated with increasing numbers of simulations (20,000, 40,000, 60,000, 80,000, 100,000) in the two-locus Mendelian model. Each row contains results from the same model scenario (combination of release-selection order and release strategy), which is labeled on the right of each row. Each column contains results for the same efficacy metrics. Error bars represent the standard errors calculated from the three replicates. Parameters in each panel are ranked from highest PI (red lines) to lowest PI (blue lines).


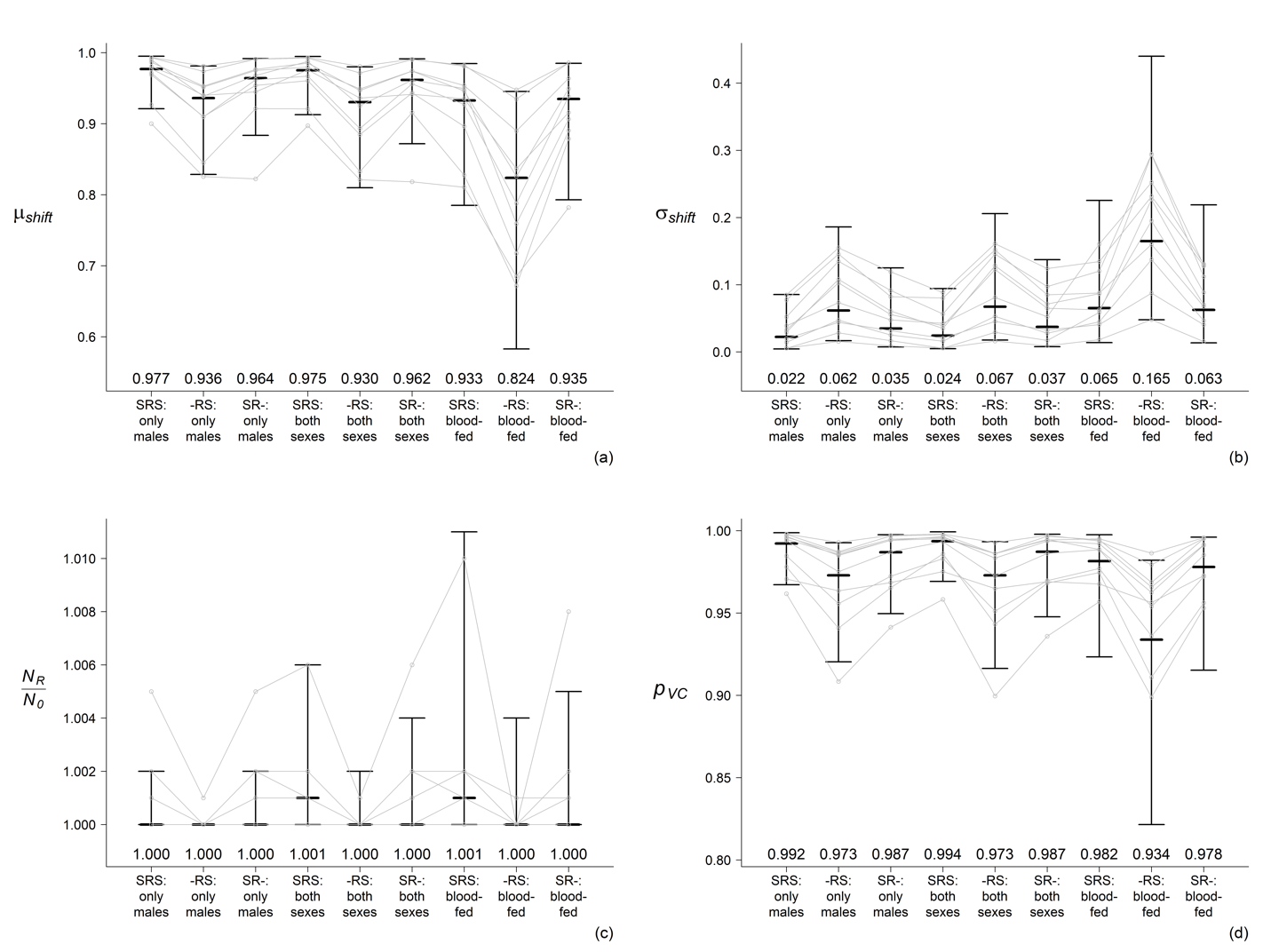


**Figure S27.** Median and interquartile range of each efficacy metrics for all nine model scenarios, calculated at *t* = $l_{rel}\times\tau_{rel}$ from the 100,000 GSA simulations in the two-locus Mendelian model: (a) relative mean of VC in the post-release population ($\mu_{shift}$)**,** (b) number of SDs shifted by the VC mean ($\sigma_{shift}$), (c) ratio of population size between the post-release and pre-release population ($N_{R}/N_{0}$), and (d) the proportion of remaining integrated VC ($p_{VC}$). The three bars in each scenario represent the 25% quantile, the median and the 75% quantile, respectively. The gray lines represent 10 randomly selected simulations. The numbers at the bottom of each figure show the median values of the 100,000 simulations.


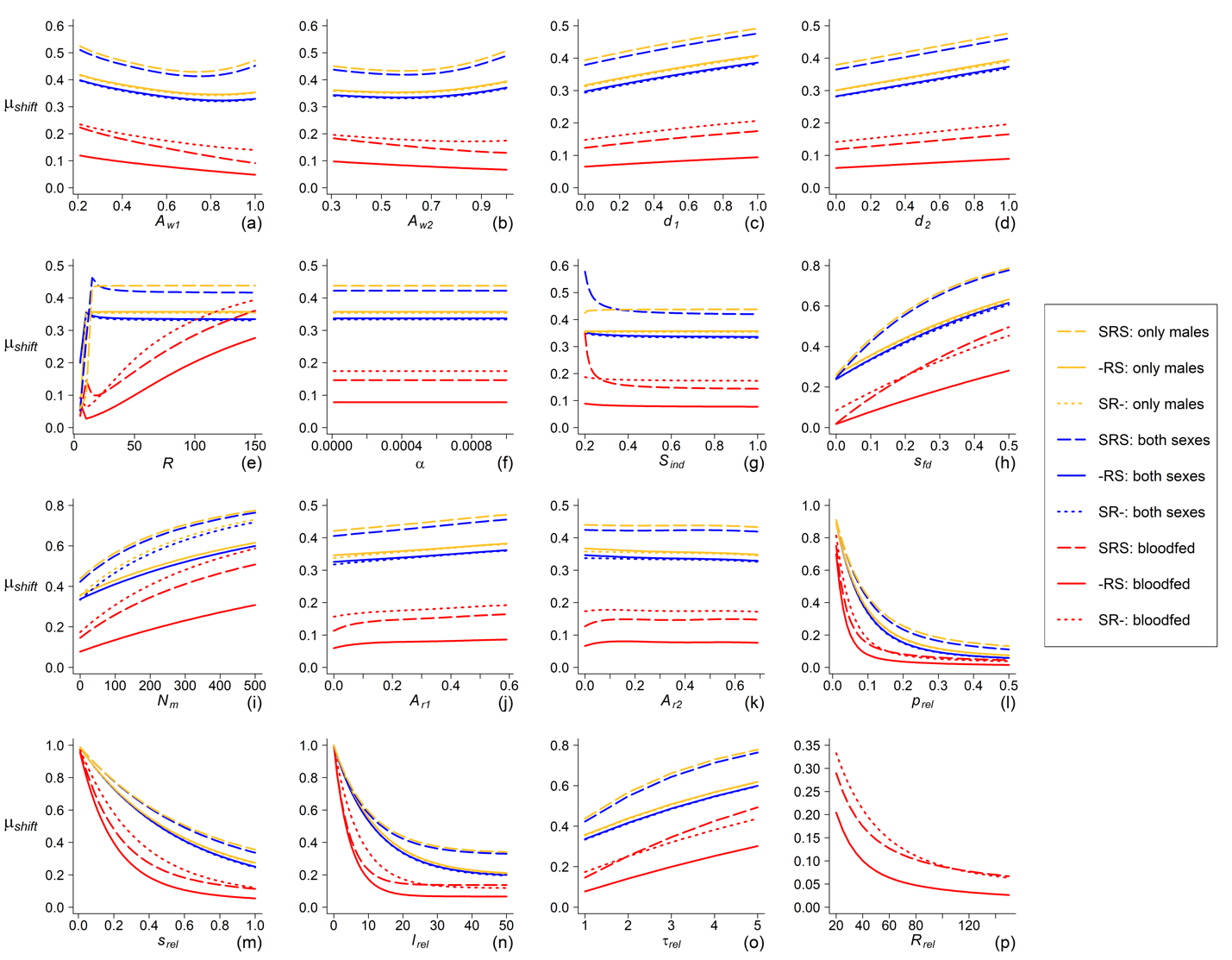


**Figure S28.** Local sensitivity analysis (LSA) of the relative mean of VC in the post-release population ($\mu_{shift}$) to each parameter given all other parameters at their default values (see Table S13 for default values and ranges) in the two-locus Mendelian model. Note the difference in the y-axis values across plots. Line types and colors are as in Figure 2.


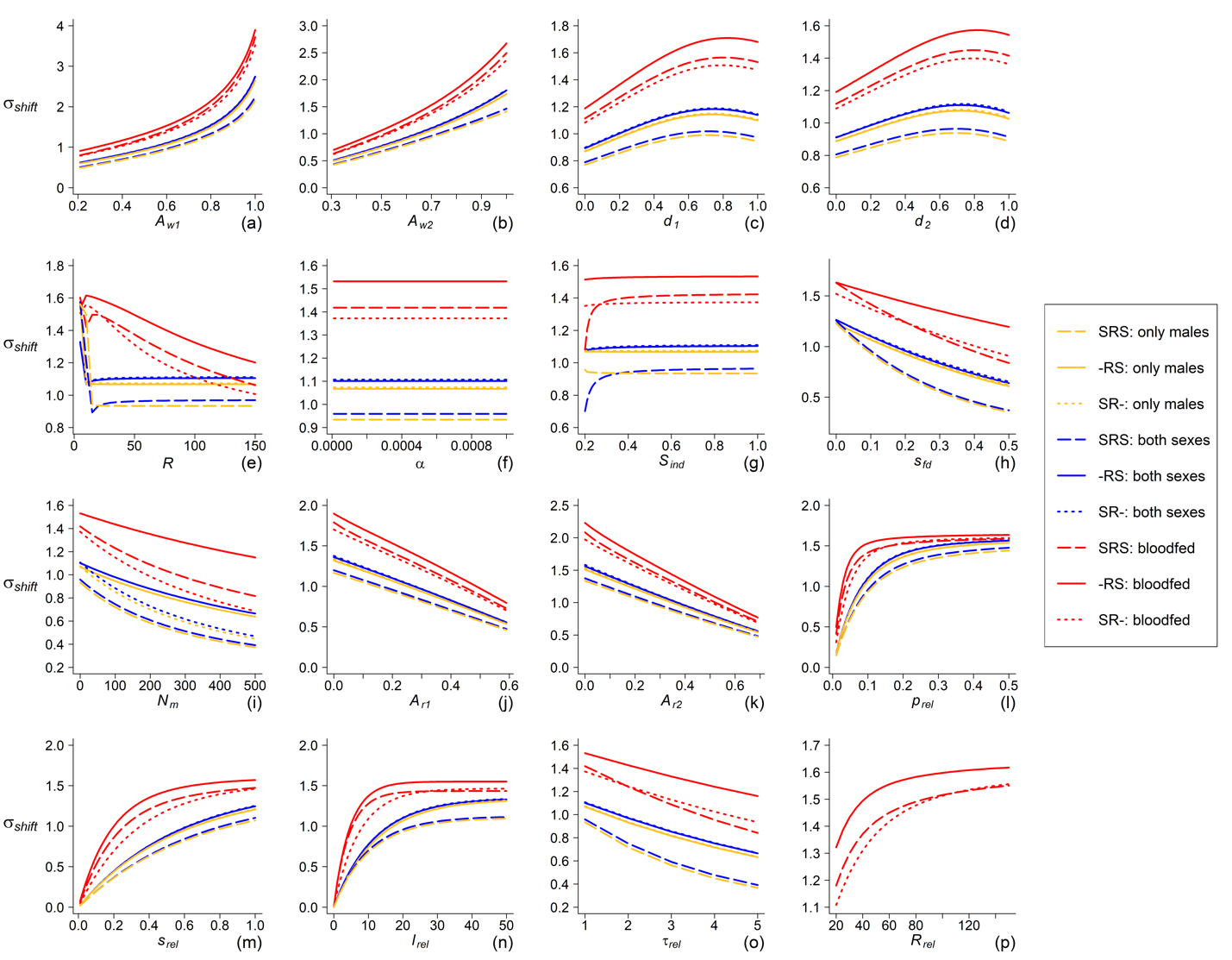


**Figure S29.** Local sensitivity analysis (LSA) of the number of SDs shifted by the VC mean ($\sigma_{shift}$) to each parameter given all other parameters at their default values (see Table S13 for default values and ranges) in the two-locus Mendelian model. Note the difference in the y-axis values across plots. Line types and colors are as in Figure 2.


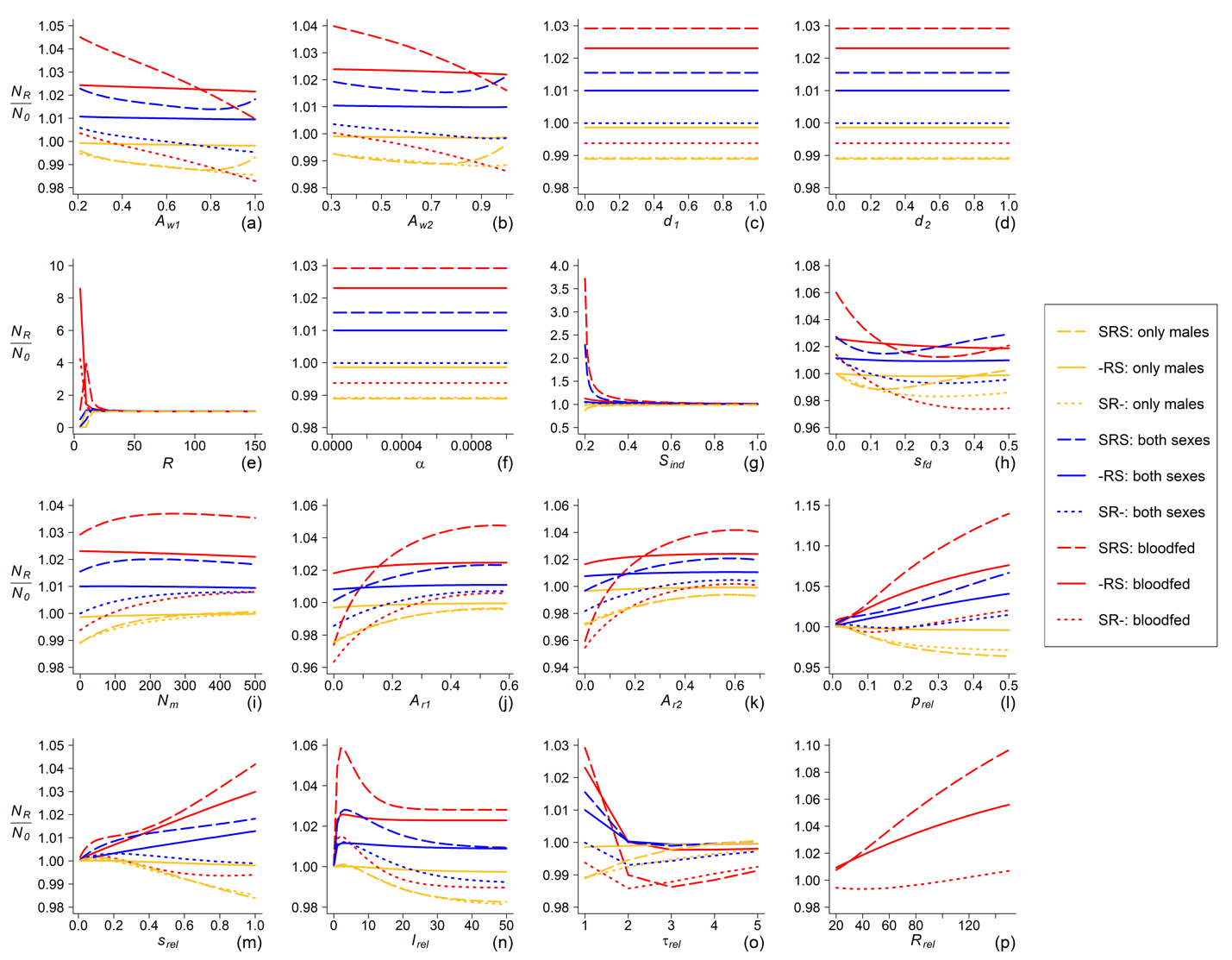


**Figure S30.** Local sensitivity analysis (LSA) of the ratio of population size between the post-release and pre-release population ($N_{R}/N_{0}$) to each parameter given all other parameters at their default values (see Table S13 for default values and ranges) in the two-locus Mendelian model. Note the difference in the y-axis values across plots. Line types and colors are as in Figure 2.


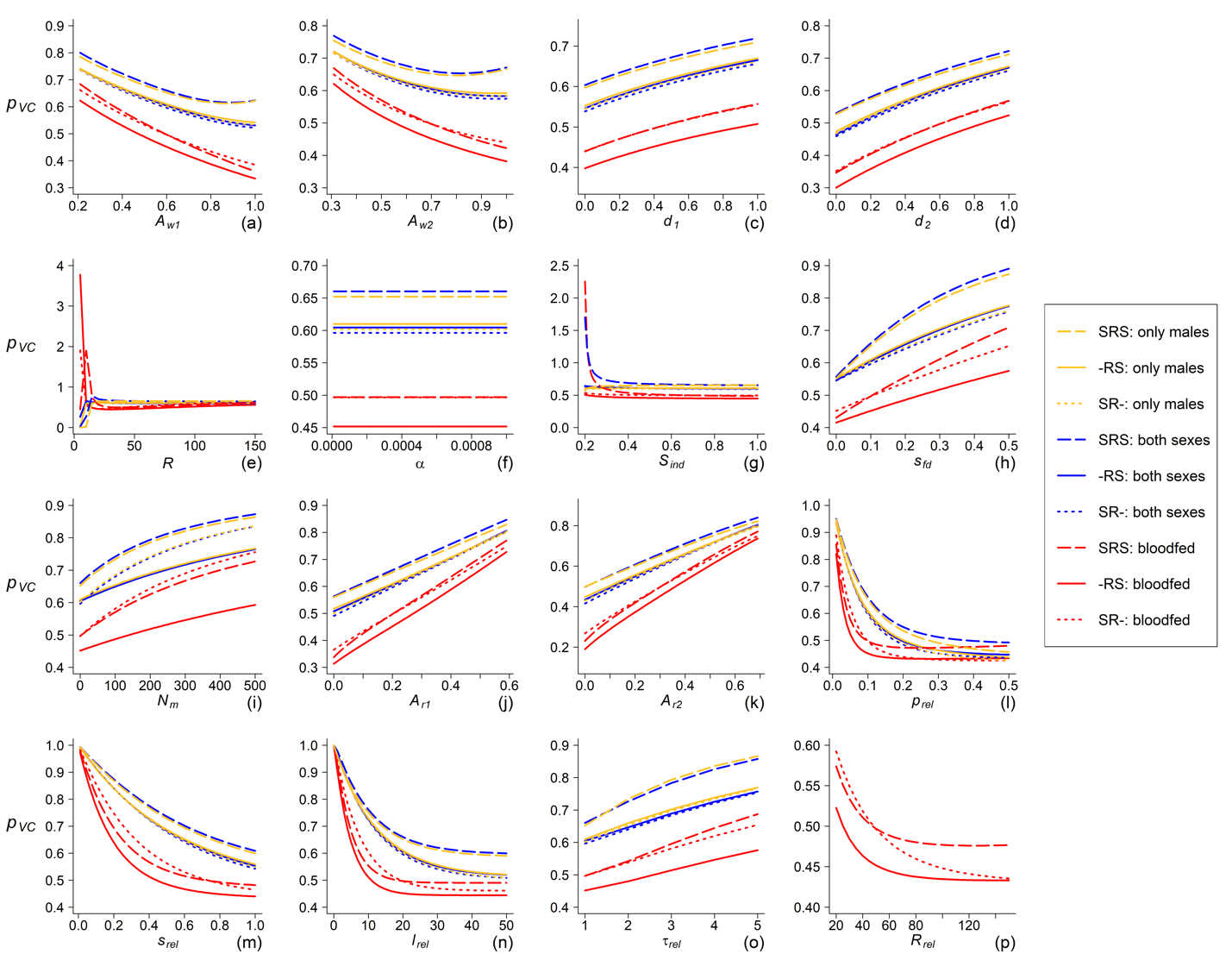


**Figure S31.** Local sensitivity analysis (LSA) of the remaining proportion of integrated VC after releases ($p_{VC}$) to each parameter given all other parameters at their default values (see Table S13 for default values and ranges) in the two-locus Mendelian model. Note the difference in the y-axis values across plots. Line types and colors are as in Figure 2.

**Table S14.** Friedman rank sum test comparing all model scenarios for all four efficacy metrics

| Efficacy metrics | ꭓ^2^ | df | p |
| --- | --- | --- | --- |
| $\mu_{shift}$ | 680,360 | 8 | <0.001 |
| $\sigma_{shift}$ | 680,360 | 8 | <0.001 |
| $N_{R}/N_{0}$ | 119,060 | 8 | <0.001 |
| $p_{VC}$ | 578,960 | 8 | <0.001 |

**Table S15.** Post-hoc pairwise comparisons of relative mean VC ($\mu_{shift}$) among model scenarios in the two-locus Mendelian model

| $\bar{\mu_{shift}}$ | SRS  only males | -RS  only males | SR-  only males | SRS  both sexes | -RS  both sexes | SR-  both sexes | SRS  bloodfed | -RS  bloodfed | SR-  bloodfed |
| --- | --- | --- | --- | --- | --- | --- | --- | --- | --- |
| SRS  only males | 0.932^*^ | V = 4.99E9^**^ p < 0.001 | V = 4.99E9 p < 0.001 | V = 4.99E9 p < 0.001 | V = 4.99E9 p < 0.001 | V = 4.99E9 p < 0.001 | V = 4.98E9 p < 0.001 | V = 5.00E9 p < 0.001 | V = 4.96E9 p < 0.001 |
| -RS  only males |  | 0.874 | V = 3.66E7 p < 0.001 | V = 1.54E7 p < 0.001 | V = 4.99E9 p < 0.001 | V = 2.77E8 p < 0.001 | V = 3.06E9 p < 0.001 | V = 4.99E9 p < 0.001 | V = 2.82E9 p < 0.001 |
| SR-  only males |  |  | 0.907 | V = 1.71E8 p < 0.001 | V = 5.00E9 p < 0.001 | V = 4.99E9 p < 0.001 | V = 4.64E9 p < 0.001 | V = 5.00E9 p < 0.001 | V = 4.70E9 p < 0.001 |
| SRS  both sexes |  |  |  | 0.926 | V = 4.99E9 p < 0.001 | V = 4.99E9 p < 0.001 | V = 4.96E9 p < 0.001 | V = 5.00E9 p < 0.001 | V = 4.92E9 p < 0.001 |
| -RS  both sexes |  |  |  |  | 0.862 | V = 3.57E7 p < 0.001 | V = 2.60E9 p < 0.001 | V = 4.97E9 p < 0.001 | V = 2.38E9 p < 0.001 |
| SR-  both sexes |  |  |  |  |  | 0.898 | V = 4.45E9 p < 0.001 | V = 5.00E9 p < 0.001 | V = 4.49E9 p < 0.001 |
| SRS  bloodfed |  |  |  |  |  |  | 0.846 | V = 5.00E9 p < 0.001 | V = 1.89E9 p < 0.001 |
| -RS  bloodfed |  |  |  |  |  |  |  | 0.734 | V = 3.83E4 p < 0.001 |
| SR-  bloodfed |  |  |  |  |  |  |  |  | 0.849 |

^*^ Values in the diagonal are the mean $\mu_{shift}$ of scenarios calculated from all GSA simulations.

^**^ Wilcoxon signed rank tests with Bonferroni correction.

**Table S16.** Post-hoc pairwise comparisons of number of SDs shifted ($\sigma_{shift}$) among model scenarios in the two-locus Mendelian model

| $\bar{\sigma_{shift}}$ | SRS  only males | -RS  only males | SR-  only males | SRS  both sexes | -RS  both sexes | SR-  both sexes | SRS  bloodfed | -RS  bloodfed | SR-  bloodfed |
| --- | --- | --- | --- | --- | --- | --- | --- | --- | --- |
| SRS  only males | 0.086^*^ | V = 8.62E6^**^ p < 0.001 | V = 9.66E6 p < 0.001 | V = 1.20E7 p < 0.001 | V = 8.63E6 p < 0.001 | V = 9.64E6 p < 0.001 | V = 1.62E7 p < 0.001 | V = 9.58E5 p < 0.001 | V = 4.27E7 p < 0.001 |
| -RS  only males |  | 0.162 | V = 4.96E9 p < 0.001 | V = 4.98E9 p < 0.001 | V = 5.06E6 p < 0.001 | V = 4.72E9 p < 0.001 | V = 1.98E9 p < 0.001 | V = 9.96E6 p < 0.001 | V = 2.23E9 p < 0.001 |
| SR-  only males |  |  | 0.120 | V = 4.82E9 p < 0.001 | V = 4.03E6 p < 0.001 | V = 4.75E6 p < 0.001 | V = 3.75E8 p < 0.001 | V = 6.14E5 p < 0.001 | V = 3.16E8 p < 0.001 |
| SRS  both sexes |  |  |  | 0.095 | V = 8.72E6 p < 0.001 | V = 9.74E6 p < 0.001 | V = 4.55E7 p < 0.001 | V = 1.46E6 p < 0.001 | V = 8.48E7 p < 0.001 |
| -RS  both sexes |  |  |  |  | 0.178 | V = 4.96E9 p < 0.001 | V = 2.44E9 p < 0.001 | V = 3.33E7 p < 0.001 | V = 2.67E9 p < 0.001 |
| SR-  both sexes |  |  |  |  |  | 0.131 | V = 5.67E8 p < 0.001 | V = 1.58E6 p < 0.001 | V = 5.25E8 p < 0.001 |
| SRS  bloodfed |  |  |  |  |  |  | 0.200 | V = 2.32E6 p < 0.001 | V = 3.11E9 p < 0.001 |
| -RS  bloodfed |  |  |  |  |  |  |  | 0.348 | V = 5.00E9 p < 0.001 |
| SR-  bloodfed |  |  |  |  |  |  |  |  | 0.197 |

^*^ Values in the diagonal are the mean $\sigma_{shift}$ of scenarios calculated from all GSA simulations.

^**^ Wilcoxon signed rank tests with Bonferroni correction.

**Table S17.** Post-hoc pairwise comparisons of population size ratio ($N_{R}/N_{0}$) among model scenarios in the two-locus Mendelian model

| $\bar{\frac{N_{R}}{N_{0}}}$ | SRS  only males | -RS  only males | SR-  only males | SRS  both sexes | -RS  both sexes | SR-  both sexes | SRS  bloodfed | -RS  bloodfed | SR-  bloodfed |
| --- | --- | --- | --- | --- | --- | --- | --- | --- | --- |
| SRS  only males | 1.001^*^ | V = 9.78E8^**^ p < 0.001 | V = 3.98E8 p < 0.001 | V = 2.57E7 p < 0.001 | V = 7.65E8 p < 0.001 | V = 3.17E8 p < 0.001 | V = 3.80E8 p < 0.001 | V = 6.85E8 p < 0.001 | V = 5.98E8 p < 0.001 |
| -RS  only males |  | 1.000 | V = 5.59E8 p < 0.001 | V = 1.86E8 p < 0.001 | V = 5.19E6 p < 0.001 | V = 4.05E8 p < 0.001 | V = 4.32E8 p < 0.001 | V = 1.40E8 p < 0.001 | V = 6.33E8 p < 0.001 |
| SR-  only males |  |  | 1.001 | V = 9.48E7 p < 0.001 | V = 7.49E8 p < 0.001 | V = 8.62E7 p < 0.001 | V = 2.91E8 p < 0.001 | V = 6.84E8 p < 0.001 | V = 4.46E8 p < 0.001 |
| SRS  both sexes |  |  |  | 1.007 | V = 1.38E9 p < 0.001 | V = 1.13E9 p < 0.001 | V = 5.50E8 p < 0.001 | V = 1.01E9 p < 0.001 | V = 1.18E9 p < 0.001 |
| -RS  both sexes |  |  |  |  | 1.004 | V = 9.66E8 p < 0.001 | V = 5.01E8 p < 0.001 | V = 1.93E8 p < 0.001 | V = 8.94E8 p < 0.001 |
| SR-  both sexes |  |  |  |  |  | 1.004 | V = 2.90E8 p < 0.001 | V = 7.53E8 p < 0.001 | V = 6.10E8 p < 0.001 |
| SRS  bloodfed |  |  |  |  |  |  | 1.028 | V = 1.75E9 p < 0.001 | V = 1.71E9 p < 0.001 |
| -RS  bloodfed |  |  |  |  |  |  |  | 1.023 | V = 1.51E9 p < 0.001 |
| SR-  bloodfed |  |  |  |  |  |  |  |  | 1.010 |

^*^ Values in the diagonal are the mean $N_{R}/N_{0}$ of scenarios calculated from all GSA simulations.

^**^ Wilcoxon signed rank tests with Bonferroni correction.

**Table S18.** Post-hoc pairwise comparisons of proportion of integrated VC ($p_{VC}$) among model scenarios in the two-locus Mendelian model

| $\bar{p_{VC}}$ | SRS  only males | -RS  only males | SR-  only males | SRS  both sexes | -RS  both sexes | SR-  both sexes | SRS  bloodfed | -RS  bloodfed | SR-  bloodfed |
| --- | --- | --- | --- | --- | --- | --- | --- | --- | --- |
| SRS  only males | 0.967^*^ | V = 4.98E9^**^ p < 0.001 | V = 4.96E9 p < 0.001 | V = 2.77E9 p < 0.001 | V = 4.87E9 p < 0.001 | V = 4.76E9 p < 0.001 | V = 4.44E9 p < 0.001 | V = 4.86E9 p < 0.001 | V = 4.79E9 p < 0.001 |
| -RS  only males |  | 0.936 | V = 1.24E8 p < 0.001 | V = 1.58E7 p < 0.001 | V = 3.95E9 p < 0.001 | V = 2.74E8 p < 0.001 | V = 1.98E9 p < 0.001 | V = 4.81E9 p < 0.001 | V = 2.26E9 p < 0.001 |
| SR-  only males |  |  | 0.954 | V = 1.06E8 p < 0.001 | V = 4.73E9 p < 0.001 | V = 3.82E9 p < 0.001 | V = 3.87E9 p < 0.001 | V = 4.85E9 p < 0.001 | V = 4.44E9 p < 0.001 |
| SRS  both sexes |  |  |  | 0.969 | V = 4.95E9 p < 0.001 | V = 4.94E9 p < 0.001 | V = 4.52E9 p < 0.001 | V = 4.89E9 p < 0.001 | V = 4.82E9 p < 0.001 |
| -RS  both sexes |  |  |  |  | 0.933 | V = 2.13E8 p < 0.001 | V = 1.71E9 p < 0.001 | V = 4.83E9 p < 0.001 | V = 2.08E9 p < 0.001 |
| SR-  both sexes |  |  |  |  |  | 0.952 | V = 3.72E9 p < 0.001 | V = 4.87E9 p < 0.001 | V = 4.33E9 p < 0.001 |
| SRS  bloodfed |  |  |  |  |  |  | 0.943 | V = 4.93E9 p < 0.001 | V = 2.71E9 p < 0.001 |
| -RS  bloodfed |  |  |  |  |  |  |  | 0.880 | V = 1.56E8 p < 0.001 |
| SR-  bloodfed |  |  |  |  |  |  |  |  | 0.931 |

^*^ Values in the diagonal are the mean $p_{VC}$ of scenarios calculated from all GSA simulations.

^**^ Wilcoxon signed rank tests with Bonferroni correction.
